# Prediction of prognostic signatures in triple-negative breast cancer based on the differential expression analysis via NanoString nCounter immune panel

**DOI:** 10.1186/s12885-020-07399-8

**Published:** 2020-11-02

**Authors:** Gyeong Back Lim, Young-Ae Kim, Jeong-Han Seo, Hee Jin Lee, Gyungyub Gong, Sung Hee Park

**Affiliations:** 1 Department of Bioinformatics and Life Science, Soongsil University, Seoul, 06978 Republic of Korea; 2 Department of Pathology, Asan Medical Center, University of Ulsan College of Medicine, Seoul, 05505 Republic of Korea; 3 Foundation of University-Industry Cooperation, Soongsil University, Seoul, 06978 Republic of Korea

**Keywords:** Triple negative breast cancer, Prognostic marker, NanoString nCounter immune panel, Differential expression, Pathological complete response, Relapse, Prediction model, Random Forest

## Abstract

**Background:**

Triple-Negative Breast Cancer (TNBC) is an aggressive and complex subtype of breast cancer. The current biomarkers used in the context of breast cancer treatment are highly dependent on the targeting of oestrogen receptor, progesterone receptor, or HER2, resulting in treatment failure and disease recurrence and creating clinical challenges. Thus, there is still a crucial need for the improvement of TNBC treatment; the discovery of effective biomarkers that can be easily translated to the clinics is essential.

**Methods:**

We report an approach for the discovery of biomarkers that can predict tumour relapse and pathologic complete response (pCR) in TNBC on the basis of mRNA expression quantified using the NanoString nCounter Immunology Panel. To overcome the limited sample size, prediction models based on random Forest were constructed using the differentially expressed genes (DEGs) as selected features. We also evaluated the differences between pre- and post-treatment groups aiming for the combinatorial assessment of pCR and relapse using additive models in edgeR.

**Results:**

We identify nine and 13 DEGs strongly associated with pCR and relapse, respectively, from 579 immune genes in a small number of samples (*n* = 55) using edgeR. An additive model for the comparison of pre- and post-treatment groups via the adjustment of the independent subject in the relapse group revealed associations for 41 genes. Comprehensive analysis indicated that our prediction models outperformed those constructed using features extracted from the existing feature selection model Elastic Net in terms of accuracy. The prediction models were assessed using a randomization test to validate the robustness (empirical *P* for the model of pCR = 0.015 and empirical *P* for the model of relapse = 0.018). Furthermore, three DEGs (FCER1A, EDNRB, and TGFBI) in the model of relapse showed prognostic significance for predicting the survival of patients with cancer through Cox proportional hazards regression model-based survival analysis.

**Conclusion:**

Gene expression quantified via the NanoString nCounter Immunology Panel can be seamlessly analysed using edgeR, even considering small sample sizes. Our approach provides a scalable framework that can easily be applied for the discovery of biomarkers based on the NanoString nCounter Immunology Panel.

**Data availability:**

The source code will be available from github at https://github.com/sungheep/nanostring.

## Background

The subtypes of breast cancer have distinct pathological features and clinical implications and primarily include hormone receptor-positive breast cancer, HER2-positive breast cancer, and triple-negative breast cancer (TNBC). Of note, breast cancer classification depends on protein or gene expression profiling; importantly, it provides helpful information for prognosis establishment and adoption of treatment strategies. Breast cancer therapy involves drugs that target oestrogen, progesterone, and HER2 receptors expressed on hormone receptor-positive and HER2-positive breast cancer cells, respectively [1]. However, TNBC does not respond to these therapies, including tamoxifen or trastuzumab, as no specific receptors are expressed in TNBC. TNBC is characterized by its invasiveness, widespread metastasis, and high post-treatment relapse rates, although many studies have attempted to predict the aetiology, response to treatment, and prognosis of TNBC [2]. In this study, we aimed to identify prognostic biomarkers for TNBC to facilitate improvements in the current treatment approaches.

The NanoString nCounter Analysis System is composed of a prep station and a digital analyser and is used to quantify gene expression levels and chromosome variations. This system identifies target genes using specific 100-mer probes and simultaneously analyses 800 genes. A key advantage of the NanoString technology with respect to next-generation sequencing (NGS) is the absence of an amplification step and the ability to directly quantify target molecules, thus preventing artificial amplification bias.

Since 2014, numerous studies have attempted to analyse TNBC using a NanoString nCounter Immunology Panel [3–6]. Most expression analyses studies using NanoString nCounter data [7] focused on statistical analyses and clustering analyses with gene heat maps similar to those used in microarray data analysis. Such statistical analyses included the Mann-Whitney U test [5, 6, 8, 9], the *t*-test, or the analysis of variance [3, 6], as well as the Fisher’s exact test [5, 9], Spearman’s correlation [5, 10], and negative binomial distribution [8]. Using the NanoString nCounter Immunology Panel, gene expression can be quantified as the counts measured in a manner similar to that used to quantify expression on the NGS platform; the statistical methods generally used are also used in microarray data analysis.

We developed an approach for biomarker discovery which predicts relapse and pathological complete responses (pCR) after neoadjuvant chemotherapy in TNBC as per learning prediction models using random Forest with features selected via the analysing of differential gene expression using edgeR. The data was obtained using the NanoString nCounter Immunology Panel. This study takes advantage of the prognostic model for predicting tumour relapse and pCR with a small sample size via the application of edgeR to assess differential gene expression, which is suitable for nCounter Immunology Panel analysis in feature selection.

## Methods

Paraffin-embedded tissue biopsy samples from 55 TNBC patients treated with anthracycline and taxane-based neoadjuvant chemotherapy (or surgery) from 2010 to 2012 at the Asan Medical Centre. The study was approved by the Institutional Review Board of the Asan Medical Center (IRB No. 2013–0866). We used whole sections of biopsy tissues with usually 4–5 cores. Importantly, when we cut the FFPE blocks, we used different blades for each sample after cleaning the microtome with 70% ethanol to prevent cross-contamination. So, our experiment is free from cross-contamination. The samples were processed using the GX Human Immunology V2kit (NanoString Technologies, Seattle, WA, USA) for NanoString nCounter Gene expression analysis of a total of 579 immunology-related human genes [5]. Among the 55 cases, a NanoString nCounter assay was performed using specimens from 14 patients, including 6 cases of pCR, after treatment. The clinical data of the patients (Additional file 5: Table S1), including the survival time, survival parameters, and chemotherapeutic responsiveness (pCR, residual cancer burden, Miller Payne grade), were collected. Thereafter, a two-row count matrix was constructed; one row showed the 55 samples and the other showed the 14 samples collected after treatment.

We have subjected the same samples from 56 patients in our data set to anti-CD3, *−*CD8, and -CD20 immunohistochemistry (IHC) staining. Correlations between IHC staining intensity and gene expression levels were then assessed using the NanoString nCounter platform and are summarized (Additional file 5: Table S2). Spearman correlation ranged from 0.623–0.761. We analysed the spearman correlation between the expression of five genes (CD3D, CD3E, CD8A, CD8B, and CD20) quantified using the NanoString nCounter platform and the immunohistochemistry staining results for CD3+, CD8+, and CD20+ cells. Of note, in the CD3+, CD8+, and CD20+ cells, the intensity of immunohistochemistry stainings was highly correlated with the expression of T and B cell markers. With respect to this observation, gene expression quantified via the NanoString nCounter platform overall highly correlated with IHC stainings.

### Feature selection

Our cohort contained a small number of samples (*n* = 55). Feature selection methods including the Elastic Net (EN) may not guarantee high accuracy in the context of small data sets. The NanoString nCounter platform allows a count format, unlike microarray analysis, for quantifying gene expression on an NGS platform without amplification. We used edgeR, which identifies differentially expressed genes (DEGs) under different conditions, using a negative binomial statistical model fitted to these observed counts [11]. EdgeR can identify DEGs in a relatively small number of samples (*n* ≥ 2).

**Fig. 1:**
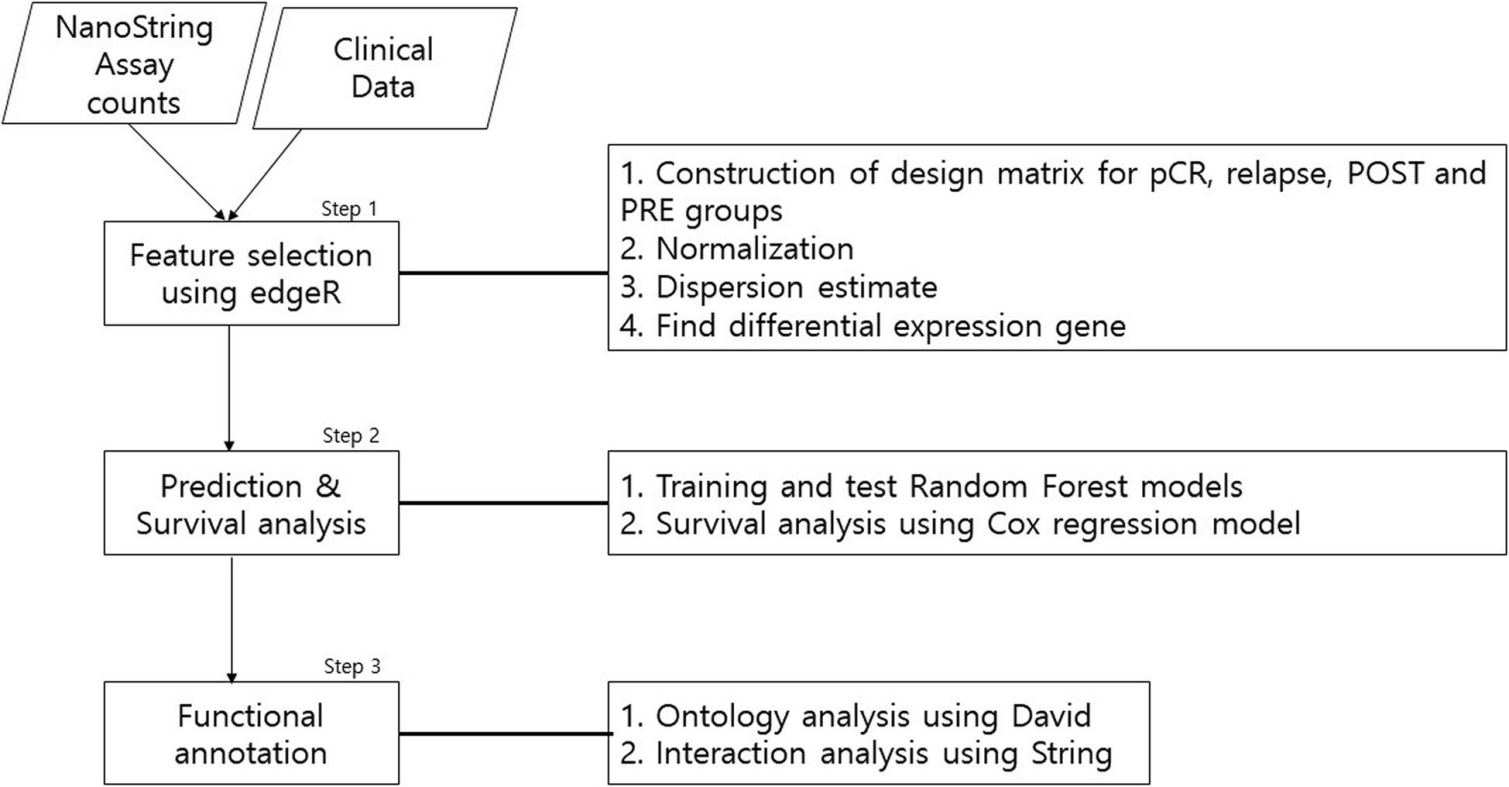
A Work flow diagram

DEGs identified by edgeR were considered as features to develop prediction models and for survival analysis (Fig. 1). Functional annotations were performed using DAVID to evaluate the significant signature genes identified via the edgeR analysis.

#### Feature selection using edgeR

We converted raw count data to log counts per million, normalized the counts using the trimmed mean of M-values in edgeR, and estimated the dispersions. DEGs were analysed to select features useful in prediction models of pCR and relapse. After setting the design matrix (e.g., pCR = 1 vs non-pCR = 0, conditions for the pCR model, and relapse = 1 and non-relapse = 0 for the relapse model), significant DEGs were identified at a false discovery rate (FDR) < 0.05.

To identify DEGs using edgeR for comparisons pre- and post-treatment (PRE and POST, respectively), we first divided samples from biopsies performed prior to neoadjuvant chemotherapy (or operation specimens) and those collected after neoadjuvant chemotherapy. Fourteen paired samples (pre- and post-treatment) were stratified based on relapse and pCR (Additional file 1: Figure S1). We conducted three DEG tests to evaluate combinations of disease status (relapse and pCR) and treatments (e.g., PRE and POST). The three combinations were POST and pCR, POST and relapse, and PRE/POST and relapse. For a DEG test, an additive model was designed and applied to detect genes differentially expressed in samples obtained at POST and PRE, adjusting for the baseline difference between patients. In the combination POST and relapse, two samples, NS010 in the non-relapse group and NS032 in the relapse group, were omitted because of heterogeneity in their multidimensional scaling plot (Additional file 1: Figure S2). After the design matrix was constructed, normalization was performed and dispersions were estimated. Significant DEGs were identified at an FDR < 0.05.

#### Feature selection using lasso and elastic net

We compared the performance of our approach to that of EN to determine whether DEG selection using edgeR in our method was more appropriate for the selection of features for prediction models. We used “glmnet” from the R package to implement EN.

### Prediction model

#### Data pre-processing

Global normalization was performed by dividing the raw count data into sequencing depths, and normalization was carried out considering relatively different gene lengths. We subsequently stratified the dataset into a ratio of 7: 3 for the training and test sets, with pCR and relapse groups as class labels. Datasets for each pCR and relapse set are shown in Table 1.

**Table 1:** Dataset

	Training	Test
^a^non-pCR	26	11
^b^pCR	12	6
^c^non-relapase	22	13
^d^relapse	16	4

^a, b^: The number of patients in non-pCR and pCR corresponding to training and test groups

^c, d^: The number of patents in non-relapse and relapse corresponding to training and test groups

#### Prediction using the random Forest models

Considering the DEGs in pCR and relapse sets, we trained random Forest models to predict pCR and relapse. One limitation of this classification is that greater sample imbalance leads to more biased results in larger sample groups [12]. To compensate for this problem, we performed oversampling using SMOTE. After oversampling, the small group was matched at a ratio of 1:1 to the large group, and the random Forest model was trained. We used sklearn’s GridSearchCV in the hyperparameter optimization process to optimise model performance. Five hyperparameters (n_estimator, max_feature, max_depth, min_samples_split, and min_samples_leaf) were used among several hyperparameters to maximize model performance, and a 5-fold cross-validation was performed to avoid overfitting caused by the small number of samples.

#### Evaluation

To evaluate the performance of the predictive models, we used AUC metrics determined via receiver operating characteristic analysis. In addition, the positive prediction value (PPV: TP/TP + FP) was used, which is widely employed to assess the performance of a diagnostic test.

#### Randomization test

Hypothesis tests were conducted to determine whether the accuracy of the pCR and relapse prediction models resulted from chance events. Empirical *P* values were calculated as per 1000 permutations using the same number of features and same hyperparameter optimization used in our prediction models and rerunning a prediction model within each permuted dataset. The AUC and PPV of a prediction model for a permuted dataset were assessed with respect to their accuracy.

#### Survival analysis using cox proportional hazard model

We used the Cox proportional hazard model (Cox proportional hazards regression model), which is a survival analysis model for multiple variables, using all genes in the prediction models [13]. For prognostic prediction, significant genes (*P* < 0.05) were identified as per survival analysis of the Cox proportional hazards regression models.

#### Functional enrichment analysis

Functional analysis was performed for DEGs extracted via edgeR using DAVID (Database for Annotation, Visualisation and Integrated Discovery, http://david.abcc.ncifcrf.gov).

#### Protein interactions using STRING

To analyse the pathological significance of breast cancers of feature-derived DEGs, network analysis was performed with STRING (biological database and visualization for network analysis); https://string-db.org/cgi/input.pl?sessionId=Sdm7S6Tqzlf4&input_page_show_search=on

## Results

The overall workflow of our study is shown in Fig. 1. The analysis of differentially expressed genes (DEGs) using edgeR for feature selection, the construction of prediction models, and the survival analysis are depicted. Functional analysis was performed using DAVID for signature genes, while the biological interpretation was carried out through a literature survey.

### Feature selection

Differential gene expression analysis was performed using edgeR for pCR and relapse conditions. Nine and 13 DEGs associated with pCR and relapse, respectively, were identified (Table 2). We also analysed the differences between pre- and post-treatment paired samples upon combinatorial assessment of pCR and relapse. PRE refers to biopsies performed prior to neoadjuvant chemotherapy (*n* = 55), whereas POST indicates operations performed after neoadjuvant chemotherapy (*n* = 14, including 6 pCR cases in Additional file 1: Figure S1). We evaluated multiple experimental factors. Three tests were performed to evaluate the combinations of disease status (relapse and pCR) and treatments (e.g., PRE or POST); i.e., comparison of a POST and pCR group with a POST and relapse group, comparison of a POST and relapse group with a POST and non-relapse group, and comparison of POST and PRE relapse groups, which revealed significant DEGs. One (e.g., *KLRG2*) and three (*HLA-DQA1*, *HLA-DQB1*, and *CEACAM6*) genes were significantly associated with the combination of POST treatment and pCR and relapse groups, respectively (Table 3). An additive model for the comparison of PRE to POSTgroups by adjusting the independent subject in the relapse group revealed associations for 41 genes (Additional file 1: Table S1). *KLRG2* and *CEACAM6* were included as DEGs in the PRE and POST comparison in the relapse group. Differences in these DEGs between PRE and POST groups were much greater than those between the relapse and non-relapse groups (Additional file 2: Figure S1).

**Table 2:** DEGs in pCR and relapse groups

Gene	^a^*P*-value
^b^pCR DEG	
IL2RA	2.34E-07
CCL5	1.17E-06
SELE	1.71E-04
CCL20	1.67E-05
FCER1A	7.49E-04
CD1A	8.26E-05
HAMP	3.35E-04
CD7	5.12E-04
C4A.B	1.34E-04
^c^RELAPSE DEG	
CCL5	9.49E-06
vCCL7	9.85E-06
TNFSF13B	5.80E-05
CSF2RB	1.60E-04
CLEC4E	2.73E-04
CCL8	3.58E-04
SELE	3.97E-04
EDNRB	4.34E-04
IL17B	5.75E-04
IL2RA	7.44E-04
FCER1A	8.09E-04
TGFBI	1.07E-03
GZMB	1.12E-03

Only genes at FDR < 0.05 after multiple hypothesis testing are presented

^a^: Original *P* value calculated in edgeR

^b, c^: DEGs in pCR and relapse conditions respectively

**Table 3:** DEGs in combinations of pCR and relapse with POST treatment

Gene	^a^*P* value
**DEGs for a**^**b**^**pCR and POST group**	
KLRG2	0.000048
**DEGs for a** ^**c**^**relapse and POST group**	
HLA-DQA1	1.45E-25
HLA-DQB1	6.26E-19
CEACAM6	2.36E-04

^a^: Original *P* value calculated in edgeR

^b^: Combination of a POST and pCR group compared with a POST and relapse group

^c^: Combination of a POST and relapse group with a POST and non-relapse group

### Prediction of pCR and relapse

**Fig. 2:**
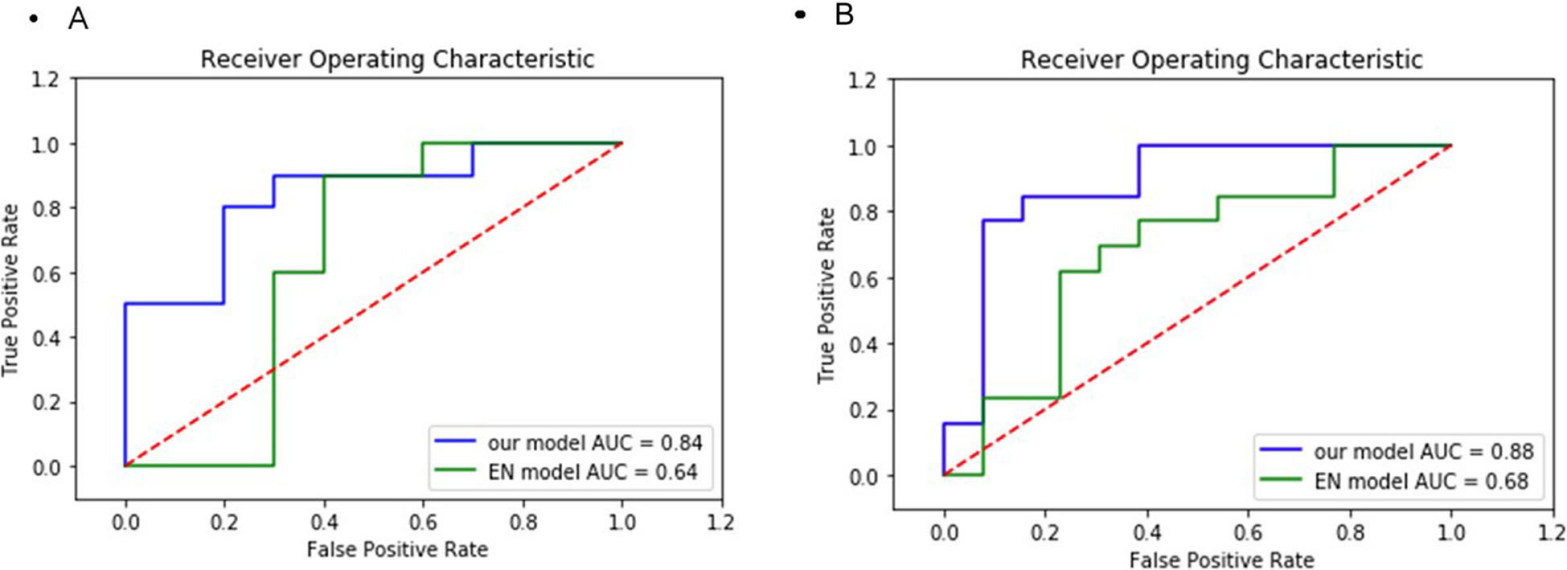
Comparison of our prediction models with EN-based prediction models. **a** AUCs between ours and the EN-based prediction model of pCR. Blue represents our RF model of pCR constructed on the features selected using edgeR, which is more robust than that the model represented in green, based on features selected using EN. **b** AUCs between ours and EN-based prediction model of relapse. Blue represents our RF model of relapse constructed on the features selected using edgeR

The performance of our predictive models for pCR and relapse, based on the random Forest method, showed an area under the curve (AUC) of 0.84 and a positive predictive value (PPV) of 0.7 for the pCR predictive model (Table 4). For the relapse predictive model, the AUC was 0.88 and PPV was 0.69 (Table 4). We compared the performance of these models to those of the classic feature selection method, Elastic Net (EN) (Fig. 2 and Table 4). The predictive models based on EN for pCR showed an AUC and PPV of 0.64 and 0, respectively, whereas our pCR analysis predicted these values to be 0.84 and 0.7, respectively. The PPV of our pCR model was not comparable to that of EN; the performance was low for our immune panel data and did not control the false-positive rate. The relapse model based on EN predicted an AUC and PPV of 0.68 and 0.23, respectively. Comprehensive analysis indicated that our model outperformed the EN with respect to the type I error rate (Table 4). Prediction models involving combinatorial assessment of PRE and POST groups for pCR and relapse models (Table 3) were not constructed, as the sample size of POST is limited.

**Table 4:** Performance comparison with our model and Elastic Net model

Model	^a^AUC	^b^PPV
^c^pCR model	0.84	0.7
^d^ RELAPSE model	0.88	0.69
^E^EN pCR model	0.64	0
^f^EN RELAPSE model	0.68	0.23

^a^AUC: Receiver Operating Characteristic Area Under Curve

^b^PPV: Predictive Positive Value (TP / TP + FP)

^c^Our model pCR: Random Forest analysis using pCR DEG.

^d^Our model RELAPSE: Random Forest analysis using RELAPSE DEG.

^e^EN model pCR: Random Forest analysis using EN pCR genes (alpha value < 0.95)

^f^EN model RELAPSE: Random Forest analysis using EN RELAPSE genes (alpha value < 0.2)

**Fig. 3:**
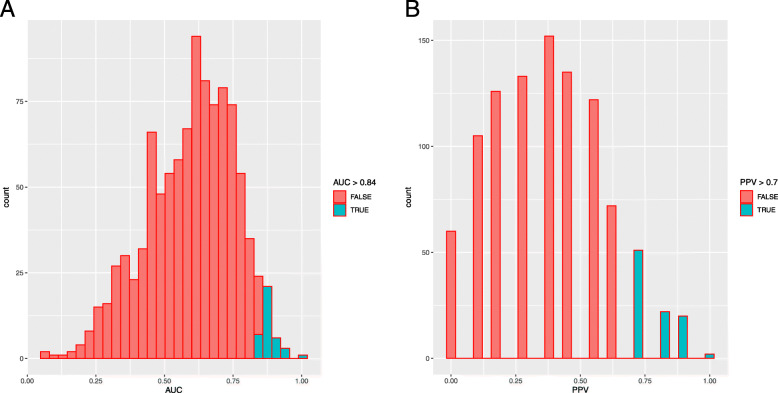
A randomization test for pCR. Plots present the distributions of the AUC and PPV values for all 1000 permutations. Histograms represent (**a**) AUCs and (**b**) PPV of permutations. Red represents AUC or PPV of permutations below (Table 4) whilst green represents permutations superior to our thresholds

**Fig. 4:**
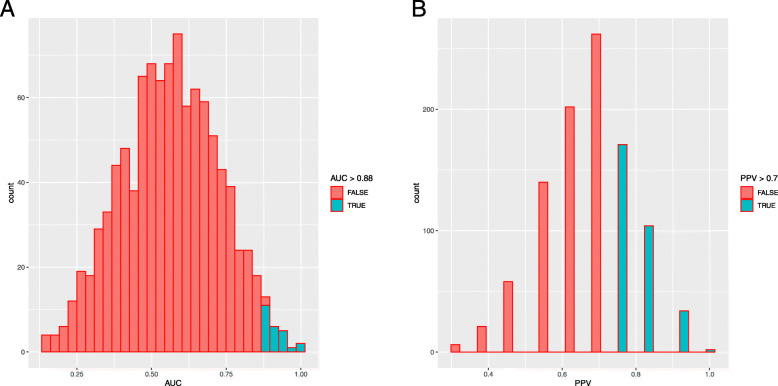
A randomization test for relapse. Plots present the distribution of the AUC and PPV values for all 1000 permutations. Histograms represent (**a**) AUC and (**b**) PPV of permutations. Red represents AUC or PPV below (Table 4) whilst green represents permutations superior to our thresholds

We performed a randomization analysis to investigate whether the significant DEGs in the prediction models were identified by chance. For pCR analysis based on random feature selection, 15 cases met our cut-off for accuracy (empirical *P* = 0.015) to reject the null hypothesis. The models were superior to our prediction model of pCR with respect to the AUC (> 0.84) and PPV (> 0.7) (Fig. 3). For relapse, 18 cases (*P* = 0.018) displayed a superior AUC (> 0.88) and PPV (> 0.69) than that of our prediction model of relapse (Fig. 4).

### Survival analysis

**Fig. 5:**
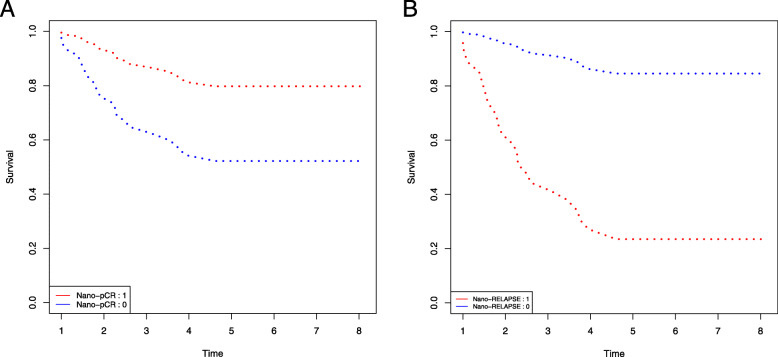
Survival analysis using the Cox proportional hazards regression model. **a** Stands for the cox model built on pCR genes. Red indicates pCR and blue non-pCR. **b** Stands for the cox model built on relapse genes. Red represents relapse and blue non-relapse

We used the Cox proportional hazards regression model to evaluate if the nine and 13 genes in the prediction models of pCR and relapse affected patient survival to determine their value as prognostic markers. In the pCR survival plot (Fig. 5a), a significant difference was observed between pCR and non-pCR patients during the first 4 years. In contrast to pCR survival analysis, recurrence-free survival was observed among patients showing expression changes in 13 DEGs, with very poor survival during the first 4 years (Fig. 5b). TNBC relapse was predicted to be 80% in earlier years, decreasing to less than 10% at 4 years, indicating that the DEGs are associated with a high risk of TNBC relapse during the first 4 years. The prognostic impact of these DEGs was constant after exceeding this point.

**Fig. 6:**
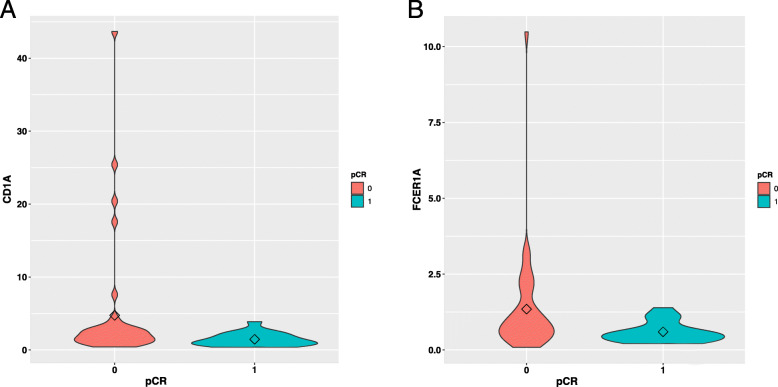
Violin plots for significant genes in the Cox model of pCR. **a** and **b** represent *CD1A* and *FCER1A*, respectively. *CD1A*, and *FCER1A* have high expression in non-pCR

**Fig. 7:**
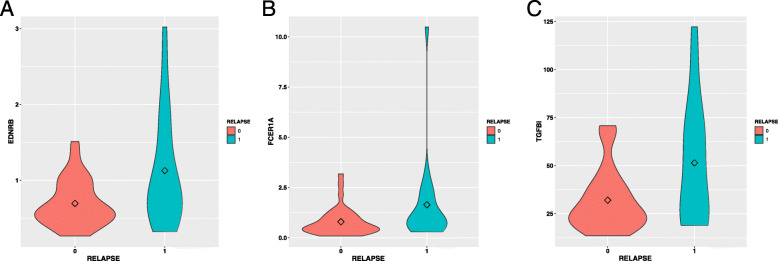
Violin plots for significant genes in the Cox model of relapse. Violin plots for three significant genes in the Cox proportional hazards regression model. **a** Corresponds to *EDNRB* with high expression at relapse, **b** corresponds to *FCER1A* with high expression at relapse, and **c** corresponds to *TGFBI* with high expression at relapse

Two significant genes (*FCER1A* and *CD1A*) in the Cox proportional hazard regression model (Table 5) were downregulated in the pCR group (Fig. 6). The violin plots for the remaining genes in the pCR model are shown in Additional file 3. In the relapse group, three genes (i.e. *FCER1A*, *EDNRB*, and *TGFBI*) were significantly up-regulated (*P* < 0.05; Table 6 and Fig. 7). The violin plots for the remaining 10 genes in the relapse model are shown in Additional file 4.

**Table 5:** Significant genes in overall survival analysis for the pCR model

Gene	^a^Hazard Ratio(95%CI)	^b^*P*-value
CD1A	0.103439	0.00755
FCER1A	0.47464	0.00128

^a^: The Hazard Ratio calculated in the Cox proportional hazards regression model. A Hazard ratio lower than 1 indicates non-risk factor; a ratio higher than 1 indicates risk factor

^b^: Original *P* value (< 0.05) calculated in the Cox proportional hazards regression model

**Table 6:** Significant genes in disease free survival analysis for the relapse model

Gene	^a^Hazard Ratio(95%CI)	^b^*P* value
FCER1A	0.14087	0.0458
EDNRB	0.35933	0.0337
TGFBI	0.52611	0.0262

^a^: The Hazard Ratio calculated in the Cox proportional hazards regression model. A Hazard ratio lower than 1 indicates non-risk factor; a ratio higher than 1 indicates risk factor

^b^: Original *P* value (< 0.05) calculated in the Cox proportional hazards regression model

### Interpretation of functional annotations and network analysis

Significant DEGs were evaluated via functional enrichment analysis using the DAVID functional annotation tool. The results of functional annotation are summarised in Table 7 (e.g., nine genes for pCR and 13 genes for relapse) and showed that DEGs for pCR and relapse had similar functions. Both were enriched in cytokine-cytokine interactions. As indicated by KEGG pathway annotation, cytokine-cytokine receptor interactions play an important role in cell proliferation and differentiation, survival, and pathogen resistance; the release of cytokines in response to infection, inflammation, and immunity can inhibit tumorigenesis and cancer progression [9].

**Table 7:** Functional enrichment analysis for pCR and RELAPSE models

Category	Term	Genes	^a^*P* Value
^b^pCR			
GOTERM_BP_DIRECT	GO:0006955 ~ immune response	IL2RA, CCL20, HAMP, CD1A, CCL5, CD7	1.95E-07
UP_SEQ_FEATURE	disulfide bond	FCER1A, IL2RA, CCL20, HAMP, CD1A, CCL5, SELE, CD7	1.36E-06
UP_SEQ_FEATURE	signal peptide	FCER1A, IL2RA, CCL20, HAMP, CD1A, CCL5, SELE, CD7	3.57E-06
UP_KEYWORDS	Disulfide bond	FCER1A, IL2RA, CCL20, HAMP, CD1A, CCL5, SELE, CD7	3.58E-06
UP_KEYWORDS	Signal	FCER1A, IL2RA, CCL20, HAMP, CD1A, CCL5, SELE, CD7	1.37E-05
KEGG_PATHWAY	hsa04640:Hematopoietic cell lineage	IL2RA, CD1A, CD7	0.002295
KEGG_PATHWAY	hsa04668:TNF signaling pathway	CCL20, CCL5, SELE	0.003452
KEGG_PATHWAY	hsa04060:Cytokine-cytokine receptor interaction	IL2RA, CCL20, CCL5	0.016968
KEGG_PATHWAY	hsa05323:Rheumatoid arthritis	CCL20, CCL5	0.074368
^c^RELAPSE			
UP_SEQ_FEATURE	disulfide bond	FCER1A, IL2RA, CCL8, GZMB, CCL5, CCL7, EDNRB, IL17B, TNFSF13B, CLEC4E, TGFBI, CSF2RB, SELE	8.75E-11
UP_KEYWORDS	Disulfide bond	FCER1A, IL2RA, CCL8, GZMB, CCL5, CCL7, EDNRB, IL17B, TNFSF13B, CLEC4E, TGFBI, CSF2RB, SELE	4.58E-10
UP_SEQ_FEATURE	signal peptide	FCER1A, EDNRB, IL17B, IL2RA, TGFBI, CCL8, CSF2RB, GZMB, CCL5, SELE, CCL7	7.83E-07
UP_KEYWORDS	Cytokine	IL17B, TNFSF13B, CCL8, CCL5, CCL7	3.29E-06
GOTERM_BP_DIRECT	GO:0006954 ~ inflammatory response	IL17B, IL2RA, CCL8, CCL5, SELE, CCL7	3.97E-06
UP_KEYWORDS	Signal	FCER1A, EDNRB, IL17B, IL2RA, TGFBI, CCL8, CSF2RB, GZMB, CCL5, SELE, CCL7	4.97E-06
UP_KEYWORDS	Glycoprotein	FCER1A, EDNRB, IL17B, IL2RA, CLEC4E, TNFSF13B, CSF2RB, GZMB, CCL5, SELE, CCL7	1.17E-05
KEGG_PATHWAY	hsa04060:Cytokine-cytokine receptor interaction	IL17B, IL2RA, TNFSF13B, CCL8, CSF2RB, CCL5, CCL7	7.28E-07
KEGG_PATHWAY	hsa04062:Chemokine signaling pathway	CCL8, CCL5, CCL7	0.034067

Only annotations with a *FDR* < 0.05 after multiple hypothesis testing are presented from DAVID outputs

^a^: Original *P* value (FDR < 0.05) calculated in DAVID

**Fig. 8:**
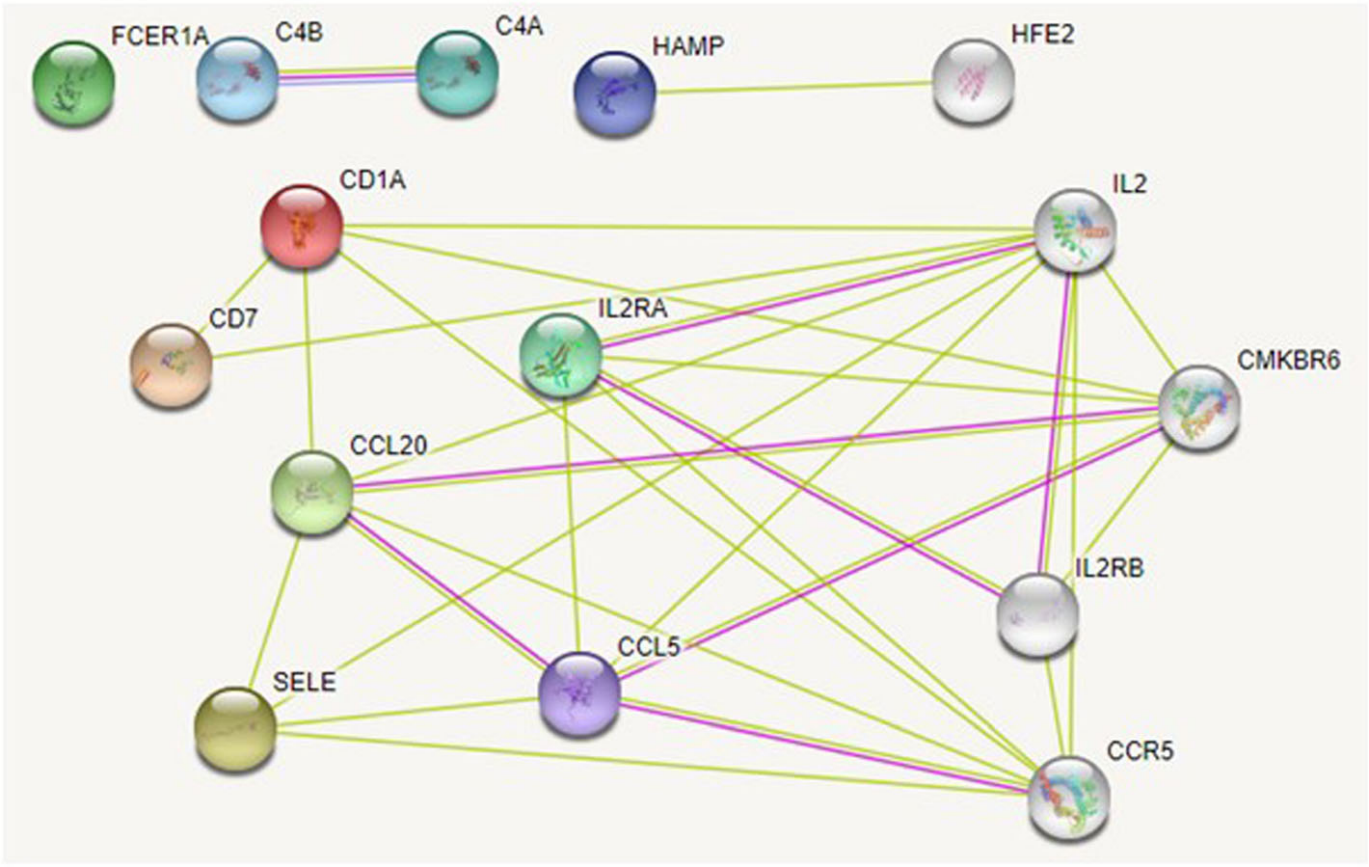
An interaction network for genes in the pCR model. The interaction network is derived from STRING for the nine significant genes in the pCR model. *FCER1A* did not form any interaction as per the STRING Database. Red represents interactions derived from gene fusion, purple from experiments, and yellow from text mining

**Fig. 9:**
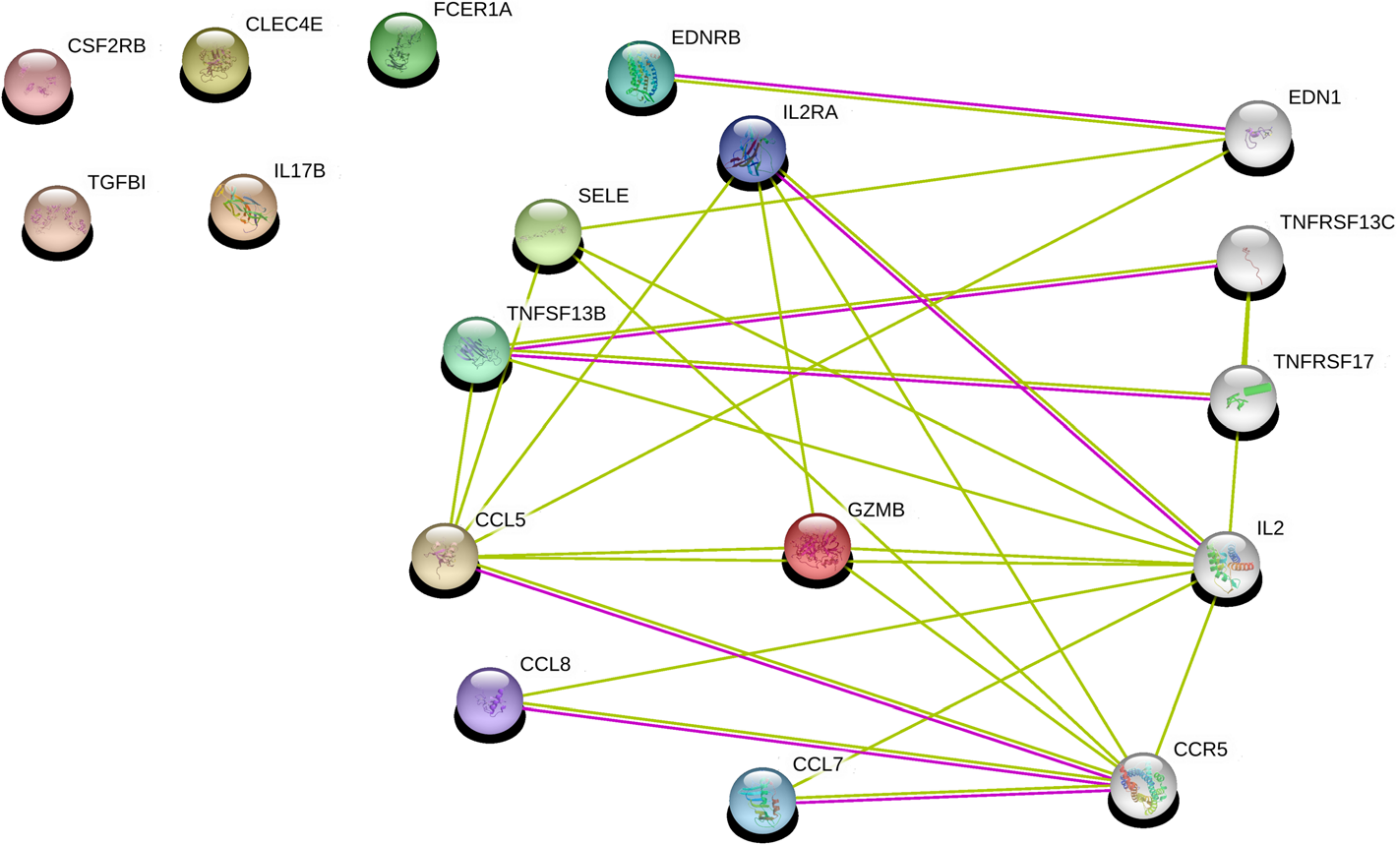
An interaction network for genes in the relapse model. Of 13 genes in the relapse model, eight genes formed interactions. Five genes on the top left corner did not form any interaction as per the STRING database. The interaction network is derived from STRING for the nine significant genes. Red represents interactions derived from gene fusion, purple from experiments, and yellow from text mining

Network analysis was performed using the STRING database. Gene interactions were determined using the STRING database through experiments, text mining, and gene fusions. Among the DEGs in the pCR group, CCL*5*, *CCL20*, *CD1A*, and *IL2RA* interact with *CCR5* and *CCR6*, alias *CMKBR6* (Fig. 8). Gene-gene interactions were detected in the relapse group (Fig. 9); *CCL5*, *GZMB*, *IL2RA*, *SELE*, and *CCL8* interacted with *CCR5* in both the pCR and relapse groups and were associated with tumour progression [14], and metastasis [10]. In patients with breast cancer, *CCR5* and its ligand *CCL5* were found to be upregulated among DEGs in the pCR group [10]. Furthermore, *CCR5* is a novel therapeutic target for metastatic cancer, and recent clinical trials have targeted this gene in breast and colon cancer.

*CCR6* interacted with *CCL20* in the pCR group and is a receptor of *CCL20*; overexpression of *CCL20* augments mitogen-activated protein kinase and protein kinase C signalling, resulting in tumour progression [15–17]. Significant enrichment in molecular functions determined by Gene Ontology analysis of genes and their interaction partners in the STRING database are summarised in Table 8.

**Table 8:** Molecular function enrichment of genes in interaction networks

pCR		RELAPSE	
GO-term	term description	GO-term	term description
GO:0019955	cytokine binding	GO:0005125	cytokine activity
GO:0004896	cytokine receptor activity	GO:0001664	G protein-coupled receptor binding
GO:0004911	interleukin-2 receptor activity	GO:0005102	signaling receptor binding
GO:0019976	interleukin-2 binding	GO:0005126	cytokine receptor binding
GO:0098772	molecular function regulator	GO:0048020	CCR chemokine receptor binding
GO:0005515	protein binding	GO:0098772	molecular function regulator
GO:0016493	C-C chemokine receptor activity	GO:0008009	chemokine activity
GO:0019957	C-C chemokine binding	GO:0031726	CCR1 chemokine receptor binding
GO:0038023	signaling receptor activity	GO:0016004	phospholipase activator activity
GO:0001848	complement binding	GO:0004896	cytokine receptor activity
GO:0004435	phosphatidylinositol phospholipase C activity	GO:0038023	signaling receptor activity
GO:0048018	receptor ligand activity	GO:0004435	phosphatidylinositol phospholipase C activity
GO:0015026	coreceptor activity	GO:0005515	protein binding
GO:0048020	CCR chemokine receptor binding	GO:0005088	Ras guanyl-nucleotide exchange factor activity
GO:0005125	cytokine activity	GO:0030246	carbohydrate binding
GO:0008009	chemokine activity	GO:0019209	kinase activator activity
GO:0005088	Ras guanyl-nucleotide exchange factor activity	GO:0019955	cytokine binding
GO:0001664	G protein-coupled receptor binding	GO:0004888	transmembrane signaling receptor activity
GO:0005126	cytokine receptor binding	GO:0008528	G protein-coupled peptide receptor activity
GO:0030246	carbohydrate binding	GO:0008201	heparin binding
GO:0004888	transmembrane signaling receptor activity	GO:0008047	enzyme activator activity
GO:0019209	kinase activator activity		
GO:0005102	signaling receptor binding		
GO:0005488	binding		
GO:0030234	enzyme regulator activity		
GO:0004866	endopeptidase inhibitor activity		
GO:0004252	serine-type endopeptidase activity		

Molecular Function analysis presents. *GO* Gene Ontology. GO terms at FDR < 0.05 present

## Discussion

In this study, we developed a novel biomarker discovery approach in the context of pCR and relapse in TNBC. We used edgeR for feature selection from the NanoString nCounter Immunology Panel and constructed prediction models for pCR and relapse for TNBC based on selected features using the random Forest method. Moreover, we verified the gene signatures of pCR and relapse prediction models for TNBC treatment through a literature survey.

Two significant genes (*CD1a* and *FCER1A*) related with the survival outcome in the pCR prediction model have been reported as conventional dendritic cell markers and are highly expressed in innate antigen-presenting cells infiltrating breast cancer tissues [18], which is consistent with our findings (Fig. 6). We found that low expression of conventional dendritic cell markers (*CD1a* and *FCER1A*) was associated with pCR, potentially affecting the overall patient survival in TNBC; however, a previous study [19] reported no significant association between the levels of CD1a + tumour-infiltrating dendritic cells and pCR in either the primary tumours or axillary lymph node metastasis. There has been no previous study evaluating the effects of CD1a + in dendritic cells in the context of breast cancer survival (in patients receiving neoadjuvant chemotherapy).

Of the three significant genes (*FCER1A*, *EDNRB*, and *TGFBI*) in the relapse prediction model, *EDNRB* is located on chromosome 13 and encodes a G protein-coupled receptor. *EDNRB* downregulation can prevent TNBC progression and may be a biomarker candidate for TNBC treatment efficacy prediction [20]. It has been reported that TGFBI is associated with both breast cancer inhibition [21] and progression [22]. A recent study revealed that TGFBI was upregulated in treatment-resistant TNBC MDA-MB-231/IR cells and is involved in drug and radiation resistance [23]. No previous study has reported that TGFBI is associated with recurrence-free survival of patients with TNBC. In total, three genes were considered as targeted diagnostic and treatment biomarkers for TNBC. Nevertheless, additional experimental validation of these genes as new drug targets is required.

The cost of NGS-based approaches for immune profiling remains high, despite the high demand for this method. This may result in the analysis of limited sample sizes. Most machine learning methods for feature selection are suitable for large datasets or show poor performance for small sample sizes.

## Conclusions

We described a practical approach for analysing transcriptome data generated using the NanoString nCounter Immunology platform with a matrix similar to that of an NGS platform, which currently involves statistical methods based on microarray analysis. This study provides a framework for transcriptome analysis in NGS, which can be applied to data obtained using the NanoString nCounter Immunology Panel.

## Supplementary information

**Additional file 1.** PRE_POST analysis. **Figure S1.** Data distribution in PRE and POST groups. **Figure S2.** A MDS plot for POST relapse and POST non-relapse groups. **Table S1.** DEGs for POST vs PRE in the relapse group.

**Additional file 2.** 41 DEGs for comparison of POST to PRE in relapse. **Figure S1.** Violin plots for 41 DEGs for comparison of POST versus PRE in relapse.

**Additional file 3.** Violin plots for nine DEGs in the pCR model. **Figure S1.** Violin plots of nine pCR DEGs. *IL2RA*, *CCL5*, *SELE*, *CCL20*, *CD7*, genes have high expression in pCR. *FCER1A*, *CD1A*, *HAMP, C4A.B* genes have high expression in non-pCR.

**Additional file 4.** Violin pots for 13 DEGs in the relapse model. **Figure S1.** Violin plots of 13 relapse DEGs. CCL5, CCL7, TNFSF13B, CSF2RB, CLEC4E, CCL8, SELE, IL17B, IL2RA, and GZMB genes have high expression in non-RELAPSE. *FCER1A*, *EDNRB, TGFBI* genes have high expression in relapse.

**Additional file 5.** Clinical data for all patients. Table S1: Clinical data for all patients. **Table S2.** Spearman correlation between immunohistochemistry staining and gene expression quantified using the NanoString nCounter platform.

## Data Availability

The dataset supporting the conclusions of this article is available from the Gene Expression Omnibus - accession ID GSE: GSE143222.
