# Supplementary material for: Prediction of prognostic signatures in triple-negative breast cancer based on the differential expression analysis via NanoString nCounter immune panel
Source: BMC Cancer. 2020 Nov 2;20:1052. doi: 10.1186/s12885-020-07399-8 (PMC7607642; doi:10.1186/s12885-020-07399-8)
Supplement: Supplementary file 1 — Additional file 1. PRE_POST analysis. Figure S1. Data distribution in PRE and POST groups. Figure S2. A MDS plot for POST relapse and POST non-relapse groups. Table S1. DEGs for POST vs PRE in the relapse group. [file 12885_2020_7399_MOESM1_ESM.pptx]

## Slide 1
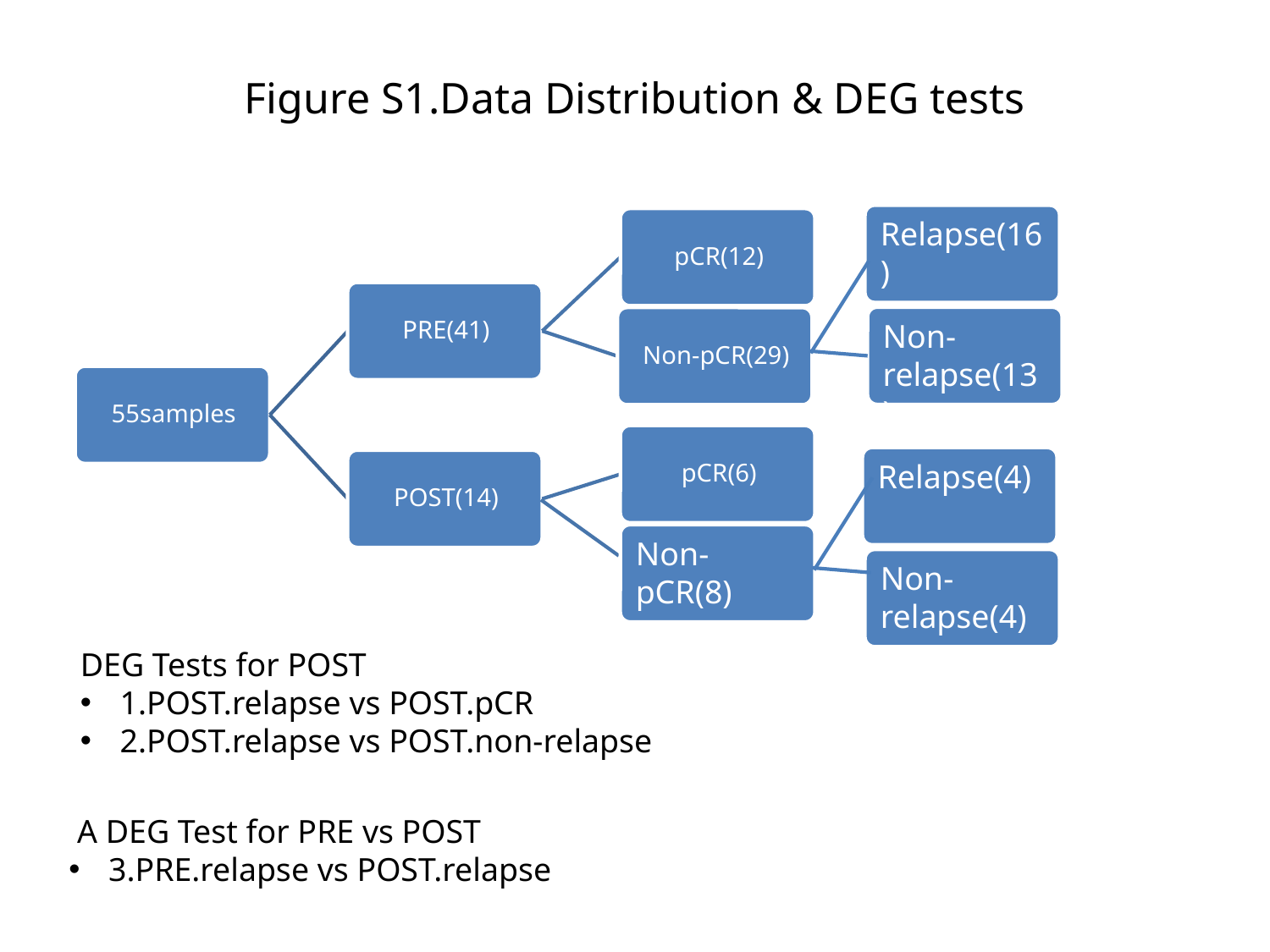

# Figure S1.Data Distribution & DEG tests
Relapse(16)
Non-relapse(13)
Relapse(4)
Non-pCR(8)
Non-relapse(4)
DEG Tests for POST
1.POST.relapse vs POST.pCR
2.POST.relapse vs POST.non-relapse
 A DEG Test for PRE vs POST
3.PRE.relapse vs POST.relapse

## Slide 2
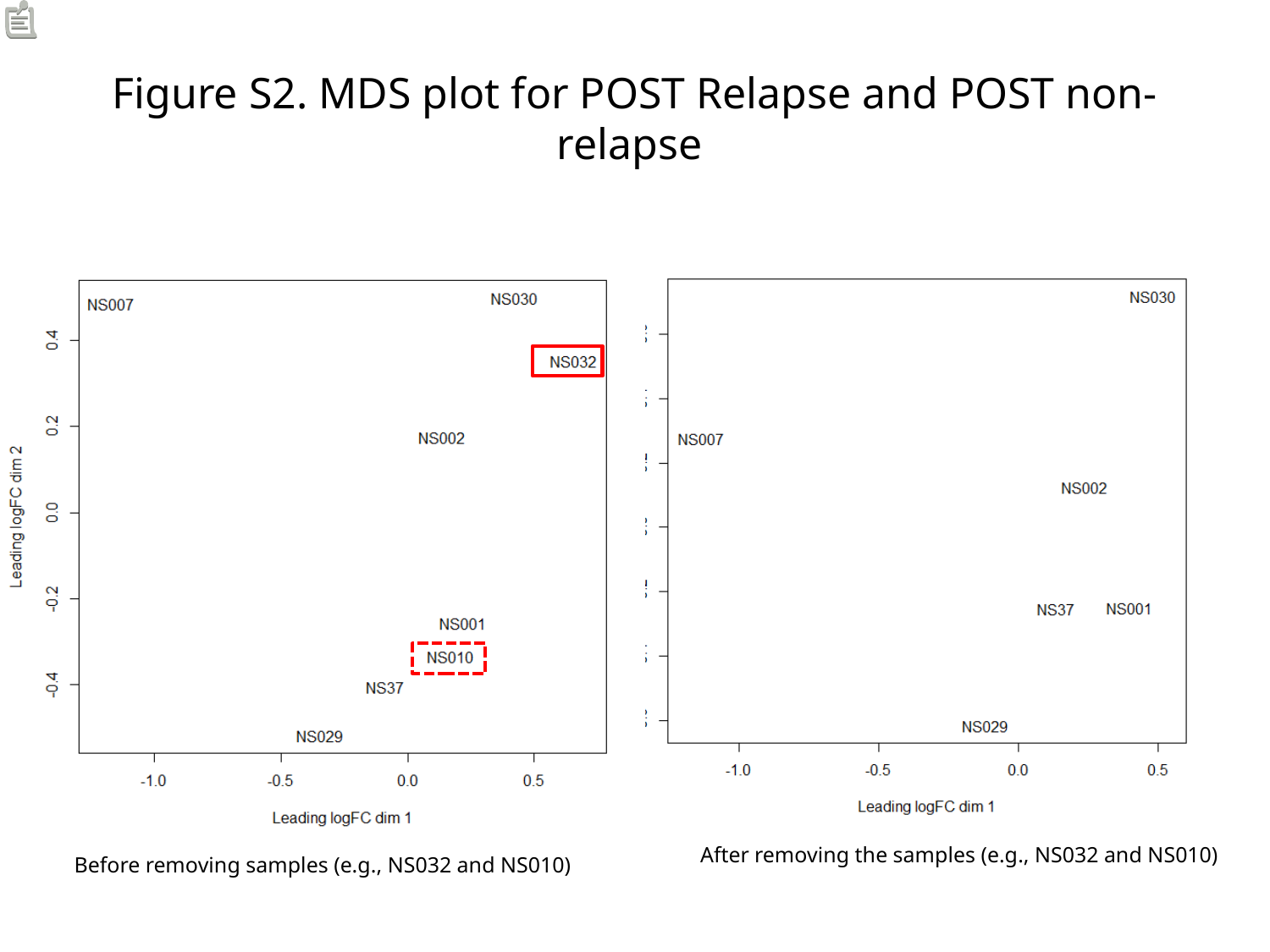

# Figure S2. MDS plot for POST Relapse and POST non-relapse
After removing the samples (e.g., NS032 and NS010)
Before removing samples (e.g., NS032 and NS010)

## Slide 3
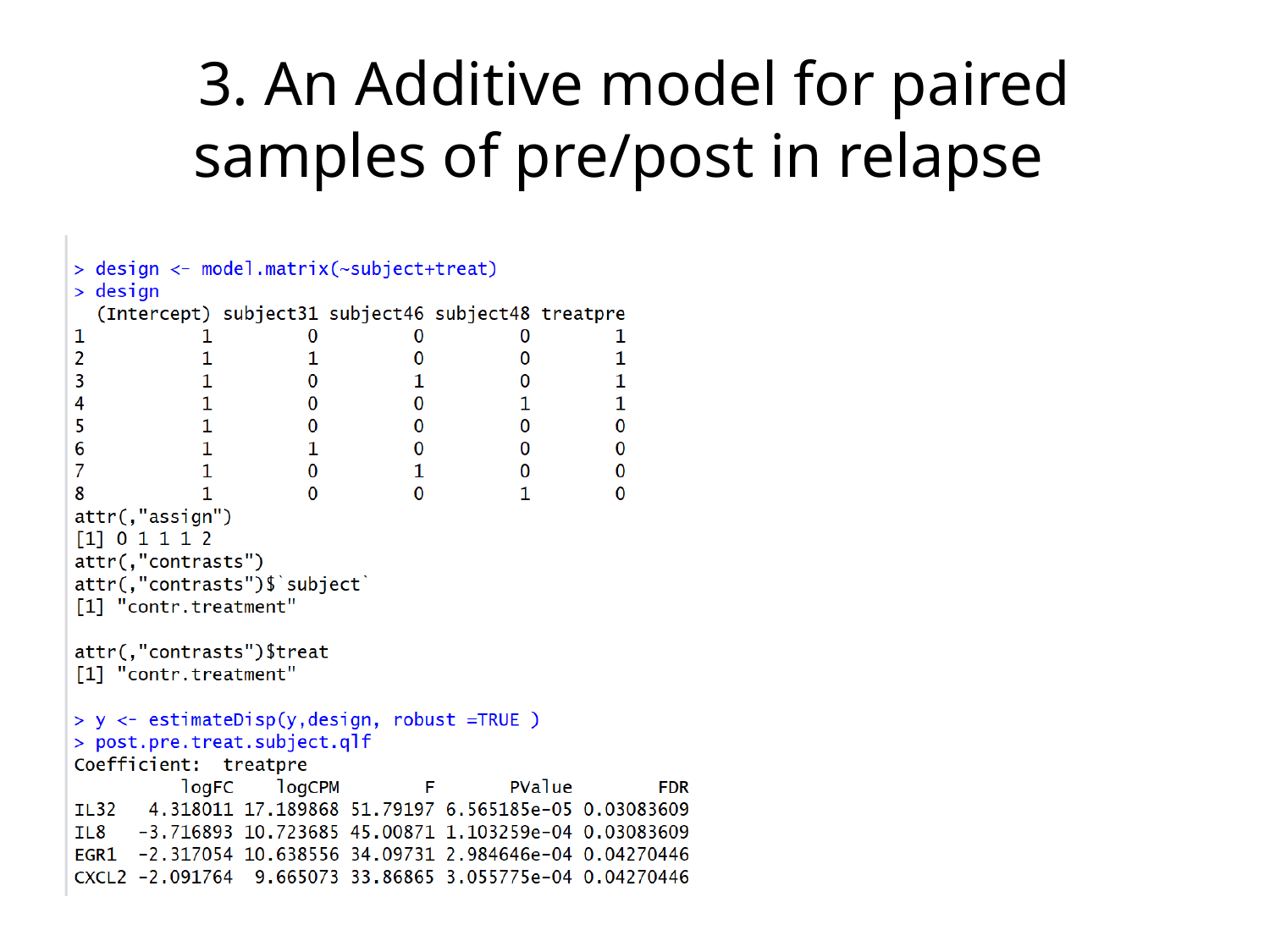

# 3. An Additive model for paired samples of pre/post in relapse

## Slide 4
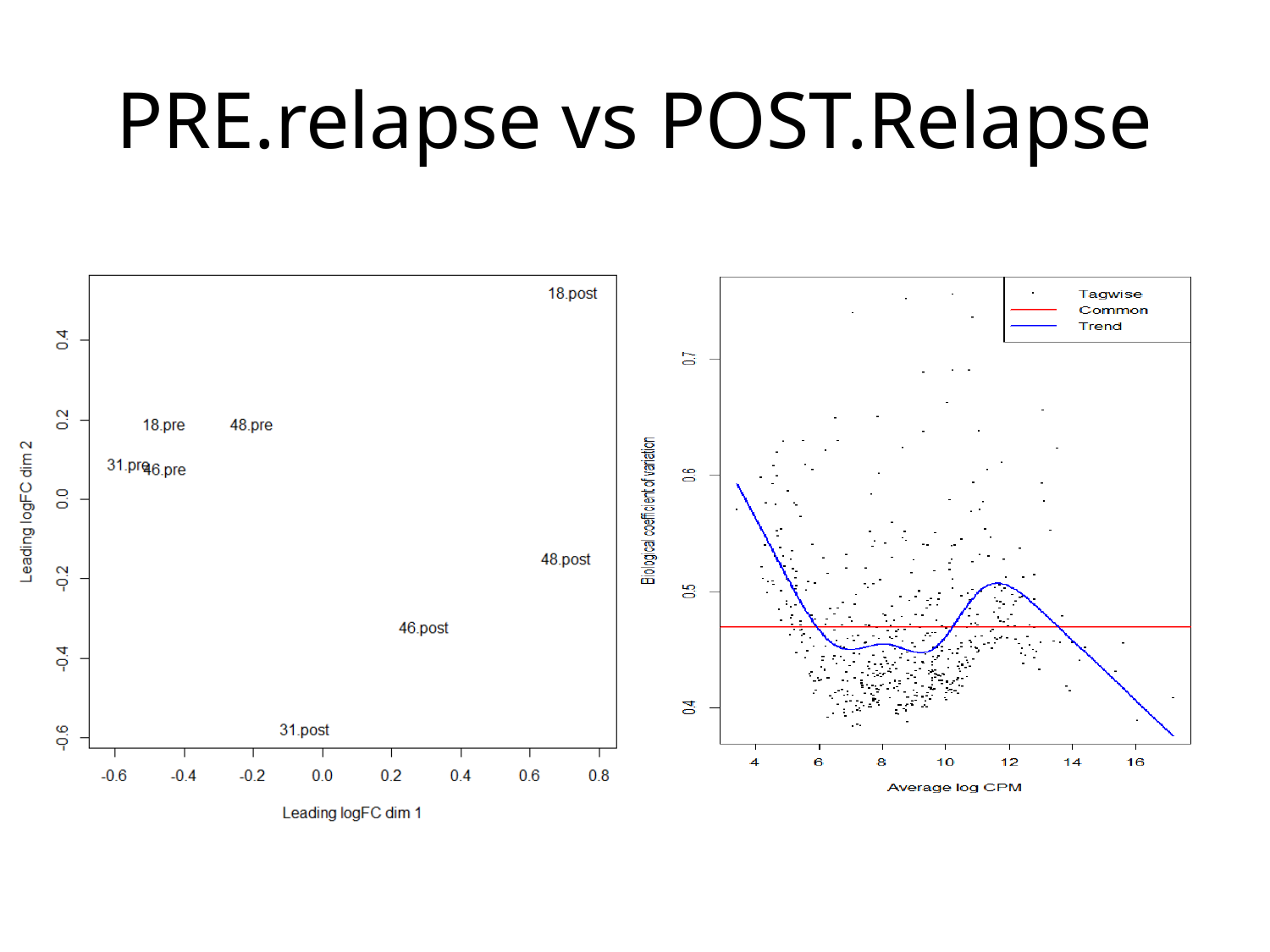

# PRE.relapse vs POST.Relapse

## Slide 5
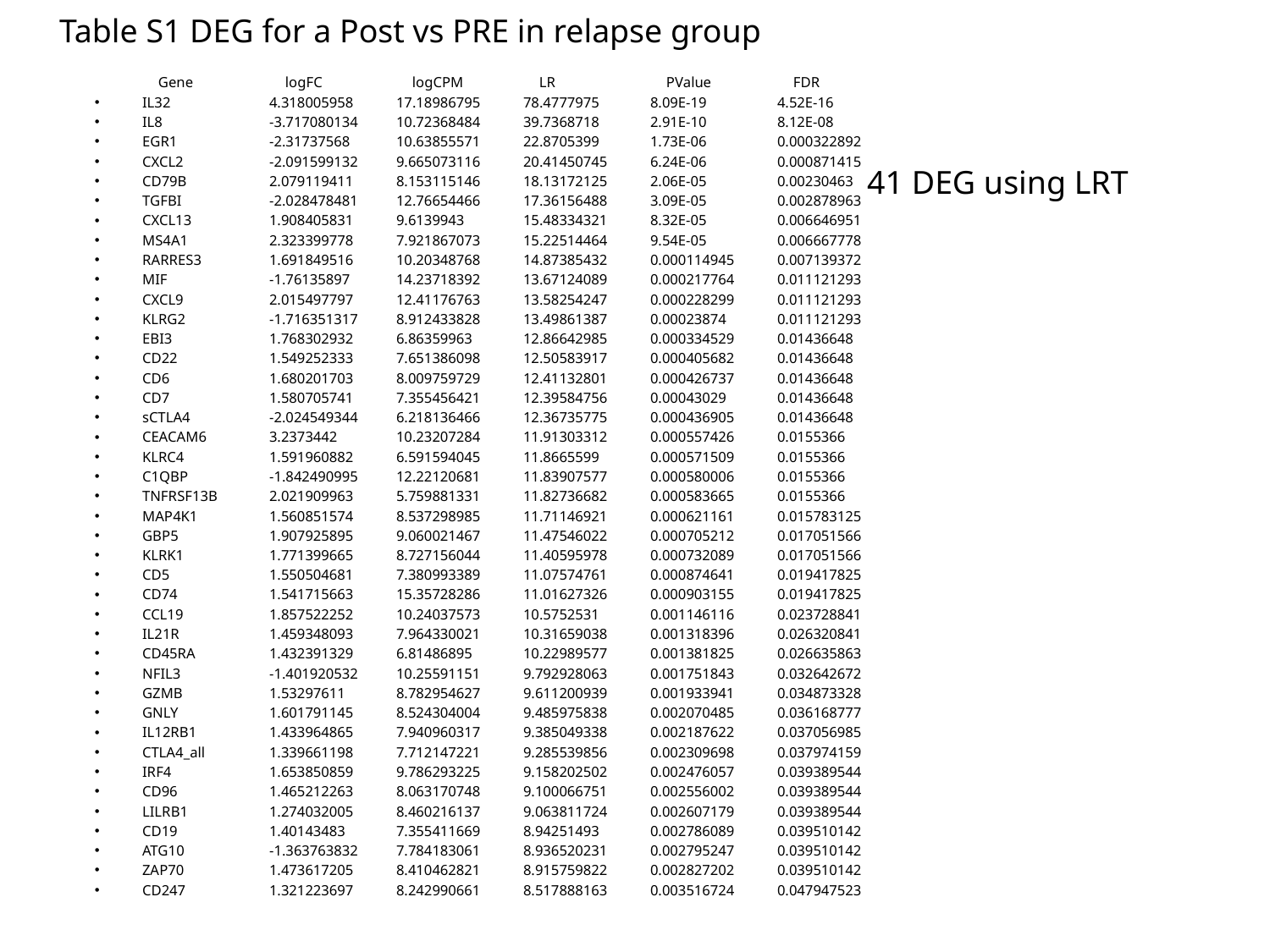

Table S1 DEG for a Post vs PRE in relapse group
Gene	logFC	logCPM	LR	PValue	FDR
IL32	4.318005958	17.18986795	78.4777975	8.09E-19	4.52E-16
IL8	-3.717080134	10.72368484	39.7368718	2.91E-10	8.12E-08
EGR1	-2.31737568	10.63855571	22.8705399	1.73E-06	0.000322892
CXCL2	-2.091599132	9.665073116	20.41450745	6.24E-06	0.000871415
CD79B	2.079119411	8.153115146	18.13172125	2.06E-05	0.00230463
TGFBI	-2.028478481	12.76654466	17.36156488	3.09E-05	0.002878963
CXCL13	1.908405831	9.6139943	15.48334321	8.32E-05	0.006646951
MS4A1	2.323399778	7.921867073	15.22514464	9.54E-05	0.006667778
RARRES3	1.691849516	10.20348768	14.87385432	0.000114945	0.007139372
MIF	-1.76135897	14.23718392	13.67124089	0.000217764	0.011121293
CXCL9	2.015497797	12.41176763	13.58254247	0.000228299	0.011121293
KLRG2	-1.716351317	8.912433828	13.49861387	0.00023874	0.011121293
EBI3	1.768302932	6.86359963	12.86642985	0.000334529	0.01436648
CD22	1.549252333	7.651386098	12.50583917	0.000405682	0.01436648
CD6	1.680201703	8.009759729	12.41132801	0.000426737	0.01436648
CD7	1.580705741	7.355456421	12.39584756	0.00043029	0.01436648
sCTLA4	-2.024549344	6.218136466	12.36735775	0.000436905	0.01436648
CEACAM6	3.2373442	10.23207284	11.91303312	0.000557426	0.0155366
KLRC4	1.591960882	6.591594045	11.8665599	0.000571509	0.0155366
C1QBP	-1.842490995	12.22120681	11.83907577	0.000580006	0.0155366
TNFRSF13B	2.021909963	5.759881331	11.82736682	0.000583665	0.0155366
MAP4K1	1.560851574	8.537298985	11.71146921	0.000621161	0.015783125
GBP5	1.907925895	9.060021467	11.47546022	0.000705212	0.017051566
KLRK1	1.771399665	8.727156044	11.40595978	0.000732089	0.017051566
CD5	1.550504681	7.380993389	11.07574761	0.000874641	0.019417825
CD74	1.541715663	15.35728286	11.01627326	0.000903155	0.019417825
CCL19	1.857522252	10.24037573	10.5752531	0.001146116	0.023728841
IL21R	1.459348093	7.964330021	10.31659038	0.001318396	0.026320841
CD45RA	1.432391329	6.81486895	10.22989577	0.001381825	0.026635863
NFIL3	-1.401920532	10.25591151	9.792928063	0.001751843	0.032642672
GZMB	1.53297611	8.782954627	9.611200939	0.001933941	0.034873328
GNLY	1.601791145	8.524304004	9.485975838	0.002070485	0.036168777
IL12RB1	1.433964865	7.940960317	9.385049338	0.002187622	0.037056985
CTLA4_all	1.339661198	7.712147221	9.285539856	0.002309698	0.037974159
IRF4	1.653850859	9.786293225	9.158202502	0.002476057	0.039389544
CD96	1.465212263	8.063170748	9.100066751	0.002556002	0.039389544
LILRB1	1.274032005	8.460216137	9.063811724	0.002607179	0.039389544
CD19	1.40143483	7.355411669	8.94251493	0.002786089	0.039510142
ATG10	-1.363763832	7.784183061	8.936520231	0.002795247	0.039510142
ZAP70	1.473617205	8.410462821	8.915759822	0.002827202	0.039510142
CD247	1.321223697	8.242990661	8.517888163	0.003516724	0.047947523
41 DEG using LRT

## Slide 6
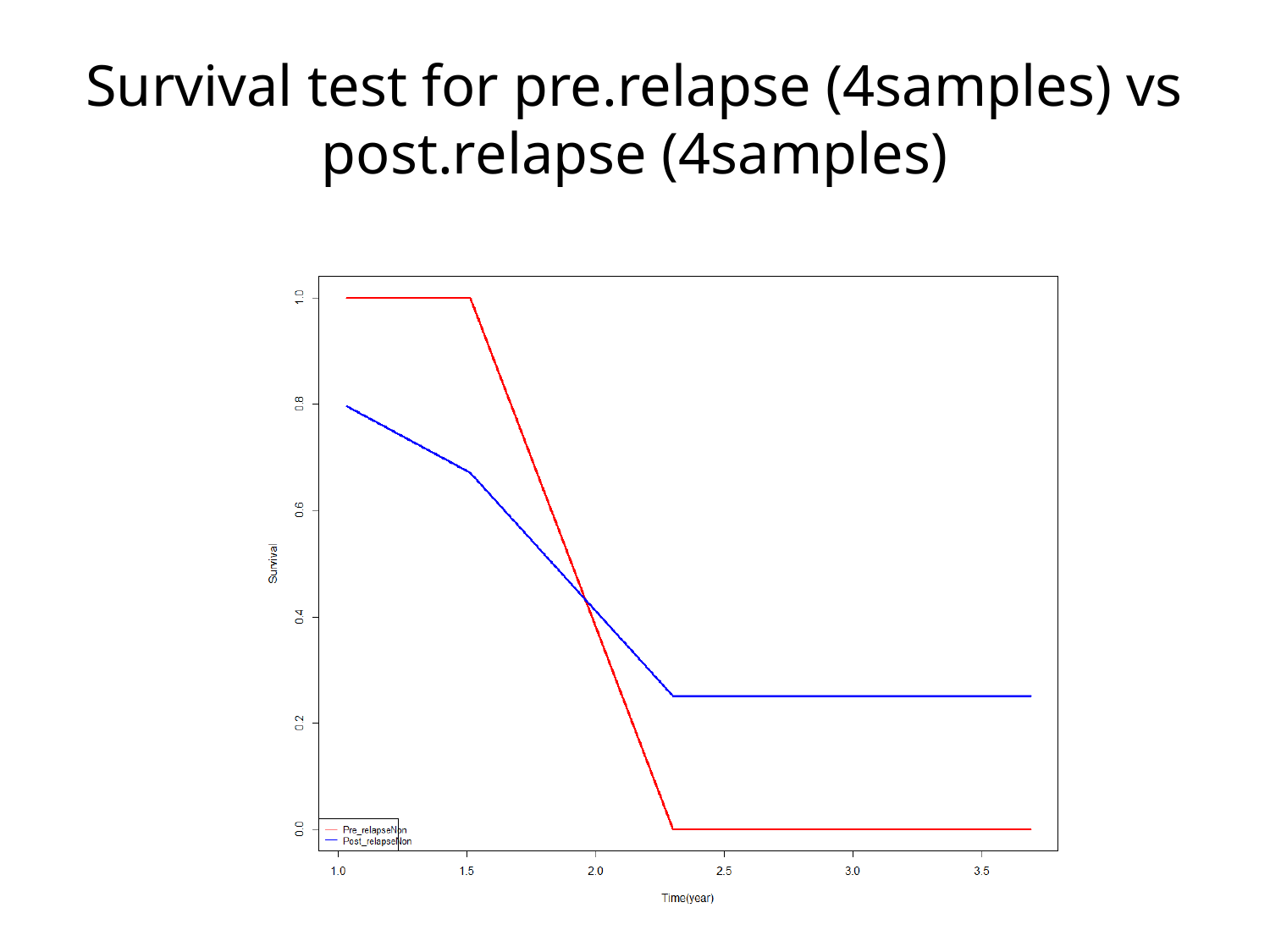

# Survival test for pre.relapse (4samples) vs post.relapse (4samples)
