# Supplementary material for: Prediction of prognostic signatures in triple-negative breast cancer based on the differential expression analysis via NanoString nCounter immune panel
Source: BMC Cancer. 2020 Nov 2;20:1052. doi: 10.1186/s12885-020-07399-8 (PMC7607642; doi:10.1186/s12885-020-07399-8)
Supplement: Supplementary file 2 — Additional file 2. 41 DEGs for comparison of POST to PRE in relapse. Figure S1. Violin plots for 41 DEGs for comparison of POST versus PRE in relapse. [file 12885_2020_7399_MOESM2_ESM.pdf]

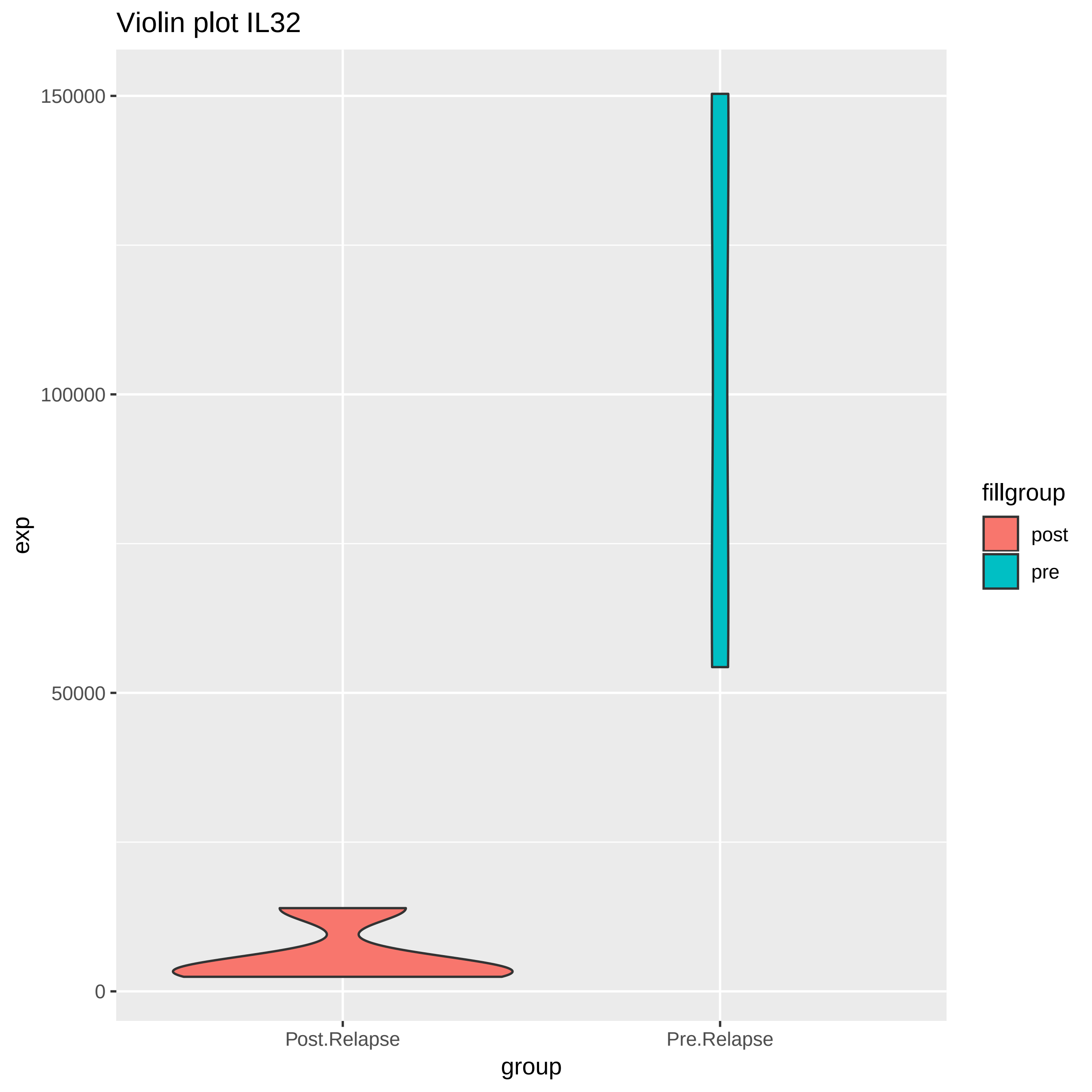

Violin plot IL32
150000
100000
50000
0
exp
Post.Relapse
Pre.Relapse
group
fillgroup
post
pre

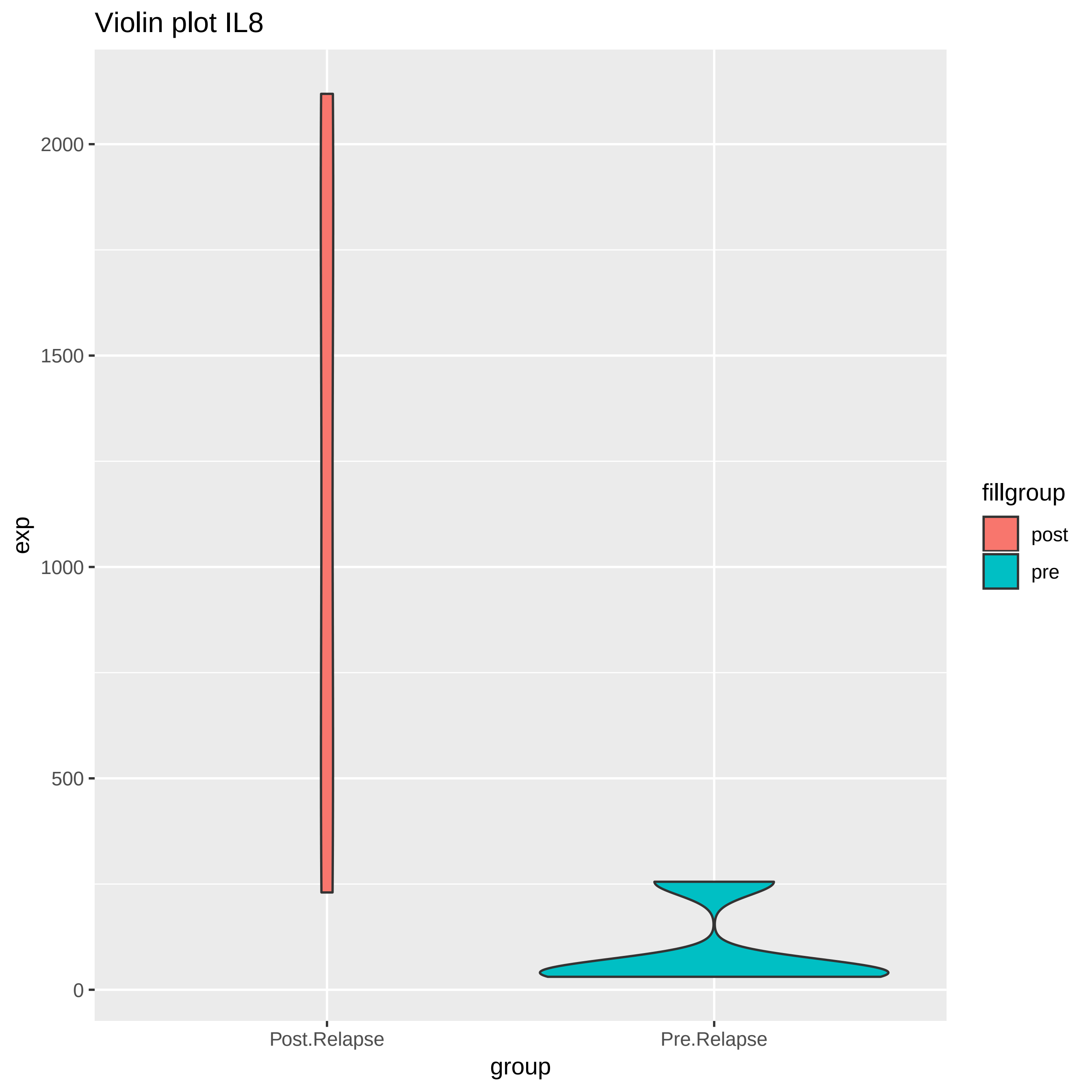
Violin plot IL8
exp
2000
1500
1000
500
0
Post.Relapse
Pre.Relapse
group
fillgroup
post
pre

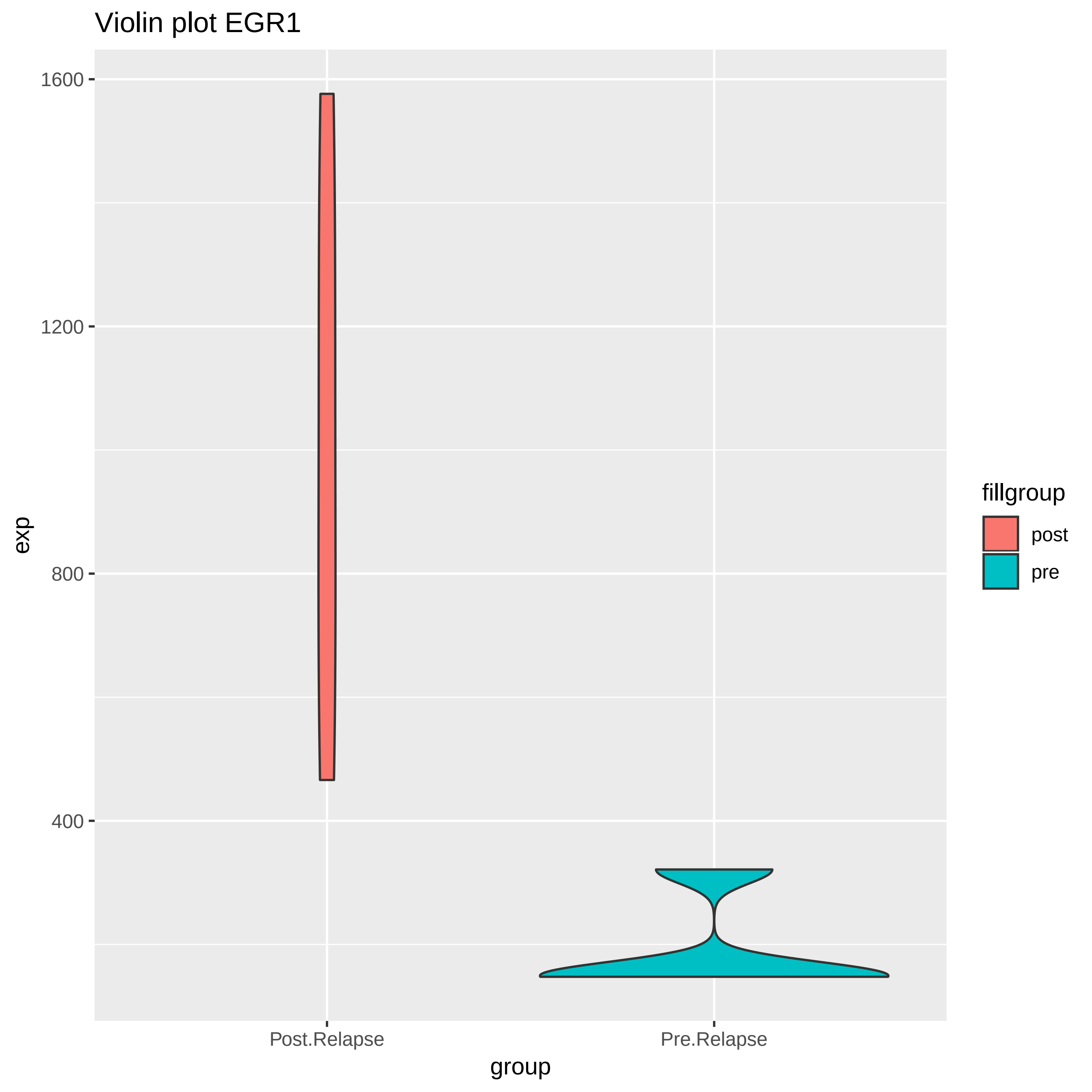

Violin plot EGR1
1600
1200
800
400
exp
Post.Relapse
Pre.Relapse
group
fillgroup
post
pre

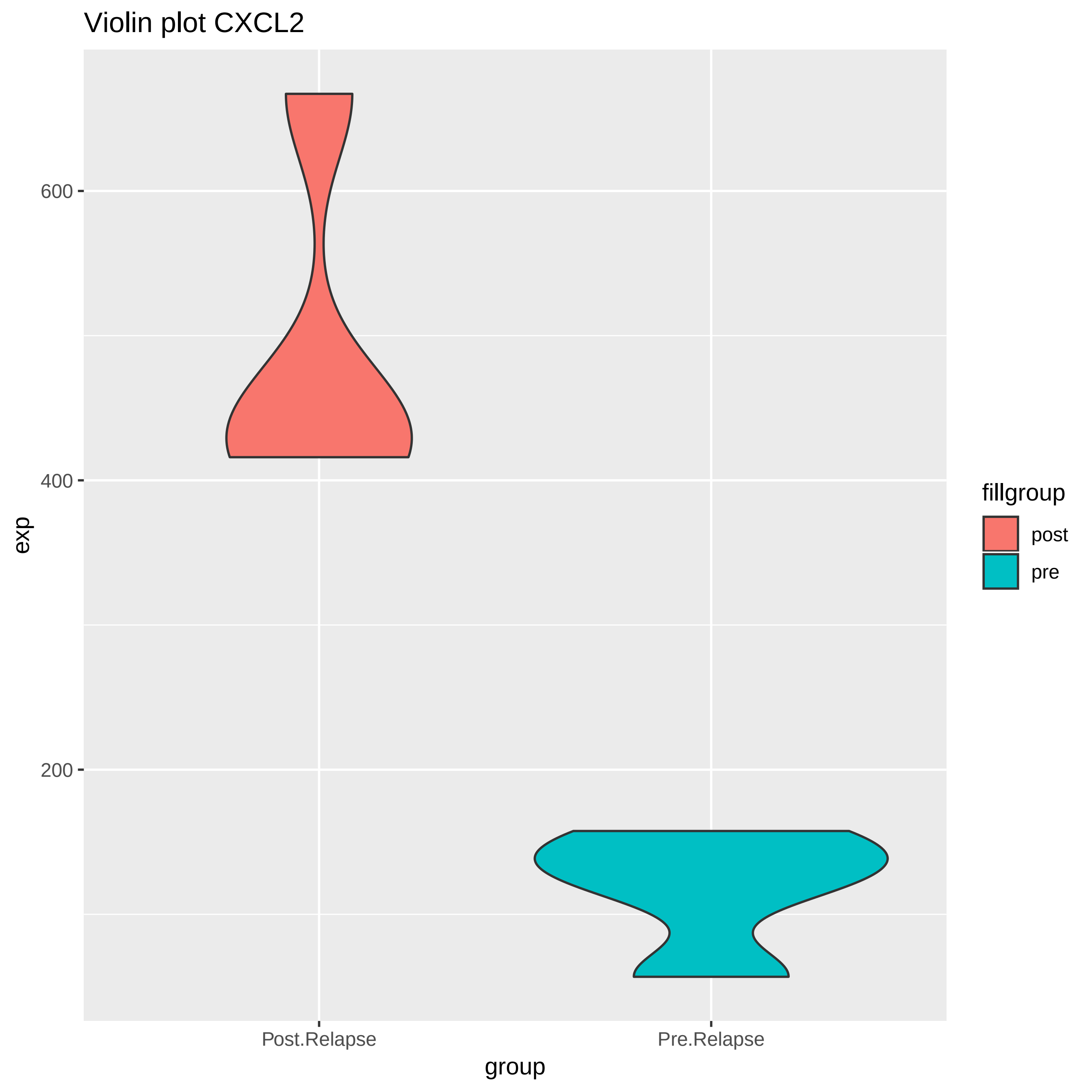

Violin plot CXCL2
600
400
200
exp
Post.Relapse
Pre.Relapse
group
fillgroup
post
pre

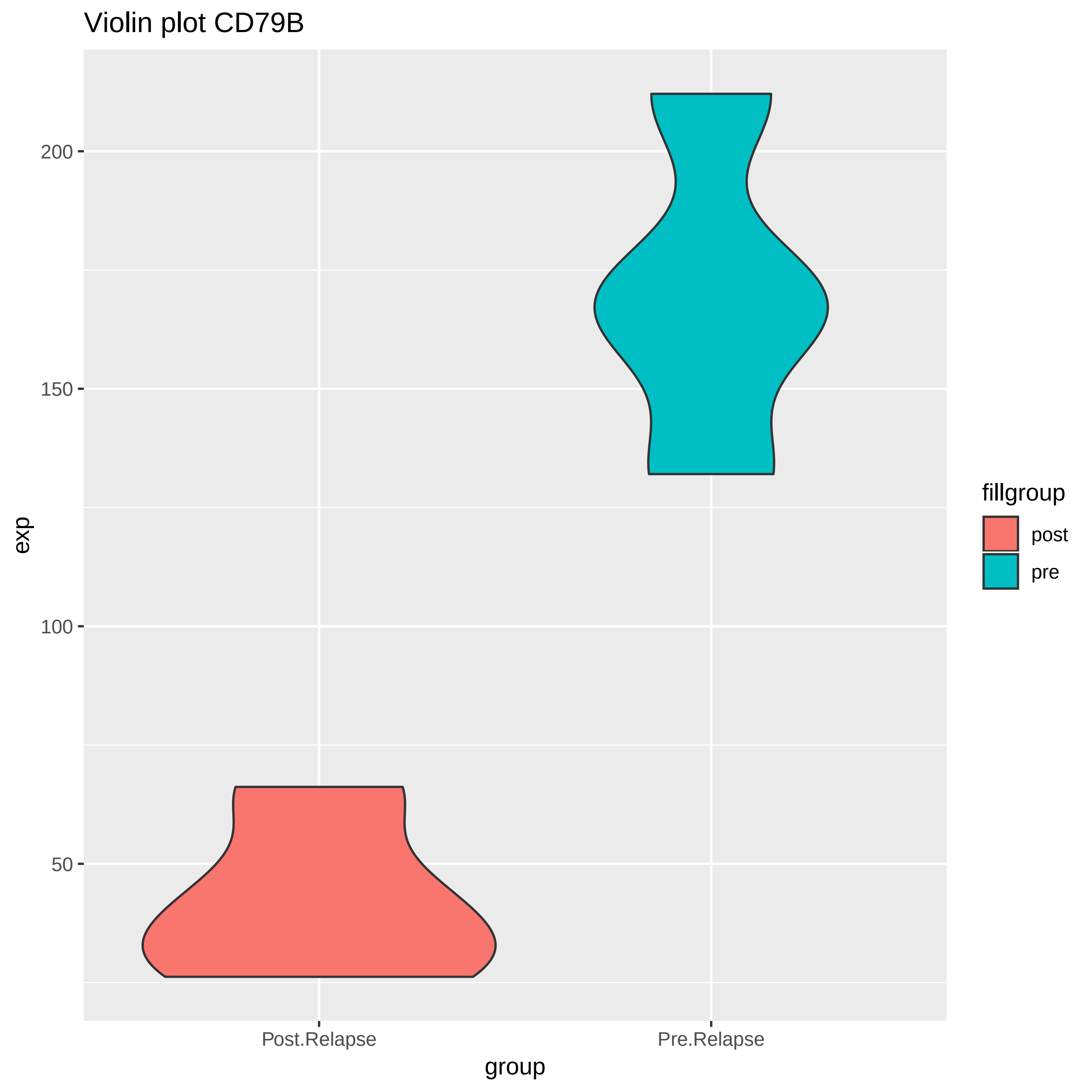

Violin plot CD79B
200
150
100
50
exp
Post.Relapse
Pre.Relapse
group
fillgroup
post
pre

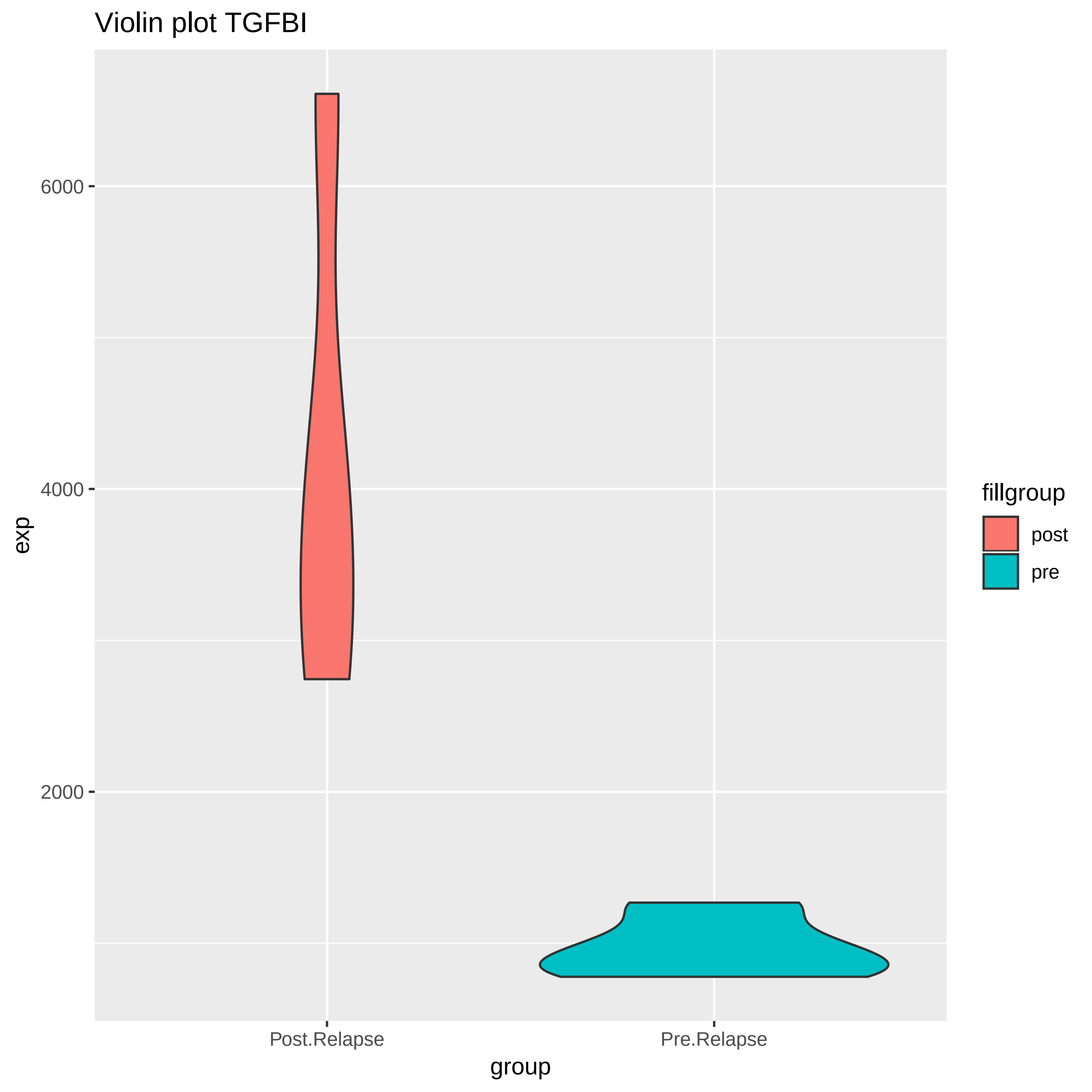

Violin plot TGFBI
6000
4000
2000
exp
Post.Relapse
Pre.Relapse
group
fillgroup
post
pre

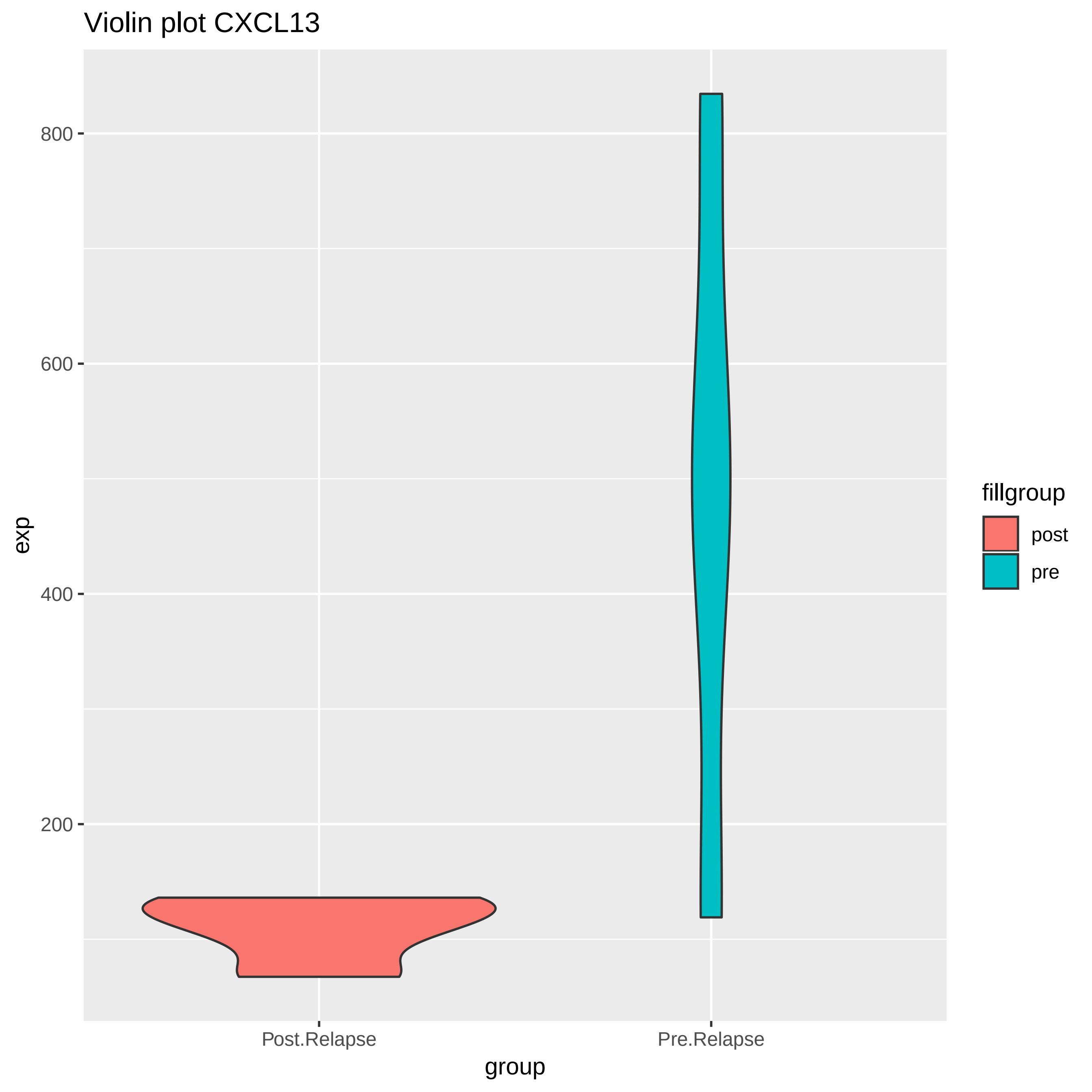
Violin plot CXCL13
800
600
400
200
exp
Post.Relapse
Pre.Relapse
group
fillgroup
post
pre

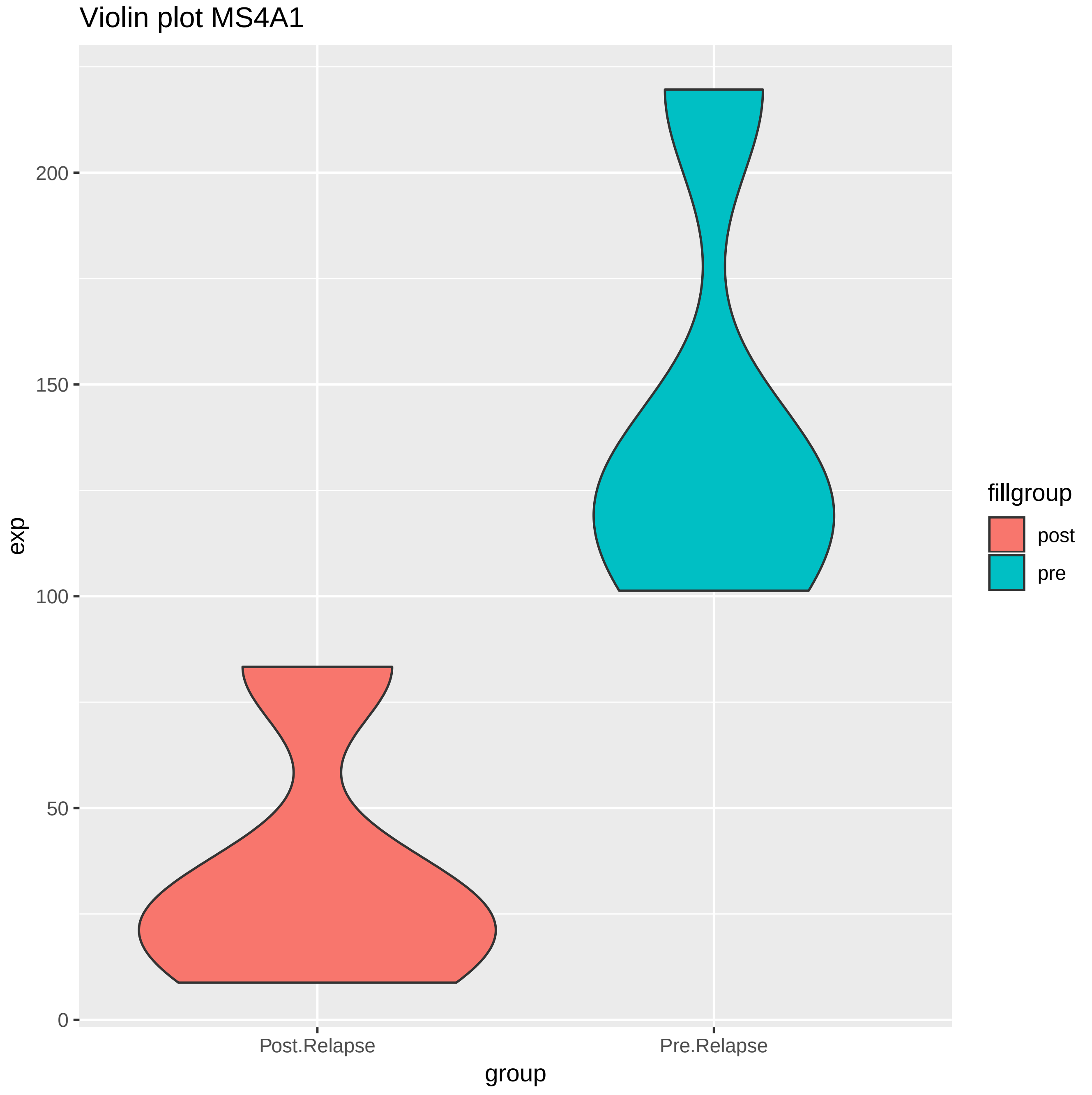

Violin plot MS4A1
200
150
100
50
0
exp
Post.Relapse
Pre.Relapse
group
fillgroup
post
pre

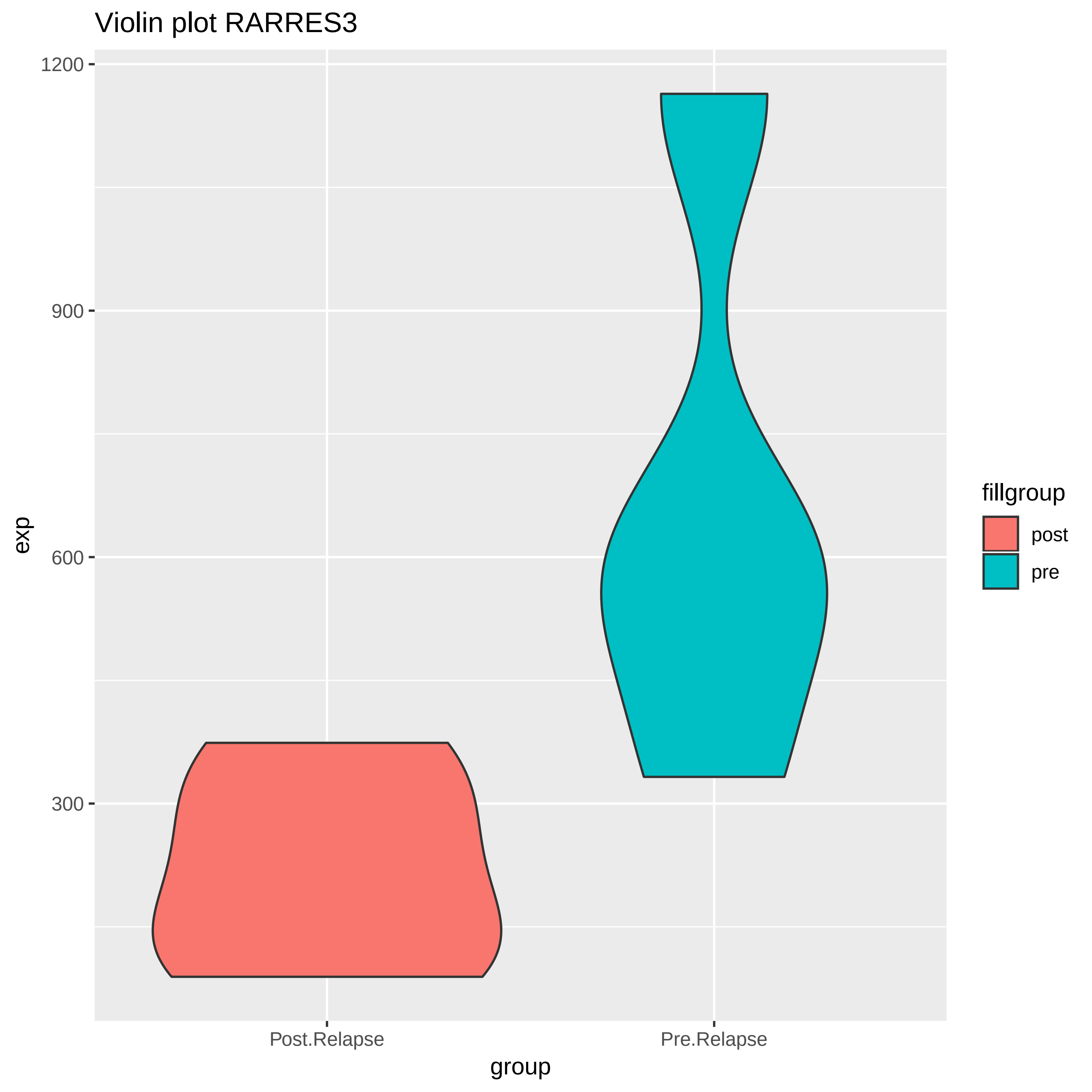
Violin plot RARRES3
1200
900
600
300
exp
Post.Relapse
Pre.Relapse
group
fillgroup
post
pre

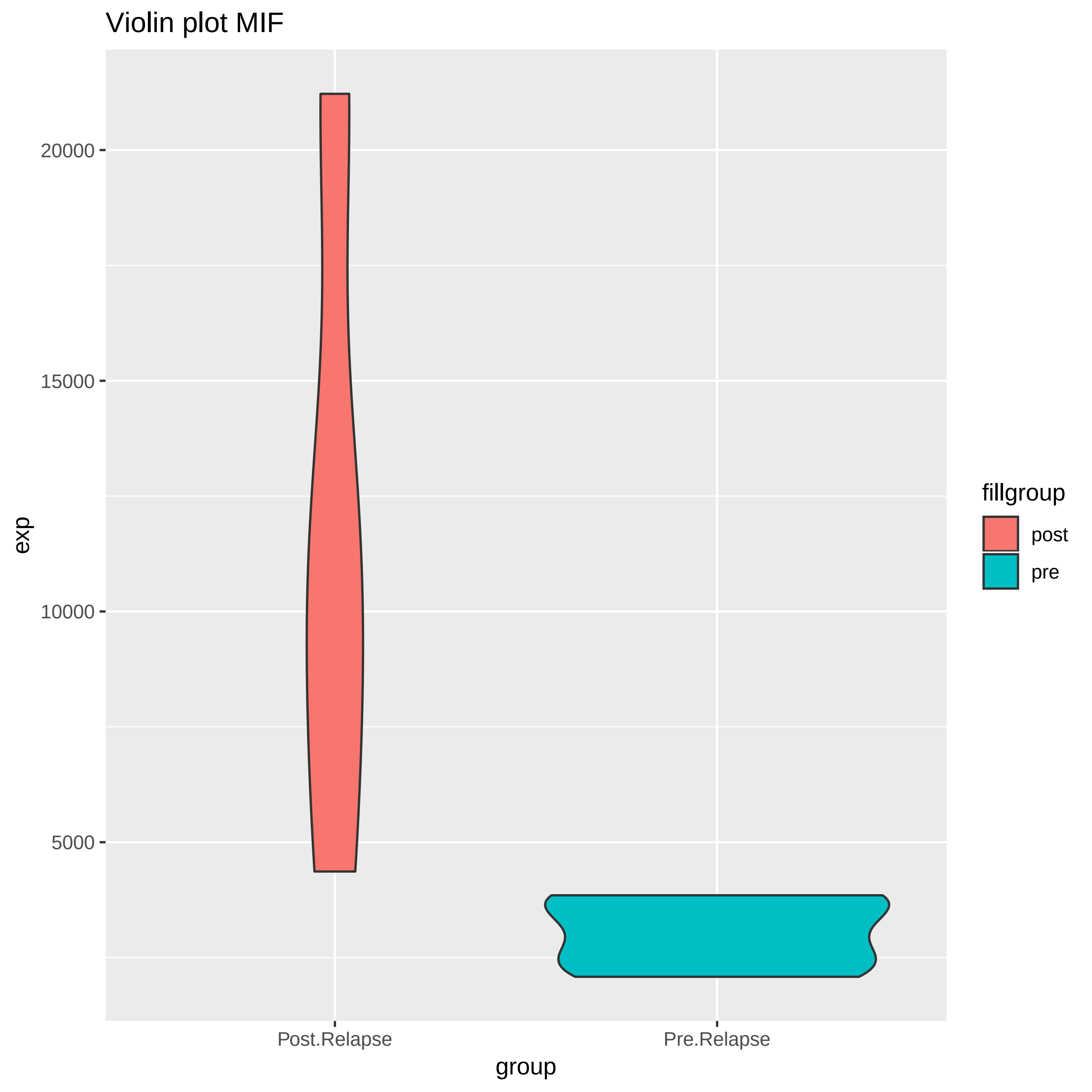
Violin plot MIF
20000
15000
10000
5000
exp
Post.Relapse
Pre.Relapse
group
fillgroup
post
pre

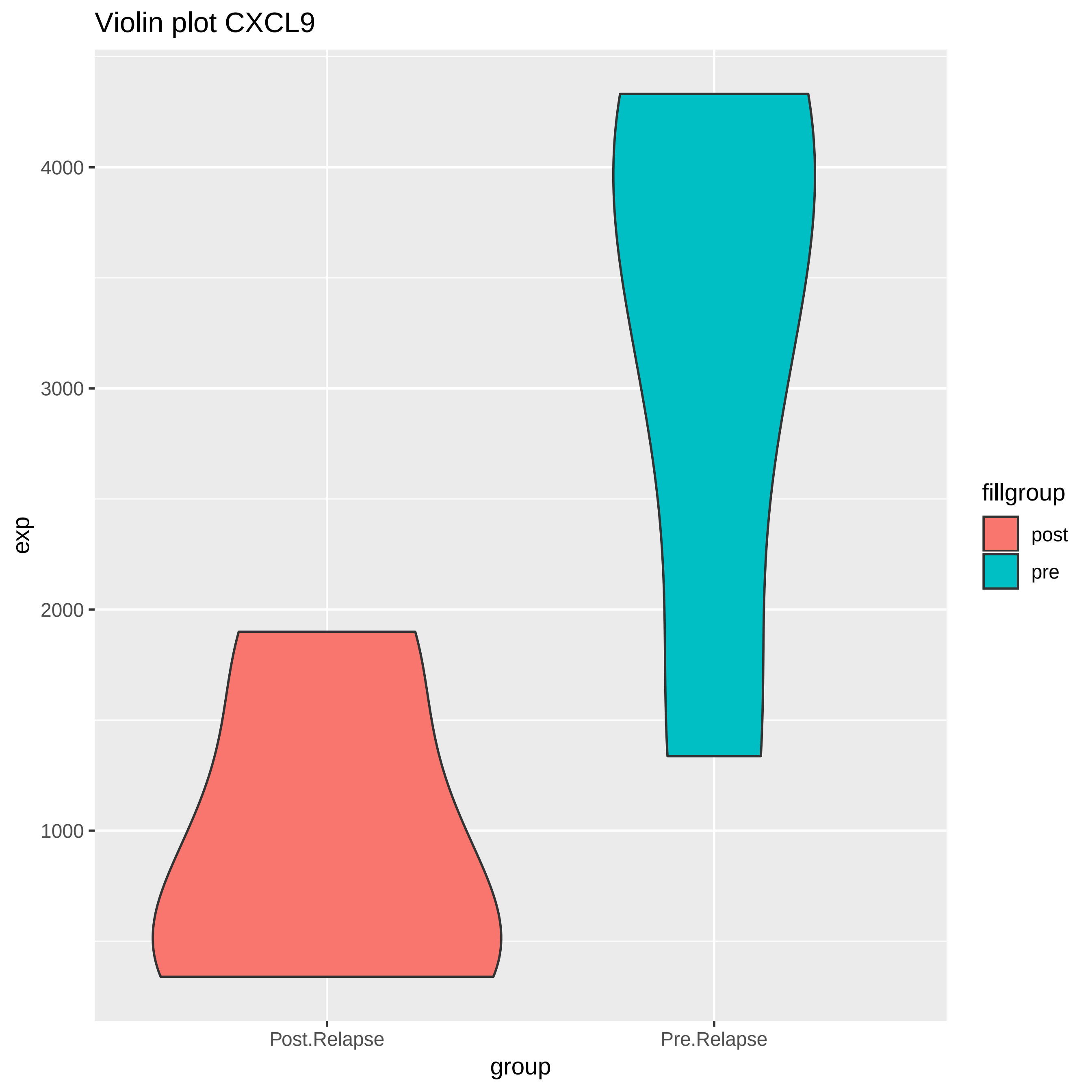

Violin plot CXCL9
exp
4000
3000
2000
1000
group
Post.Relapse
Pre.Relapse
fillgroup
post
pre

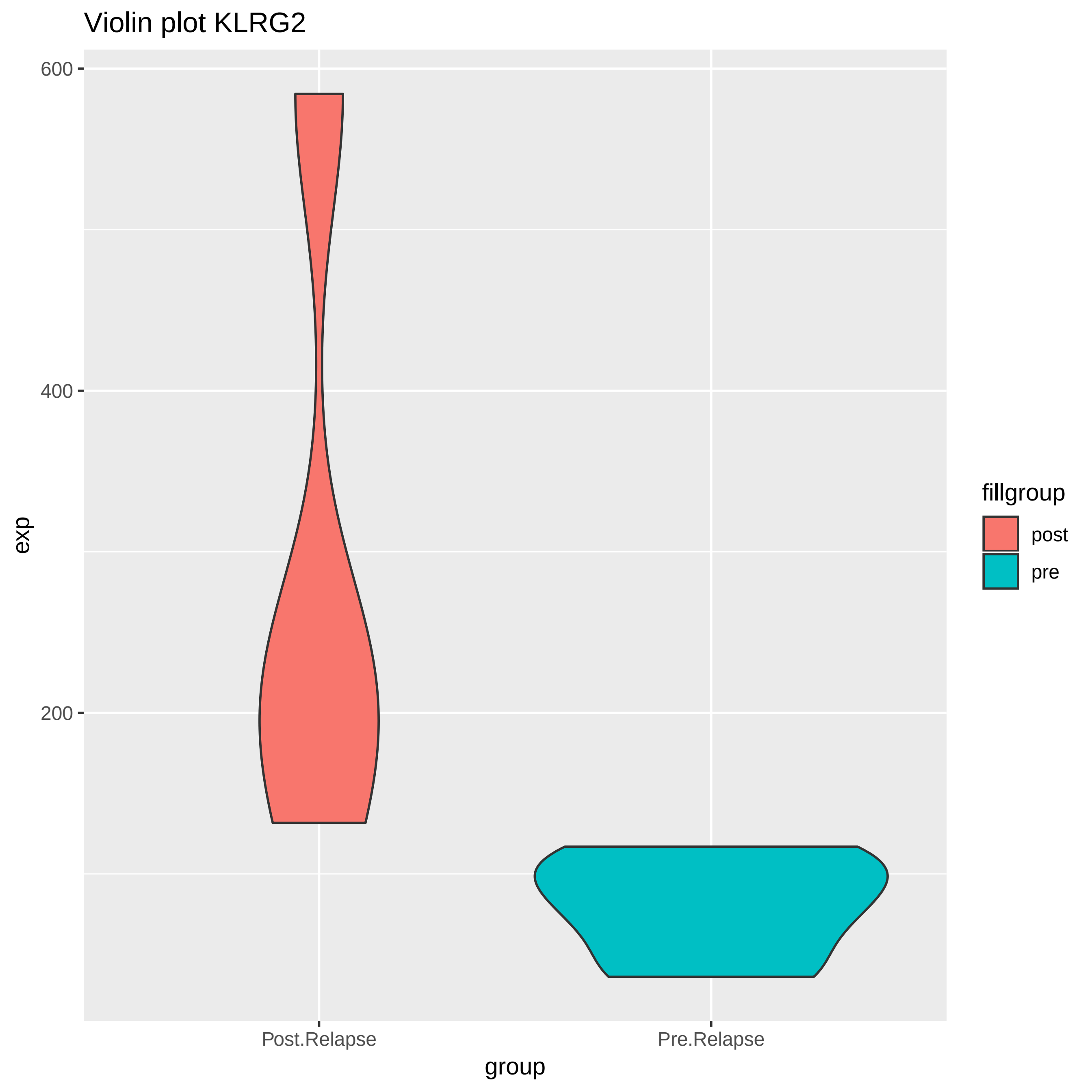

Violin plot KLRG2
600
400
200
exp
Post.Relapse
Pre.Relapse
group
fillgroup
post
pre

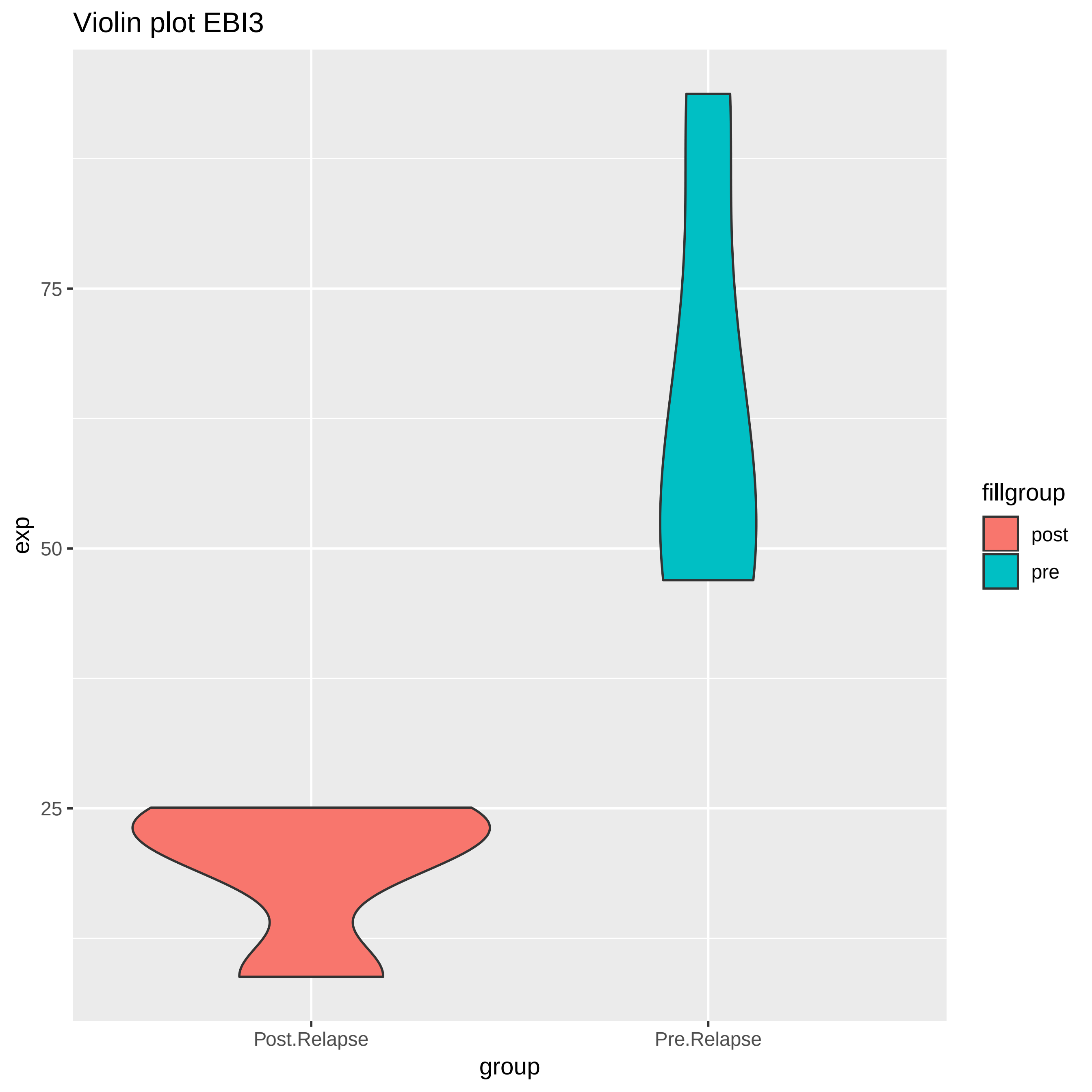

Violin plot EBI3
exp
75
50
25
group
Post.Relapse
Pre.Relapse
fillgroup
post
pre

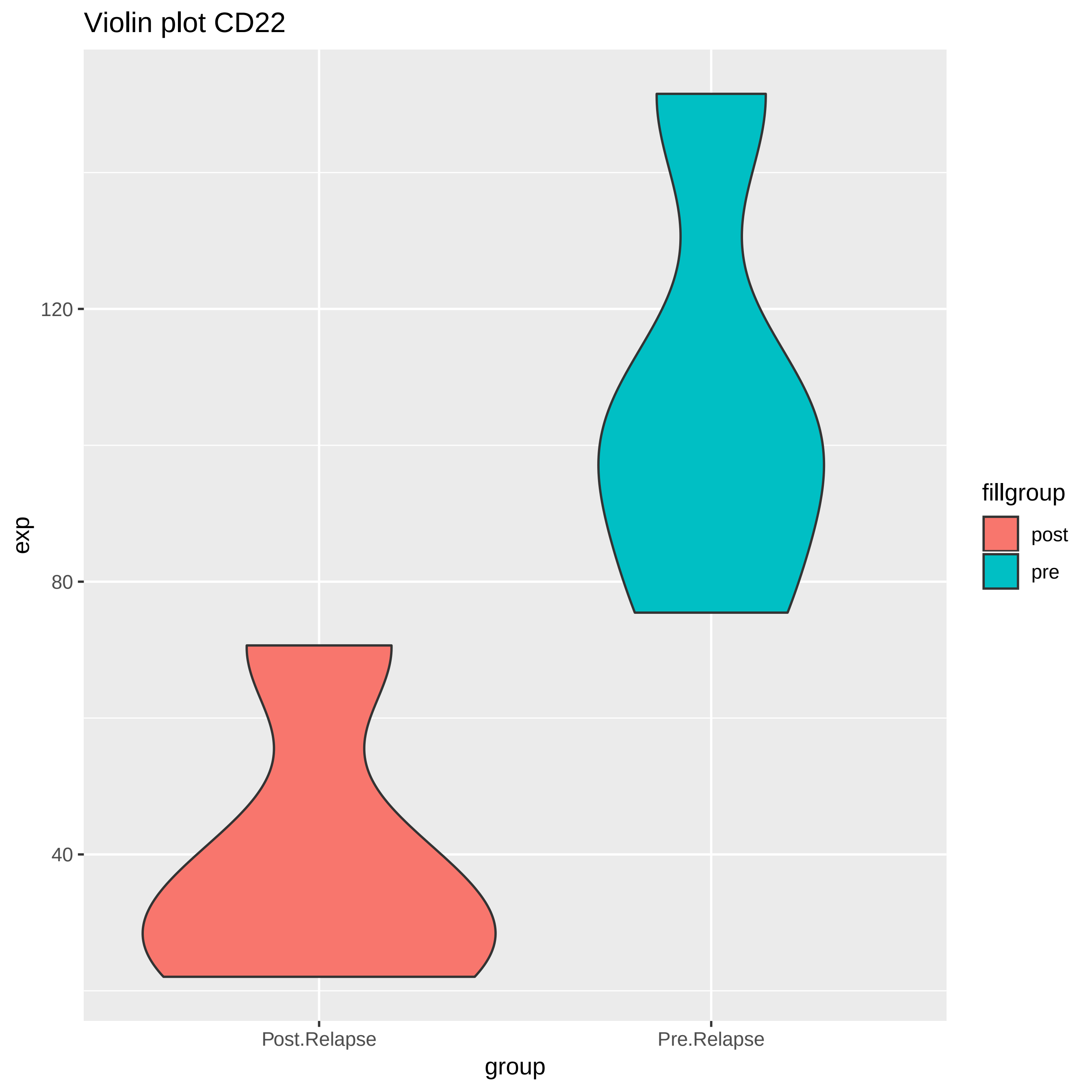

Violin plot CD22
exp
120
80
40
Post.Relapse
Pre.Relapse
group
fillgroup
post
pre

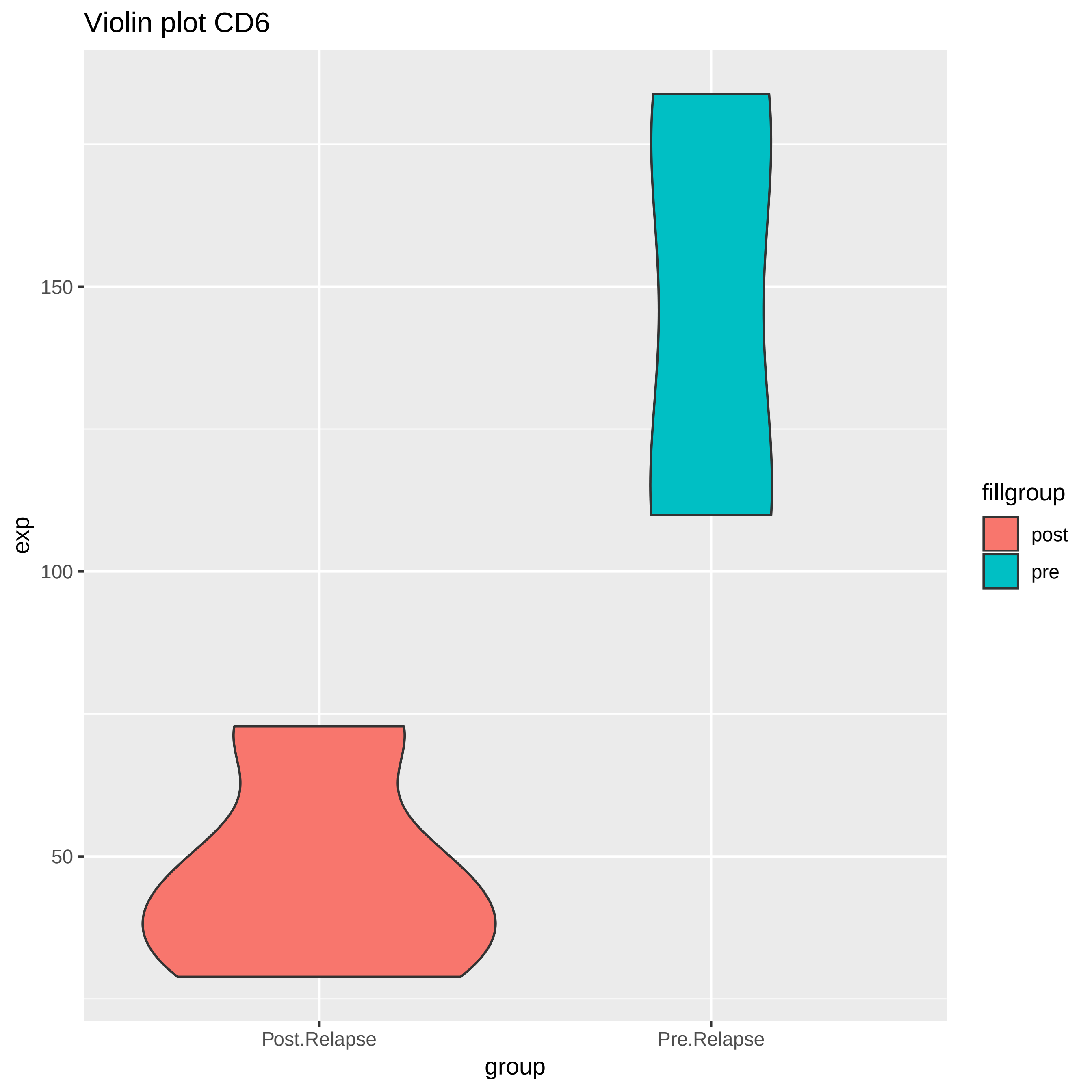

Violin plot CD6
exp
150
100
50
Post.Relapse
Pre.Relapse
group
fillgroup
post
pre

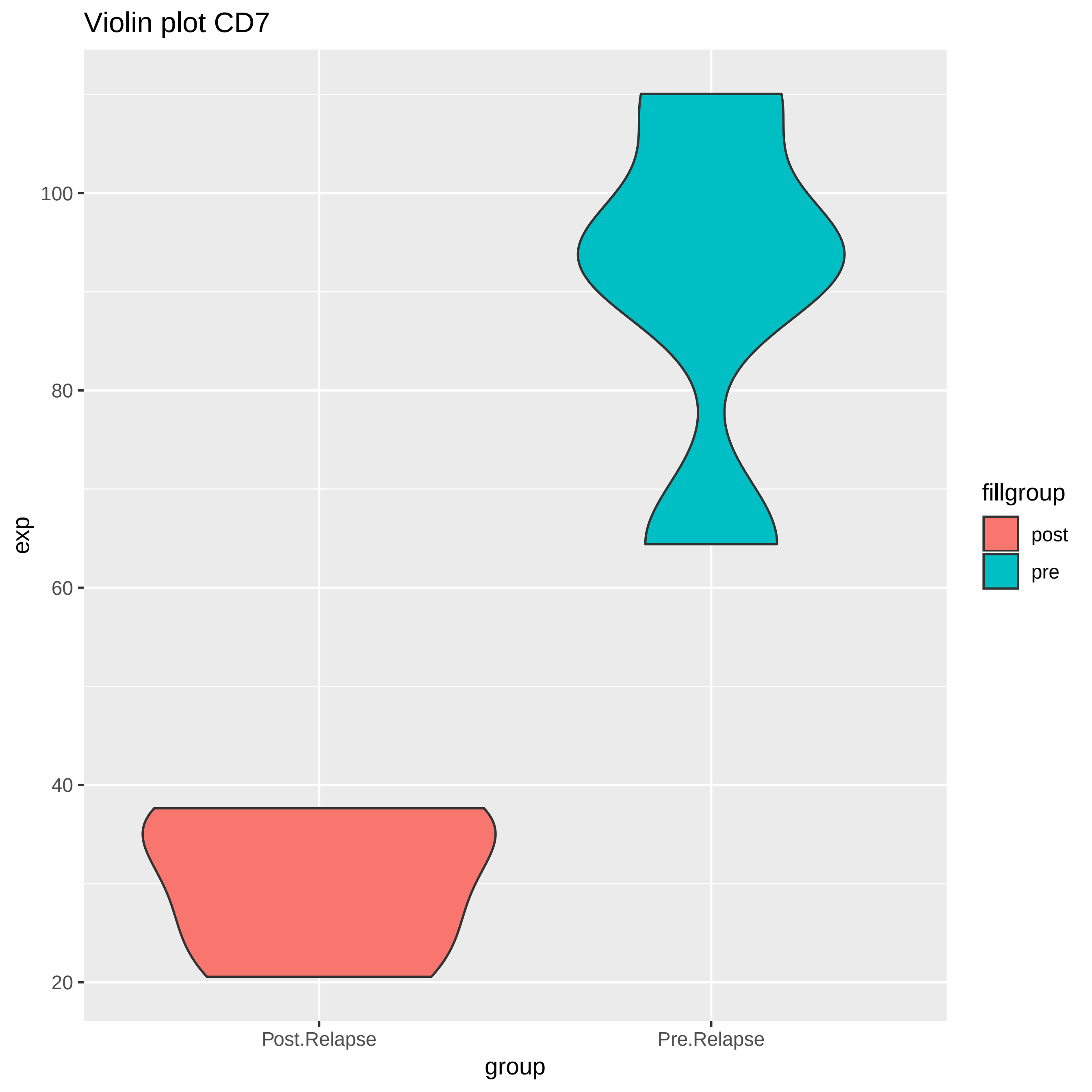

Violin plot CD7
100
80
60
40
20
exp
Post.Relapse
Pre.Relapse
group
fillgroup
post
pre

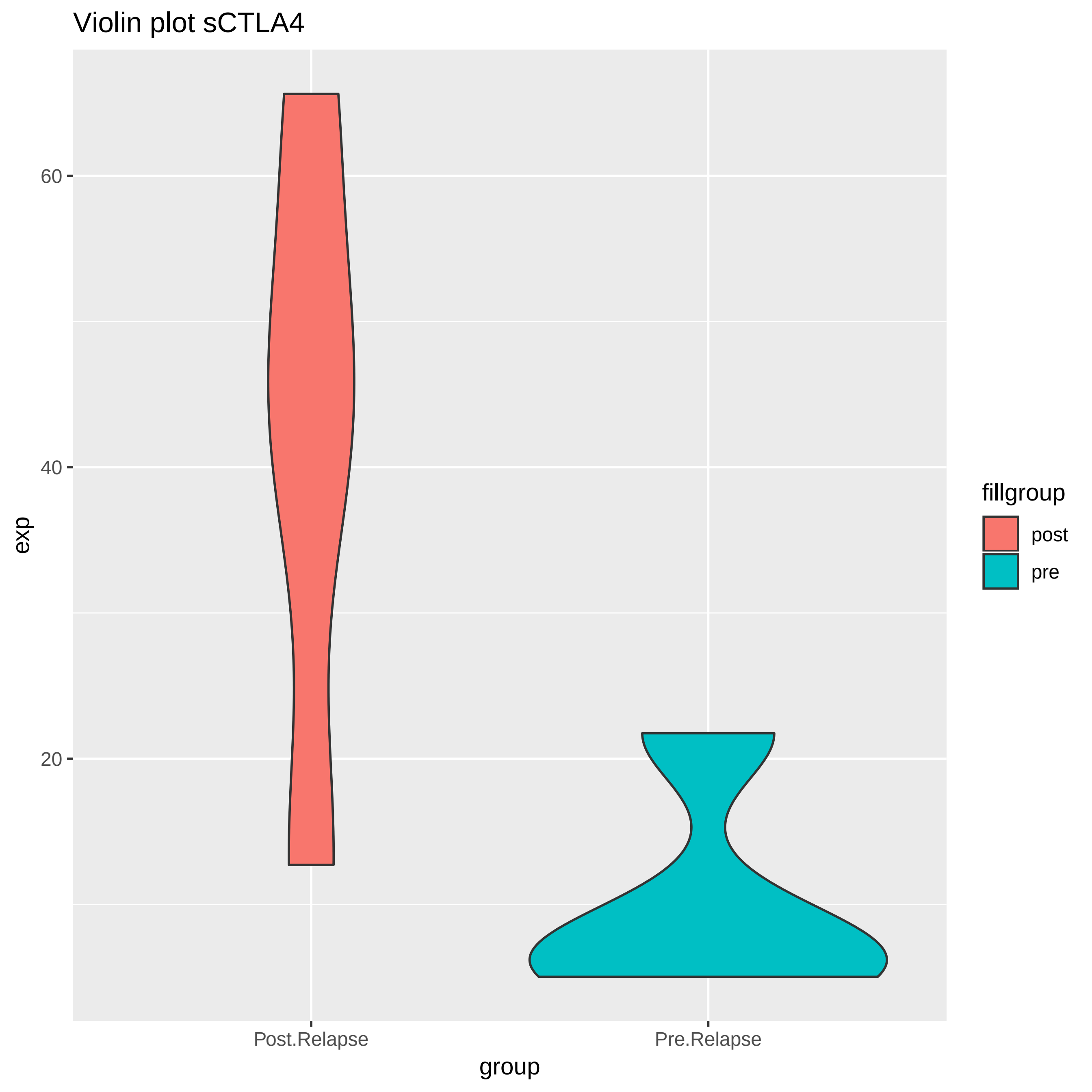

Violin plot sCTLA4
60
40
20
exp
Post.Relapse
Pre.Relapse
group
fillgroup
post
pre

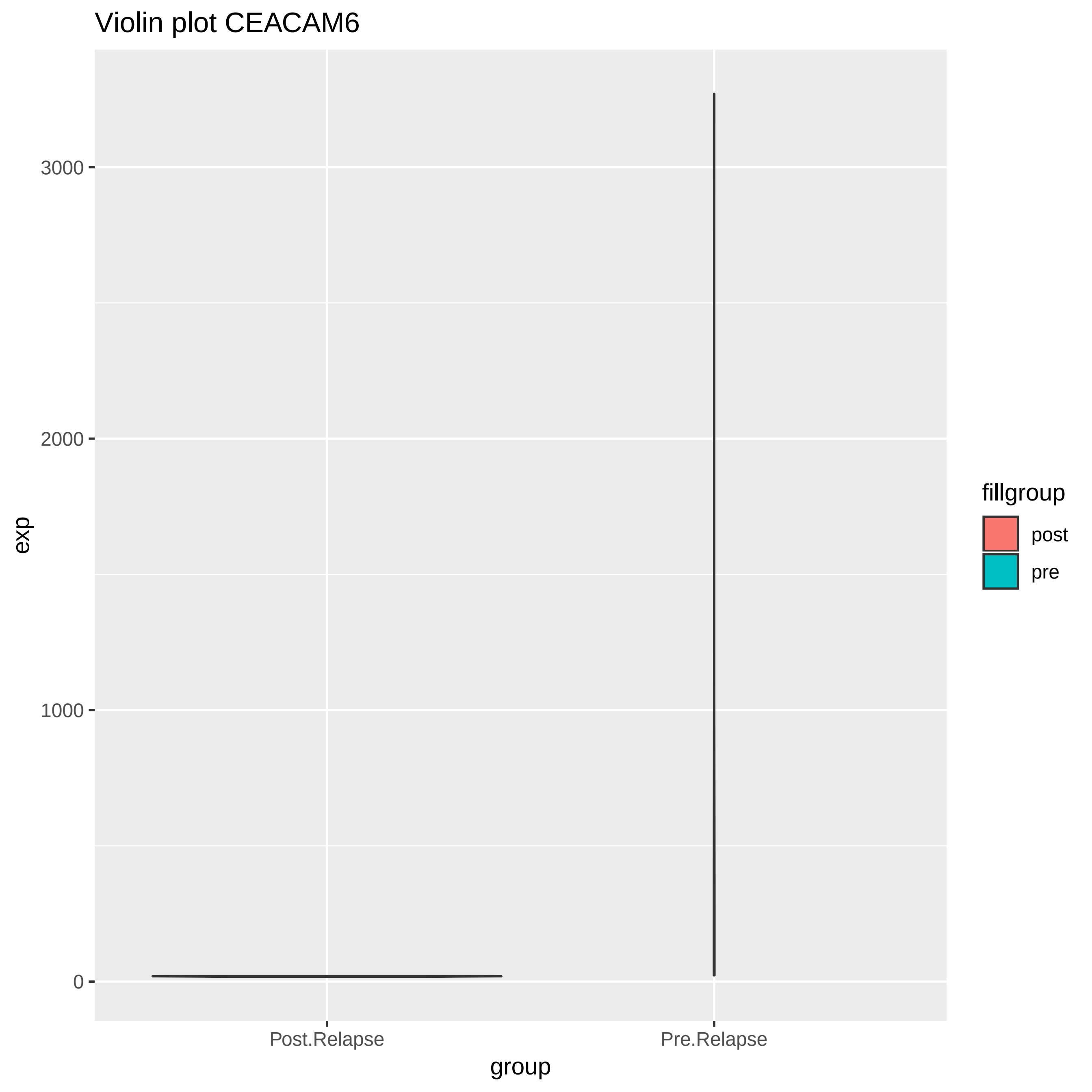
Violin plot CEACAM6
3000
2000
1000
0
exp
Post.Relapse
Pre.Relapse
group
fillgroup
post
pre

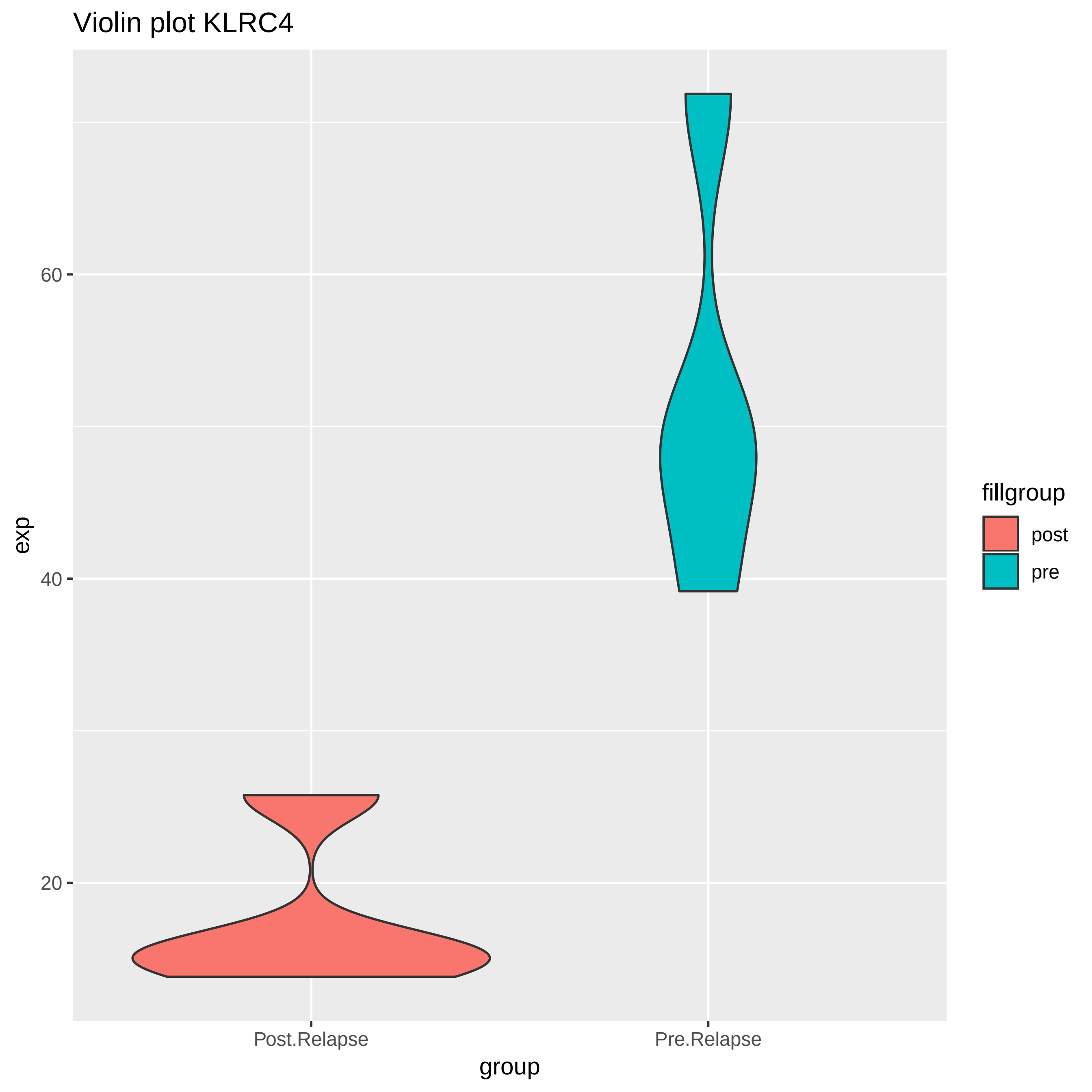
Violin plot KLRC4
60
40
20
exp
Post.Relapse
Pre.Relapse
group
fillgroup
post
pre

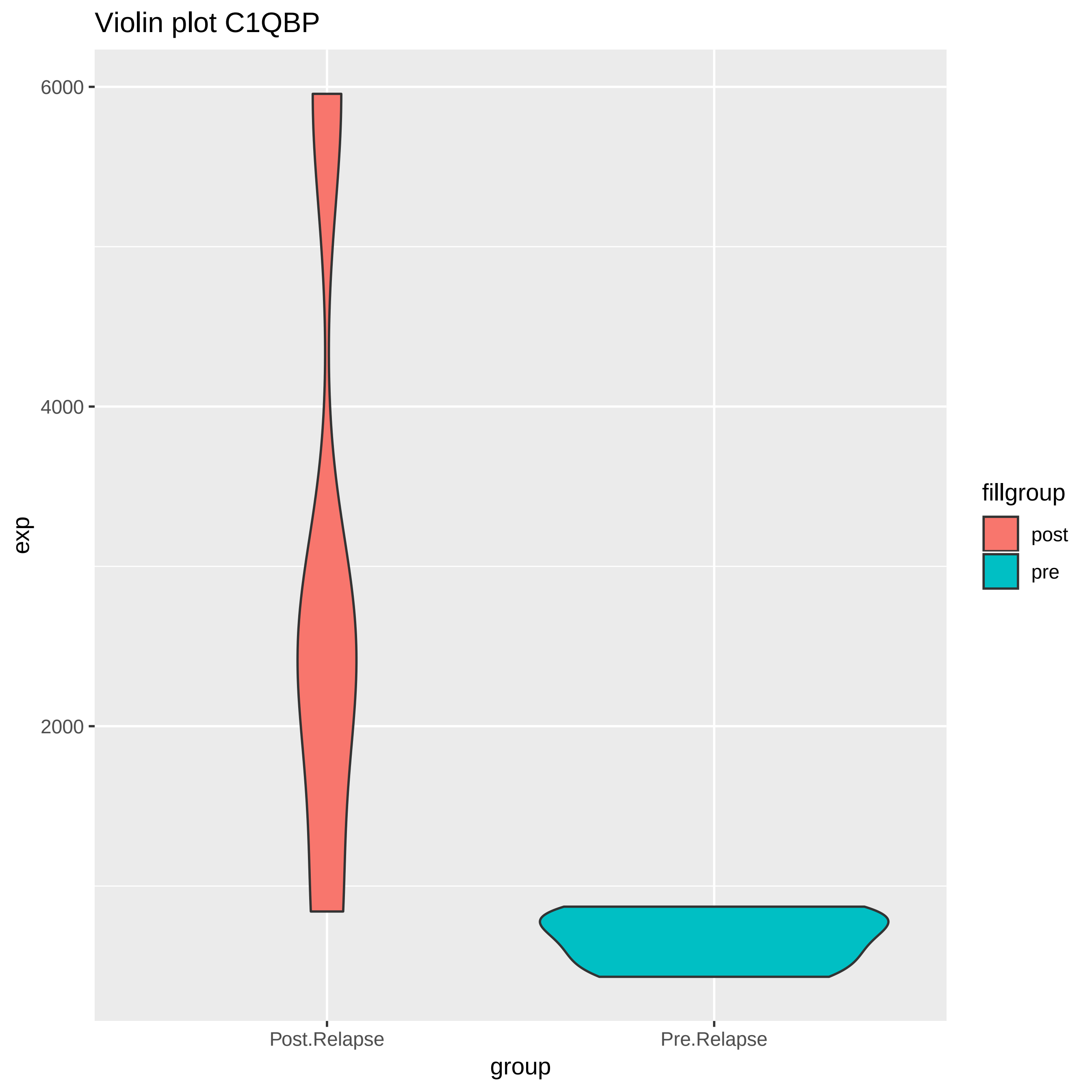

Violin plot C1QBP
6000
4000
2000
exp
group
Post.Relapse
Pre.Relapse
fillgroup
post
pre

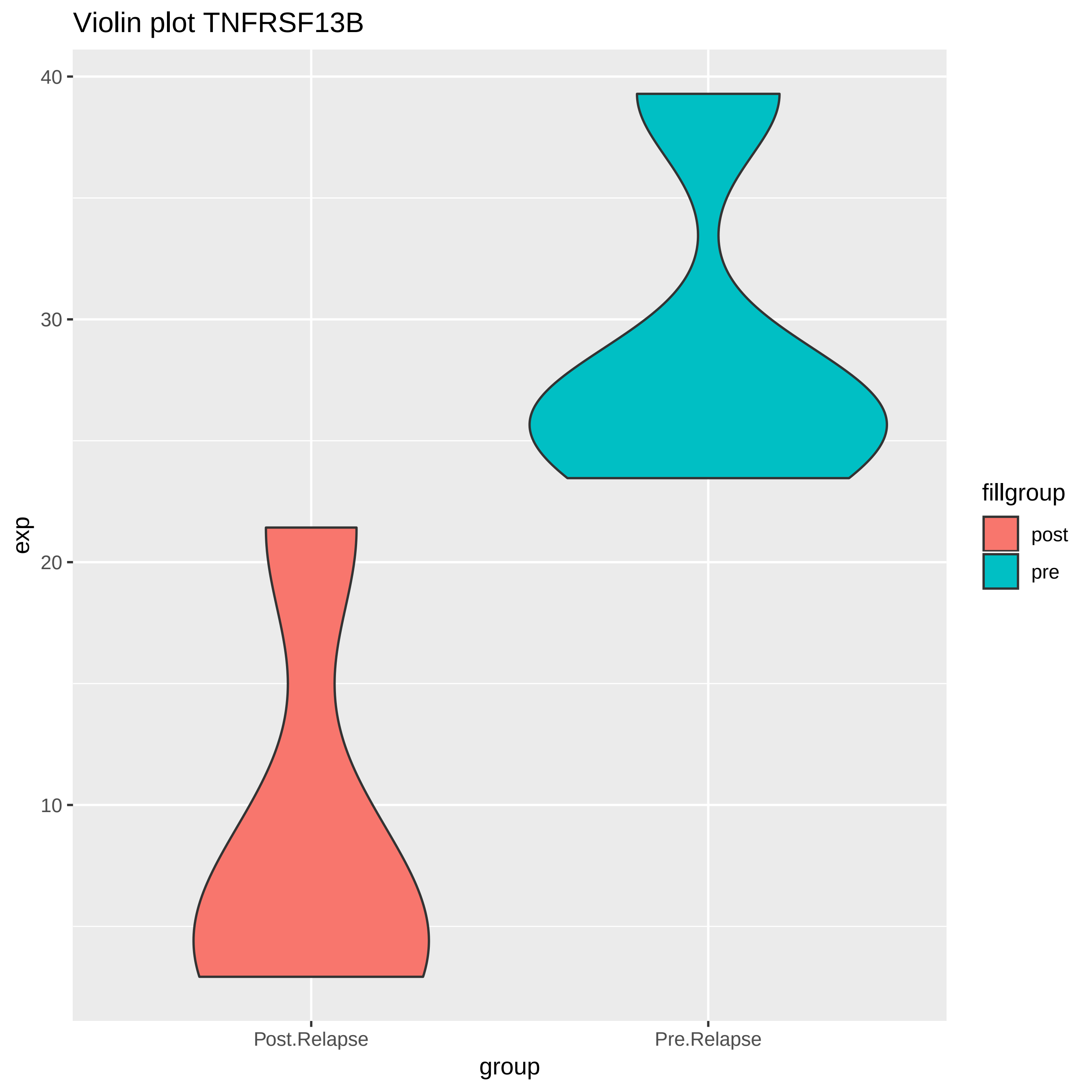

Violin plot TNFRSF13B
40
30
20
10
exp
Post.Relapse
Pre.Relapse
group
fillgroup
post
pre

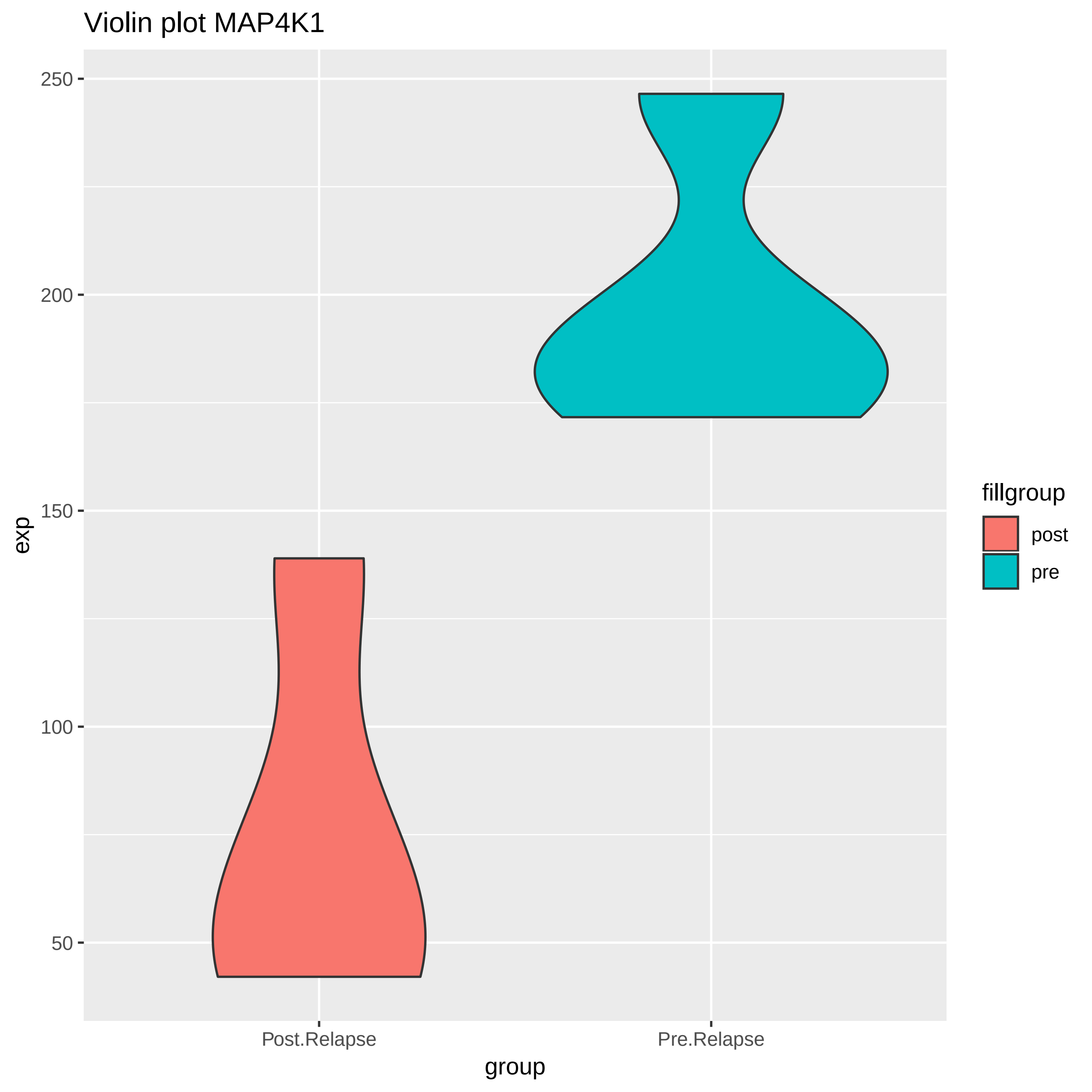

Violin plot MAP4K1
250
200
150
100
50
exp
Post.Relapse
Pre.Relapse
group
fillgroup
post
pre

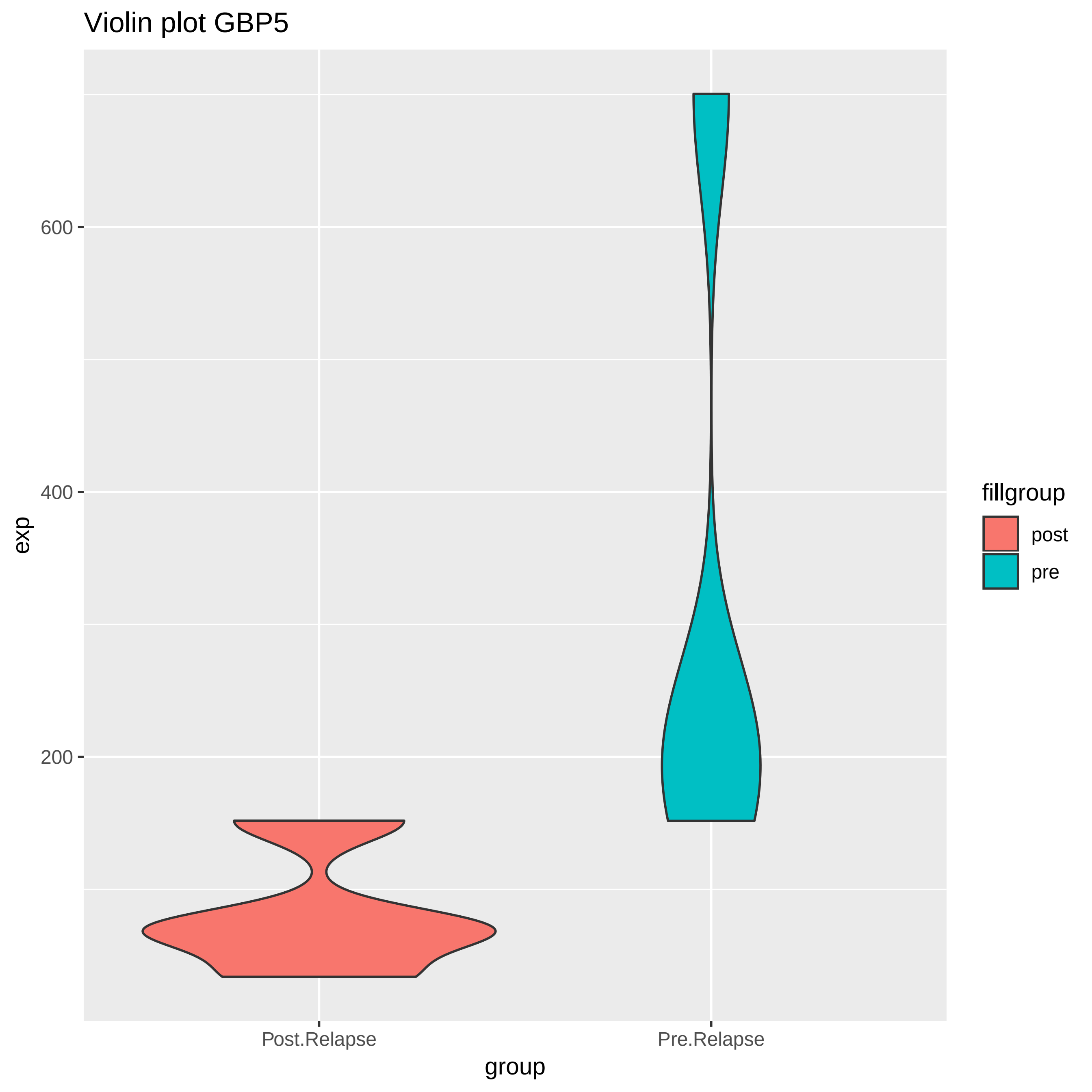
Violin plot GBP5
exp
600
400
200
group
Post.Relapse
Pre.Relapse
fillgroup
post
pre

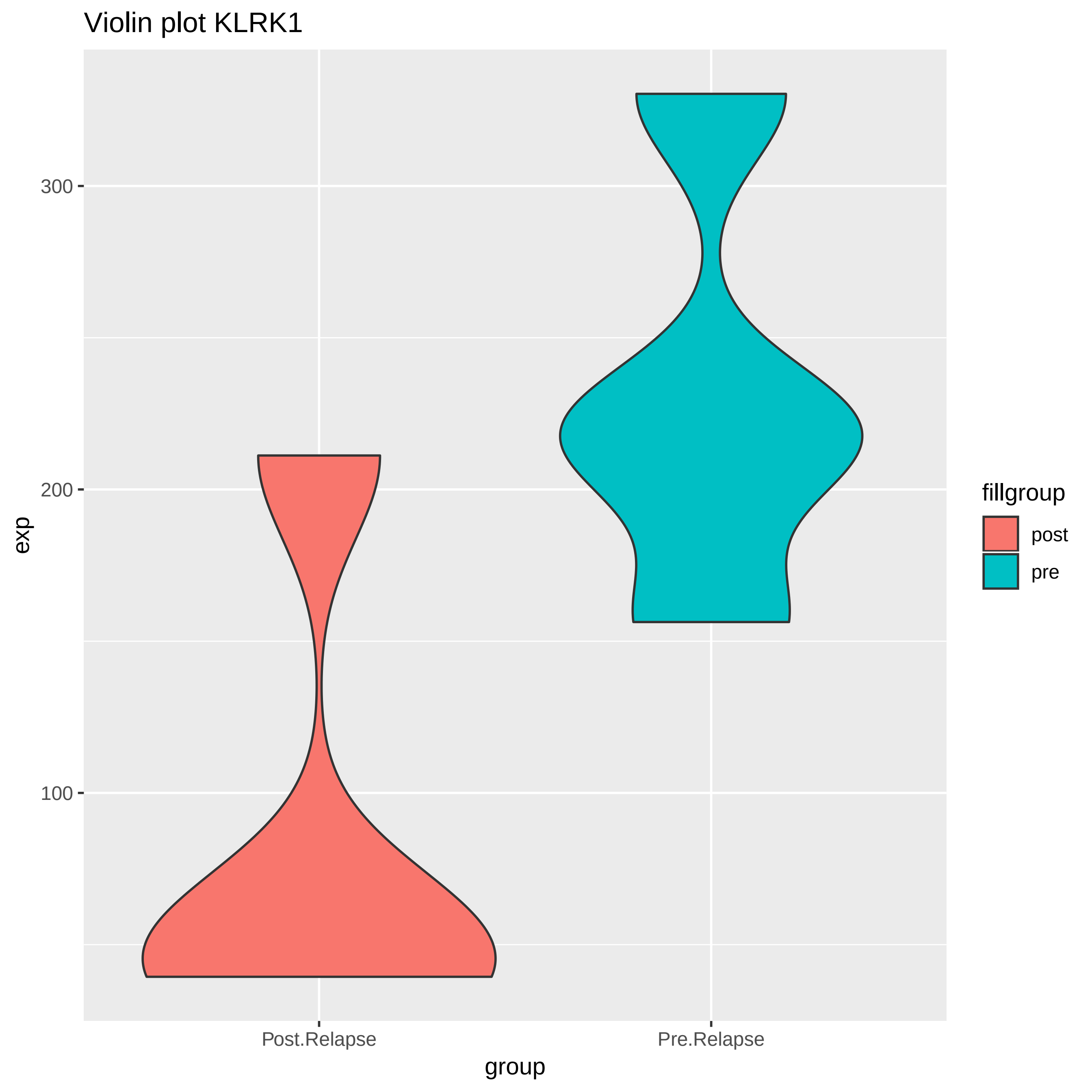

Violin plot KLRK1
300
200
100
exp
Post.Relapse
Pre.Relapse
group
fillgroup
post
pre

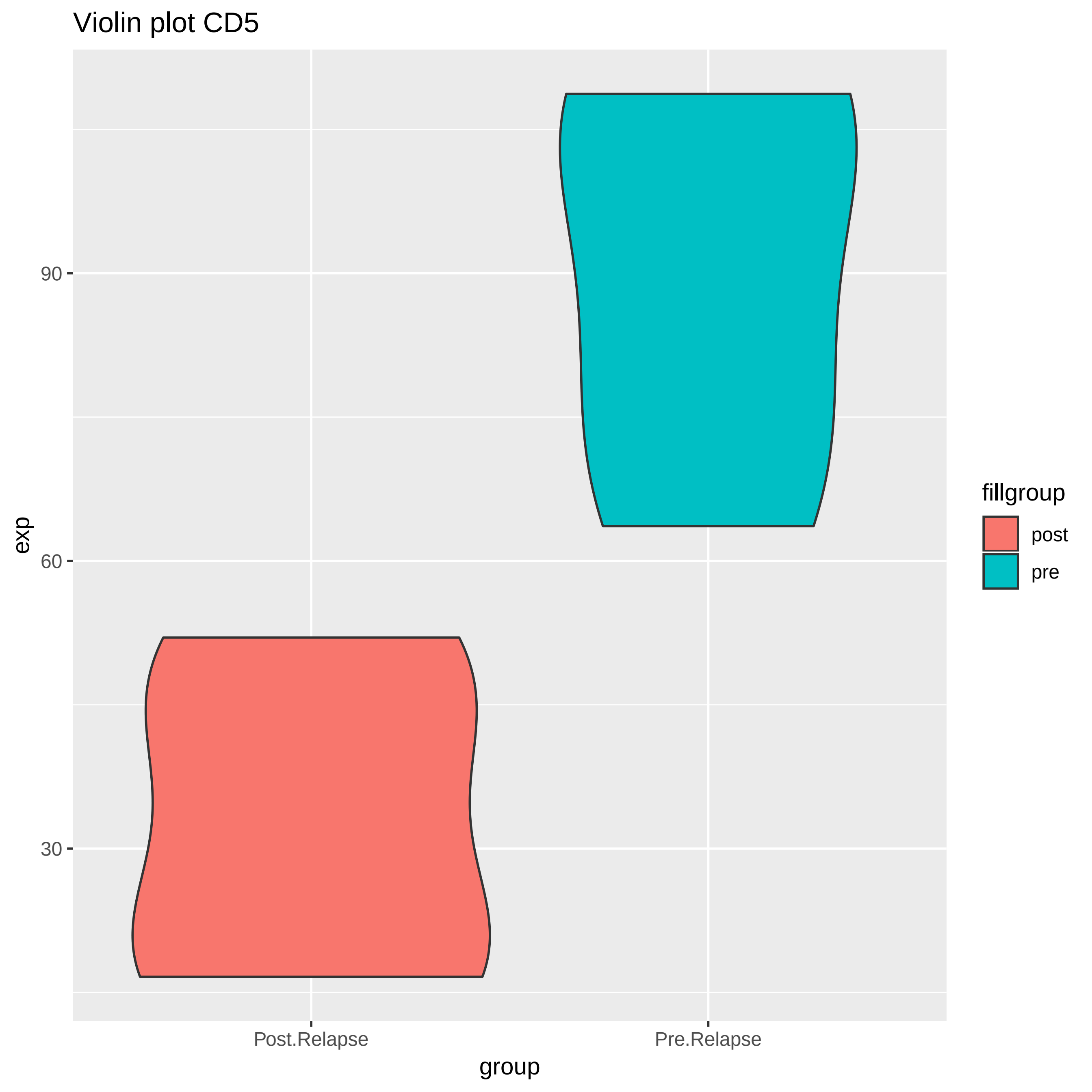

Violin plot CD5
exp
90
60
30
Post.Relapse
Pre.Relapse
group
fillgroup
post
pre

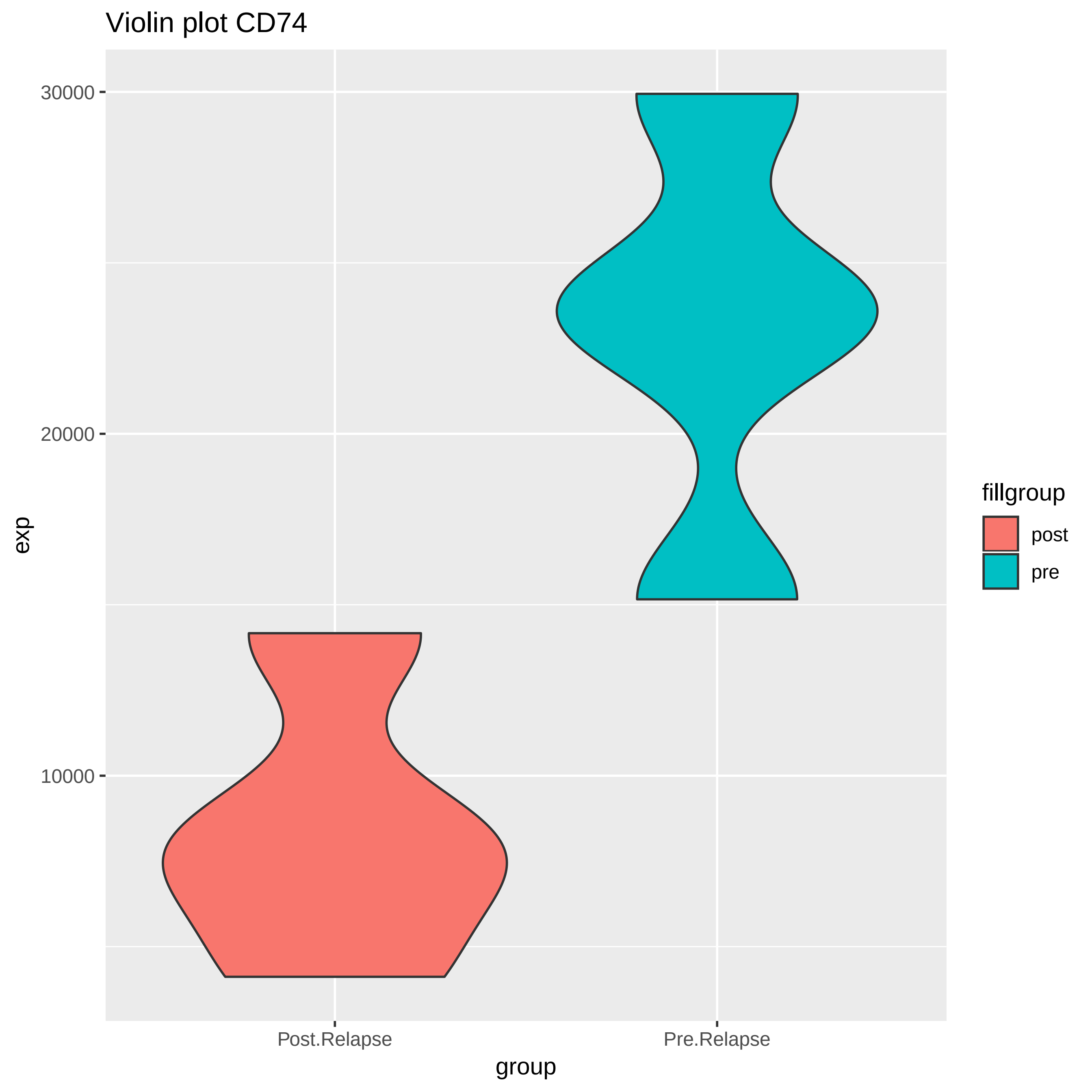

Violin plot CD74
30000
20000
10000
exp
Post.Relapse
Pre.Relapse
group
fillgroup
post
pre

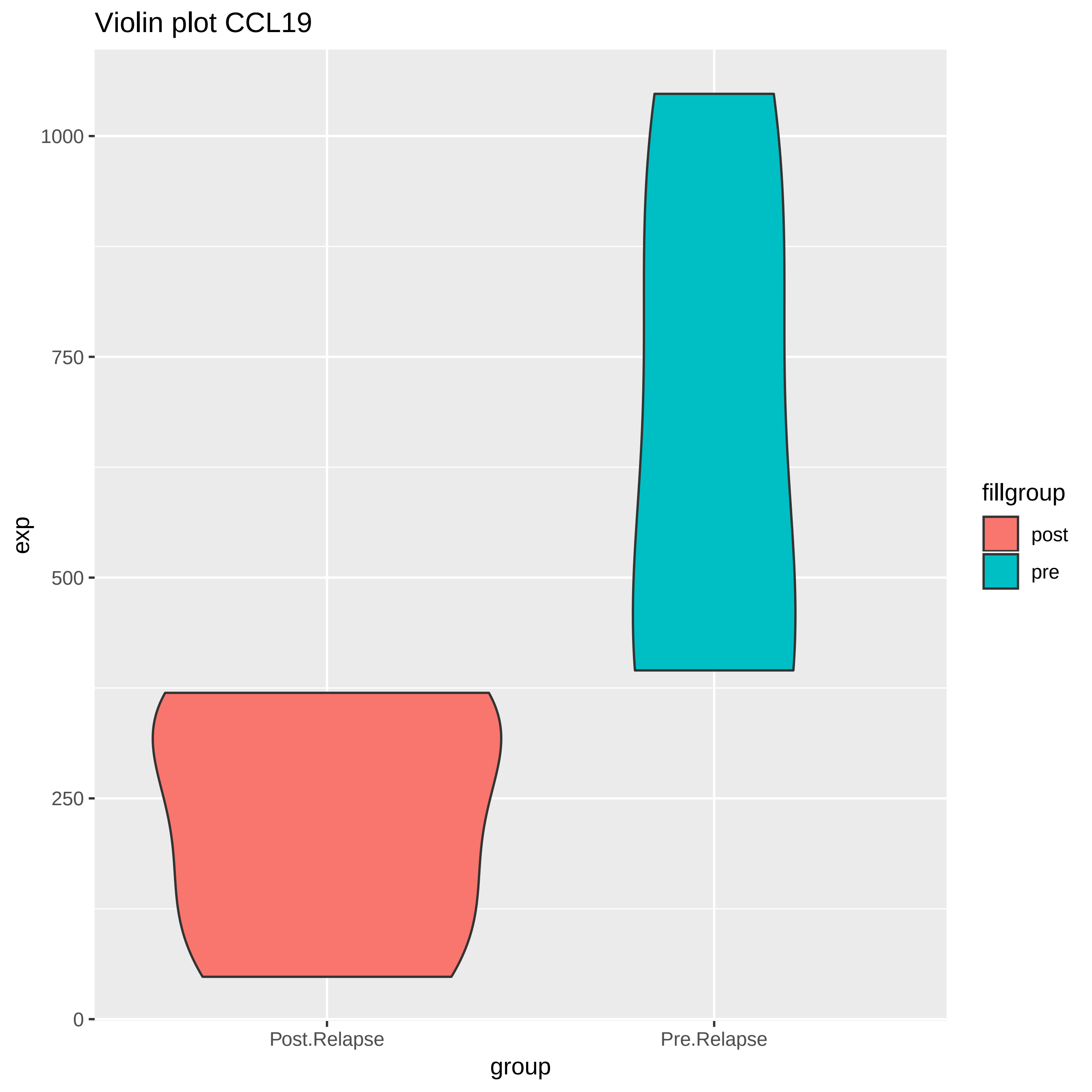
Violin plot CCL19
exp
1000
750
500
250
0
group
Post.Relapse
Pre.Relapse
fillgroup
post
pre

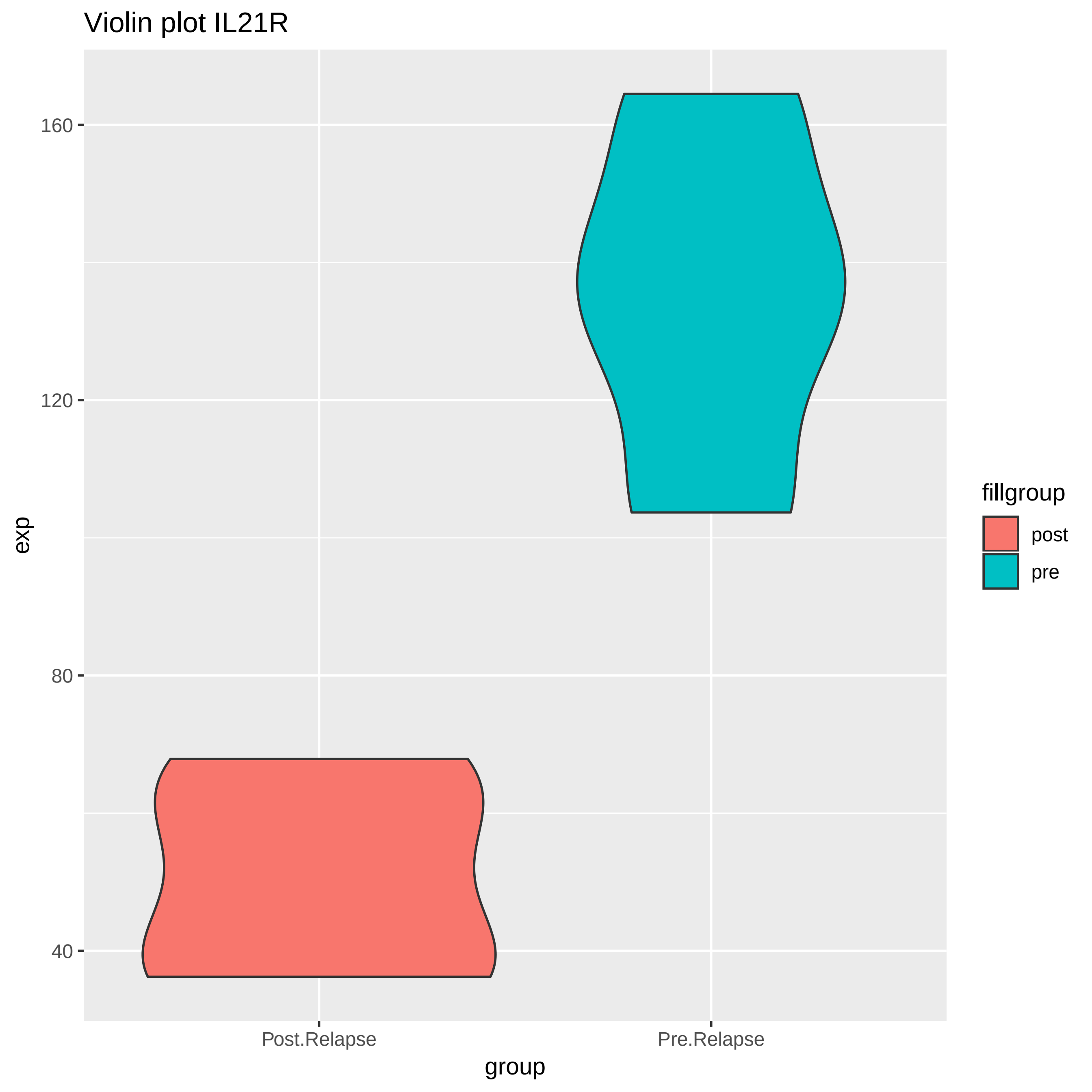

Violin plot IL21R
160
120
80
40
exp
Post.Relapse
Pre.Relapse
group
fillgroup
post
pre

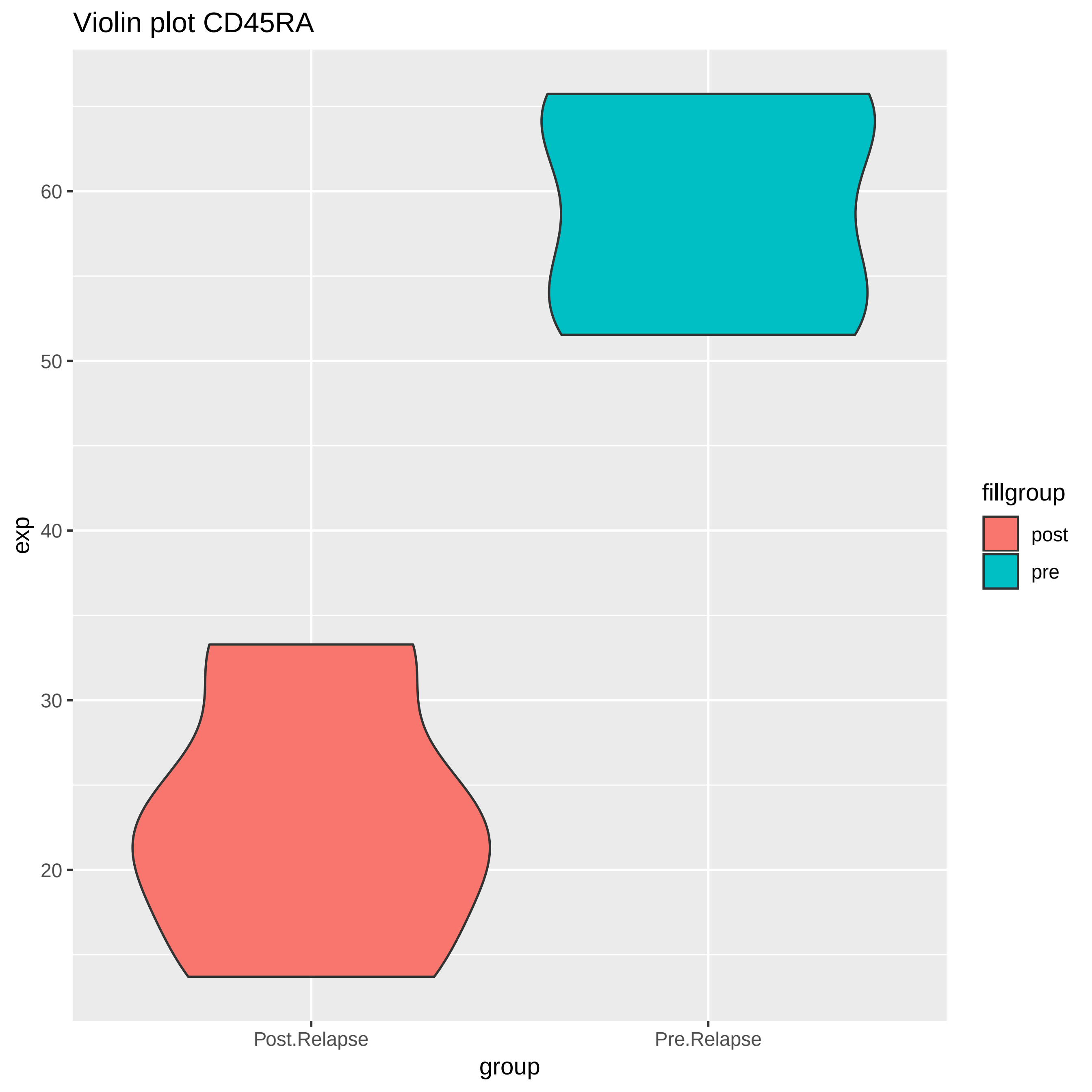

Violin plot CD45RA
exp
60
50
40
30
20
group
Post.Relapse
Pre.Relapse
fillgroup
post
pre

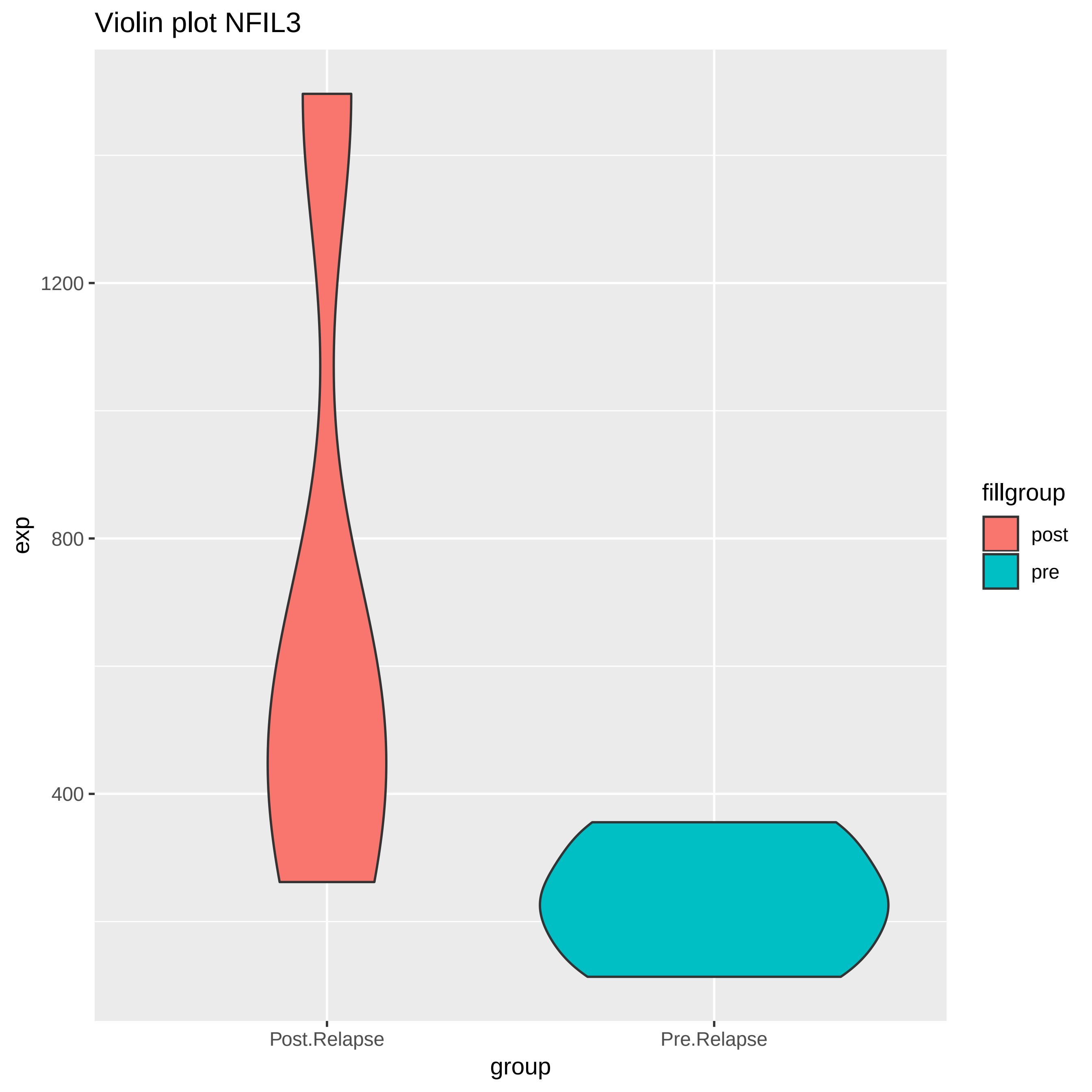

Violin plot NFIL3
1200
800
400
exp
Post.Relapse
Pre.Relapse
group
fillgroup
post
pre

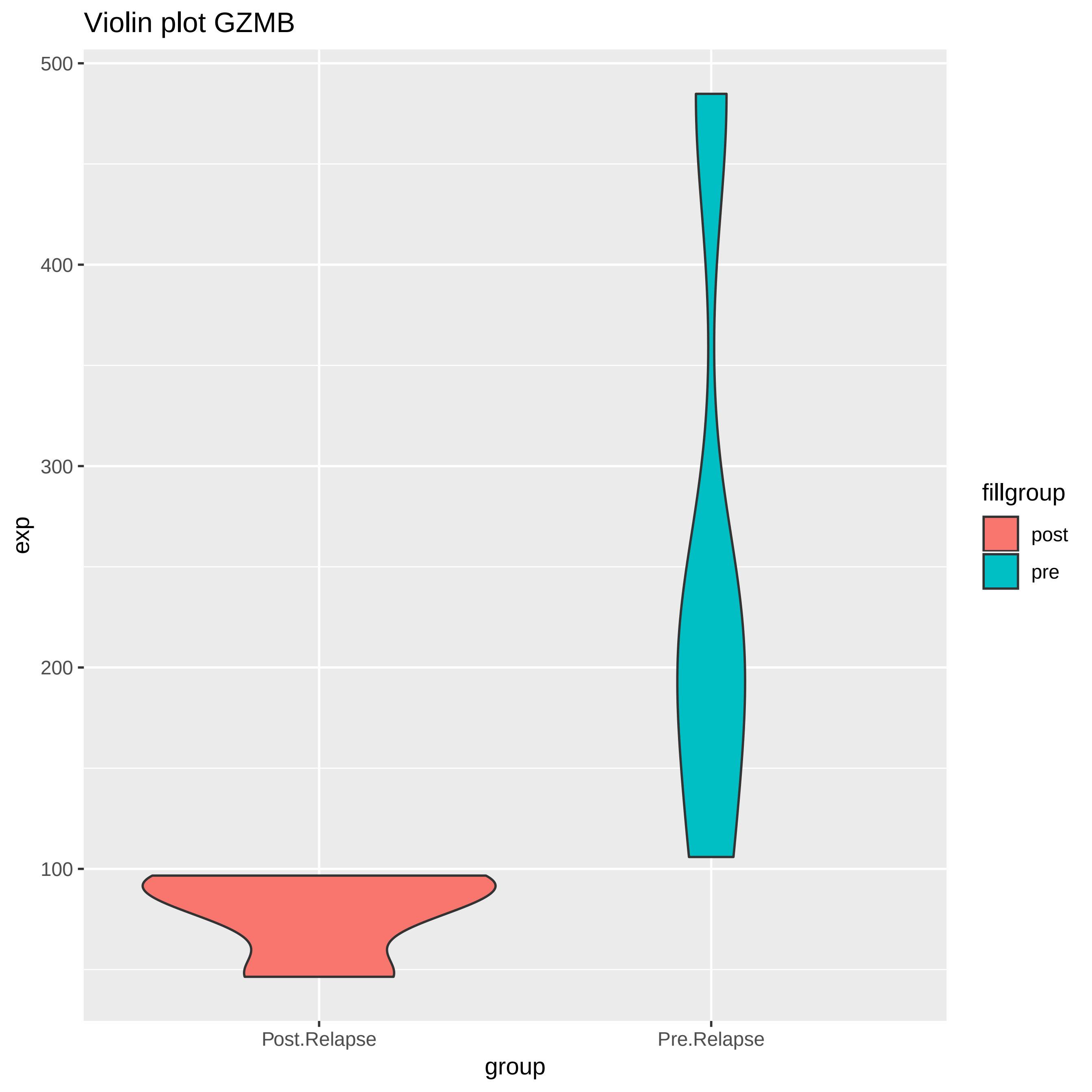

Violin plot GZMB
500
400
300
200
100
exp
Post.Relapse
Pre.Relapse
group
fillgroup
post
pre

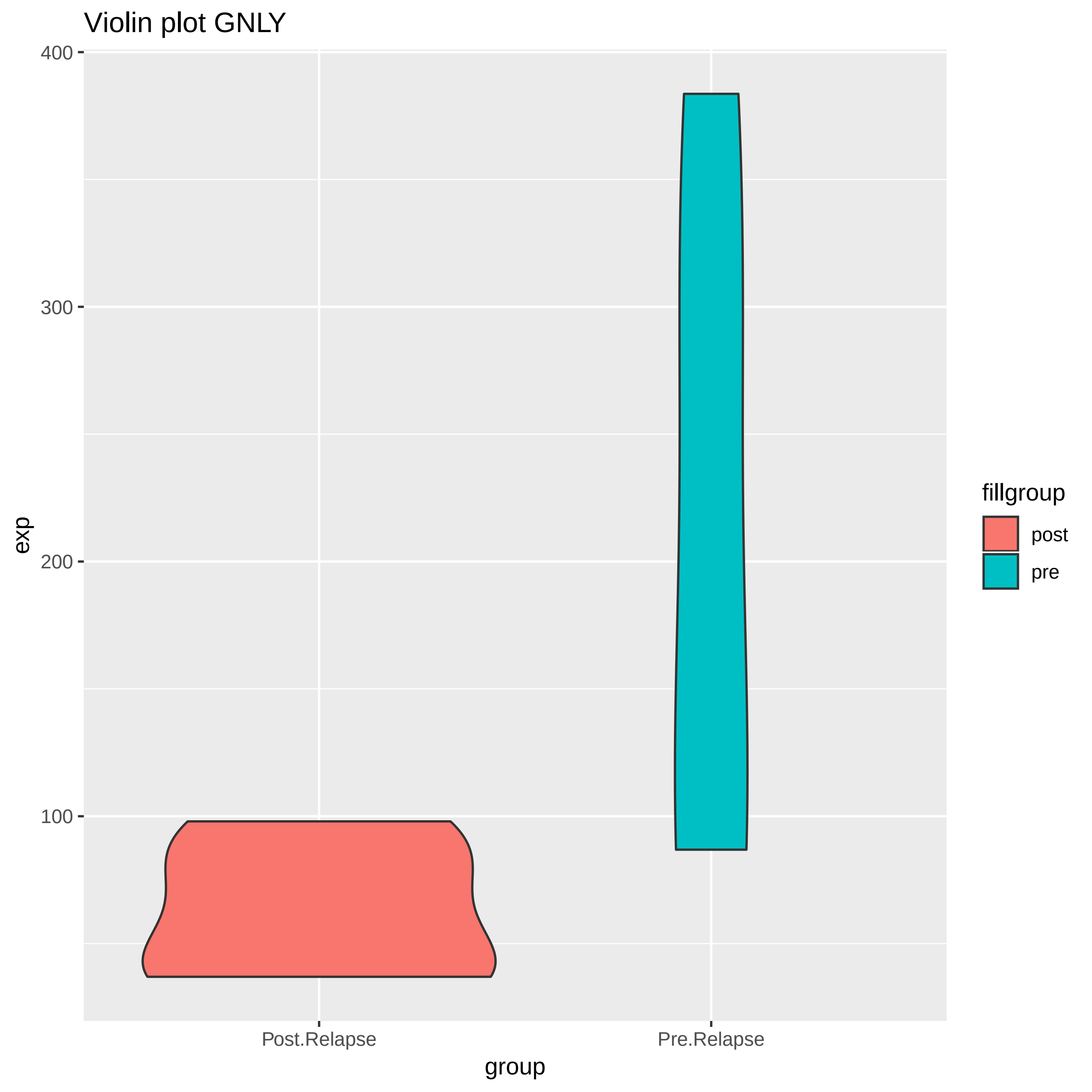

Violin plot GNLY
400
300
200
100
exp
Post.Relapse
Pre.Relapse
group
fillgroup
post
pre

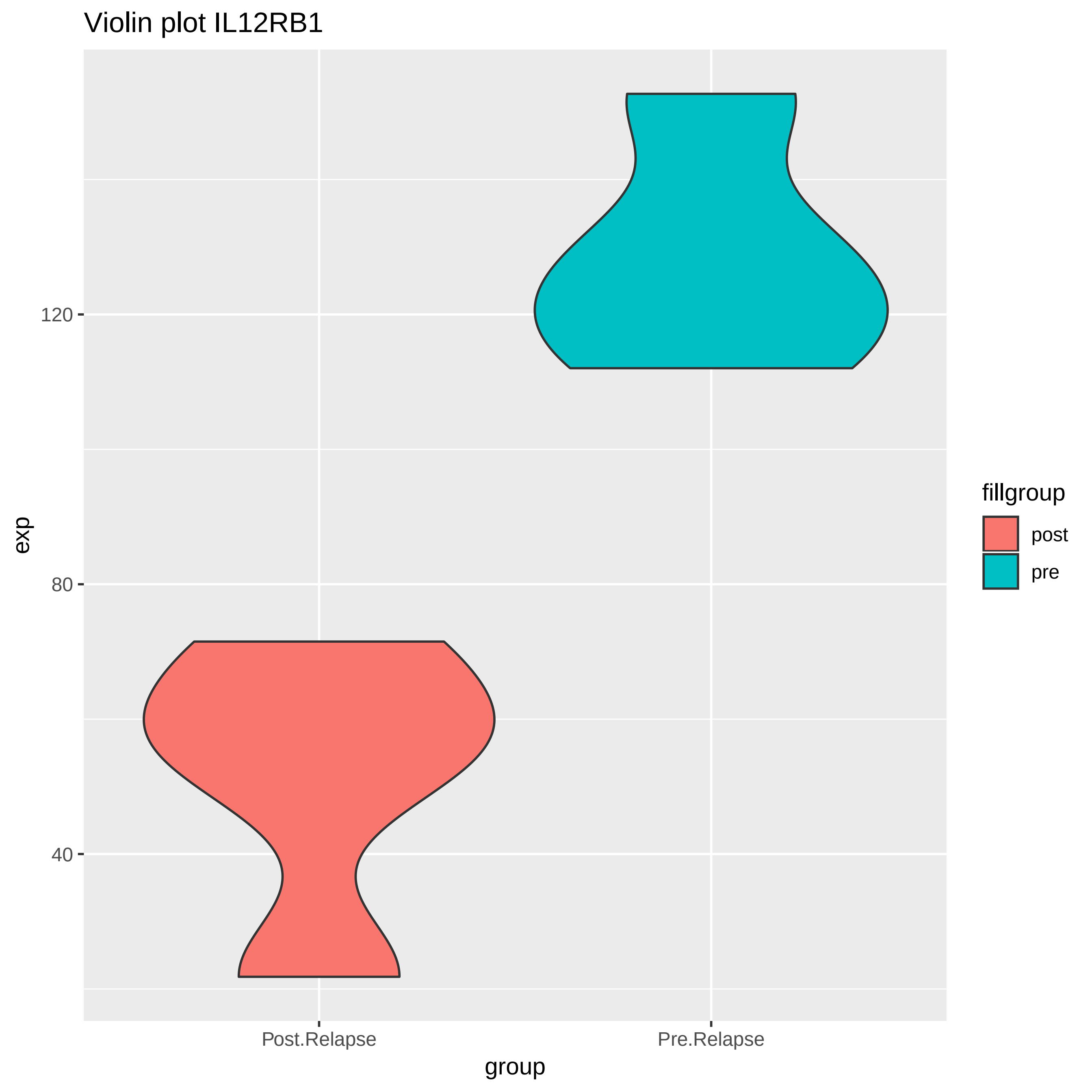

Violin plot IL12RB1
120
80
40
exp
Post.Relapse
Pre.Relapse
group
fillgroup
post
pre

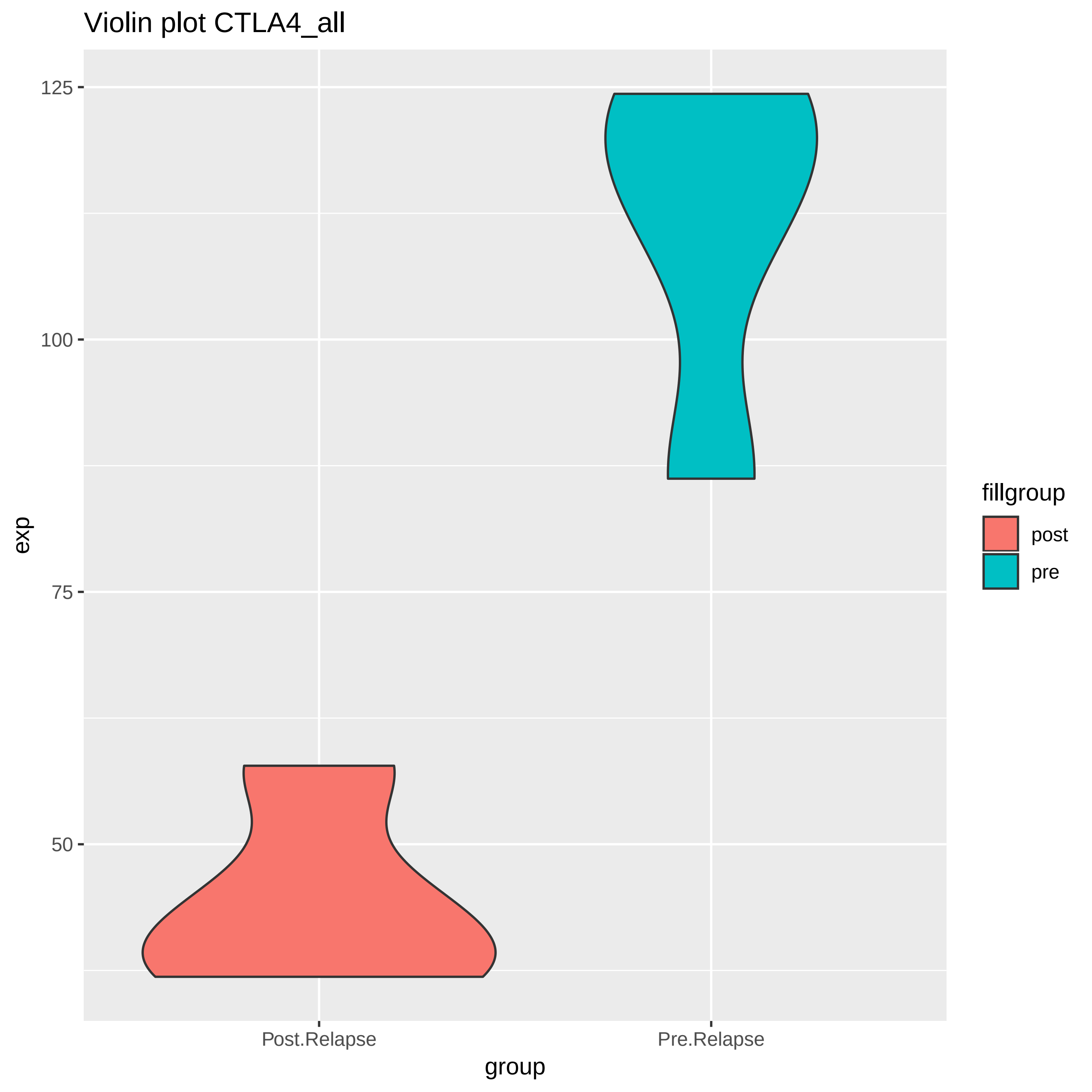

Violin plot CTLA4_all
125
100
75
50
exp
Post.Relapse
Pre.Relapse
group
fillgroup
post
pre

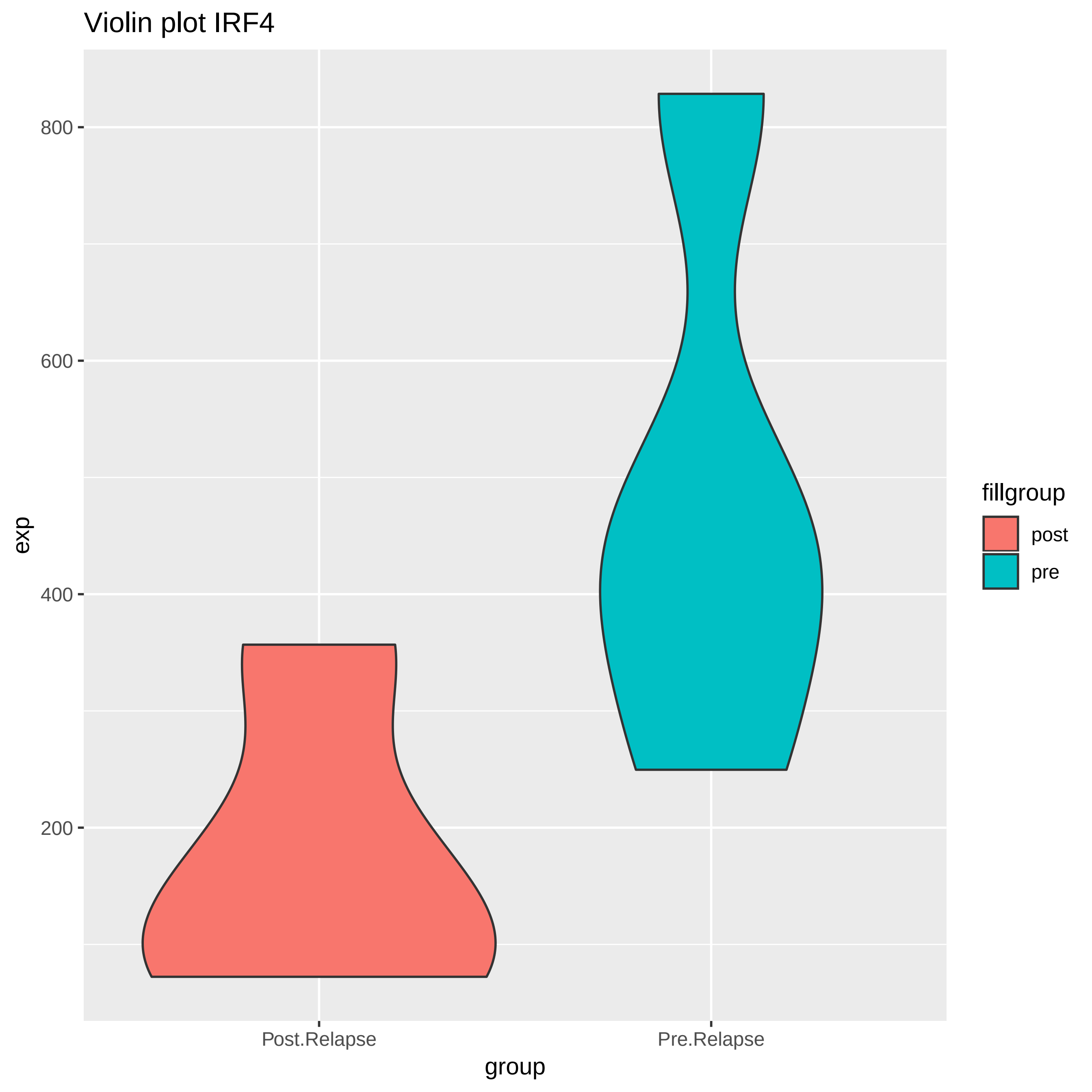
Violin plot IRF4
exp
800
600
400
200
Post.Relapse
Pre.Relapse
group
fillgroup
post
pre

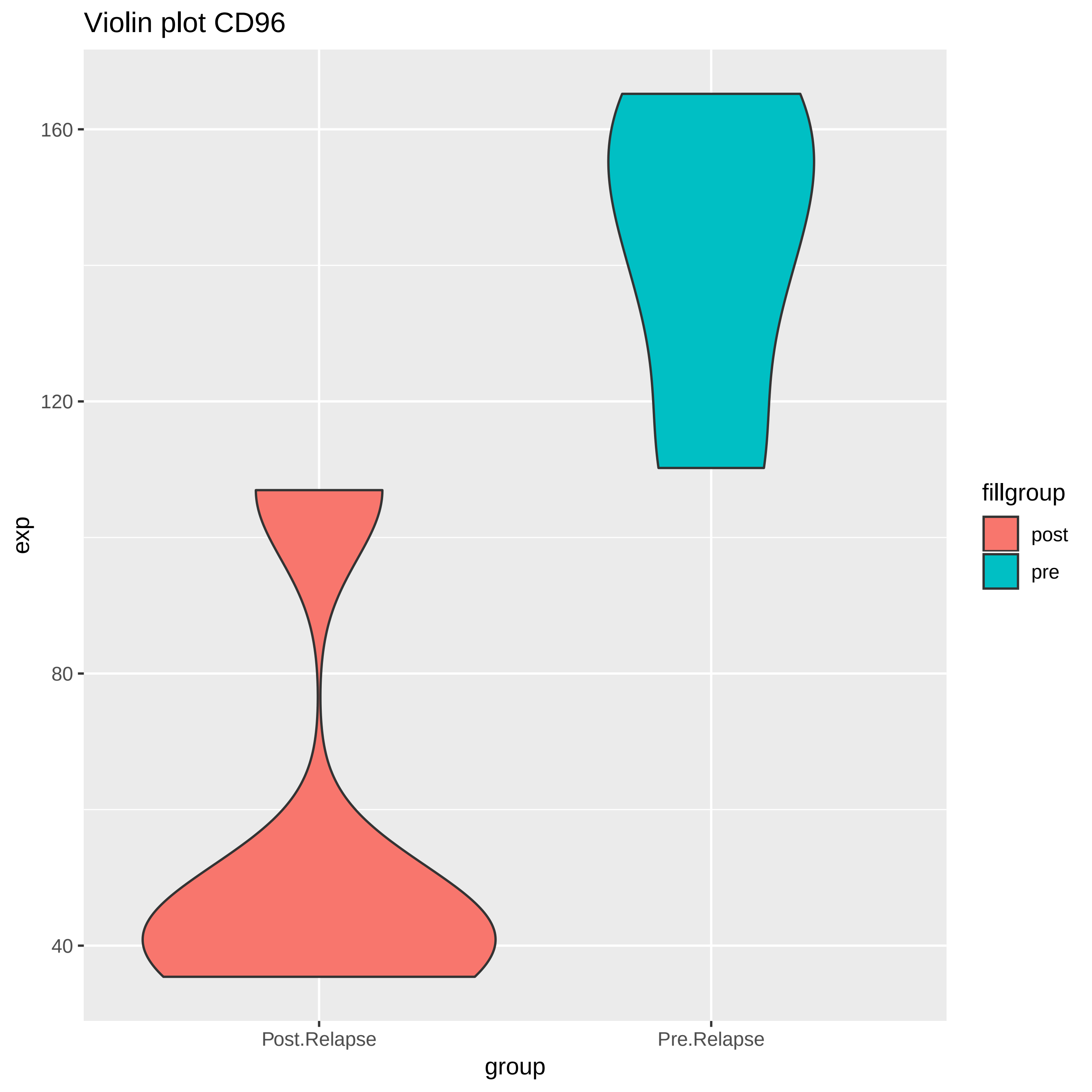

Violin plot CD96
160
120
80
40
exp
Post.Relapse
Pre.Relapse
group
fillgroup
post
pre

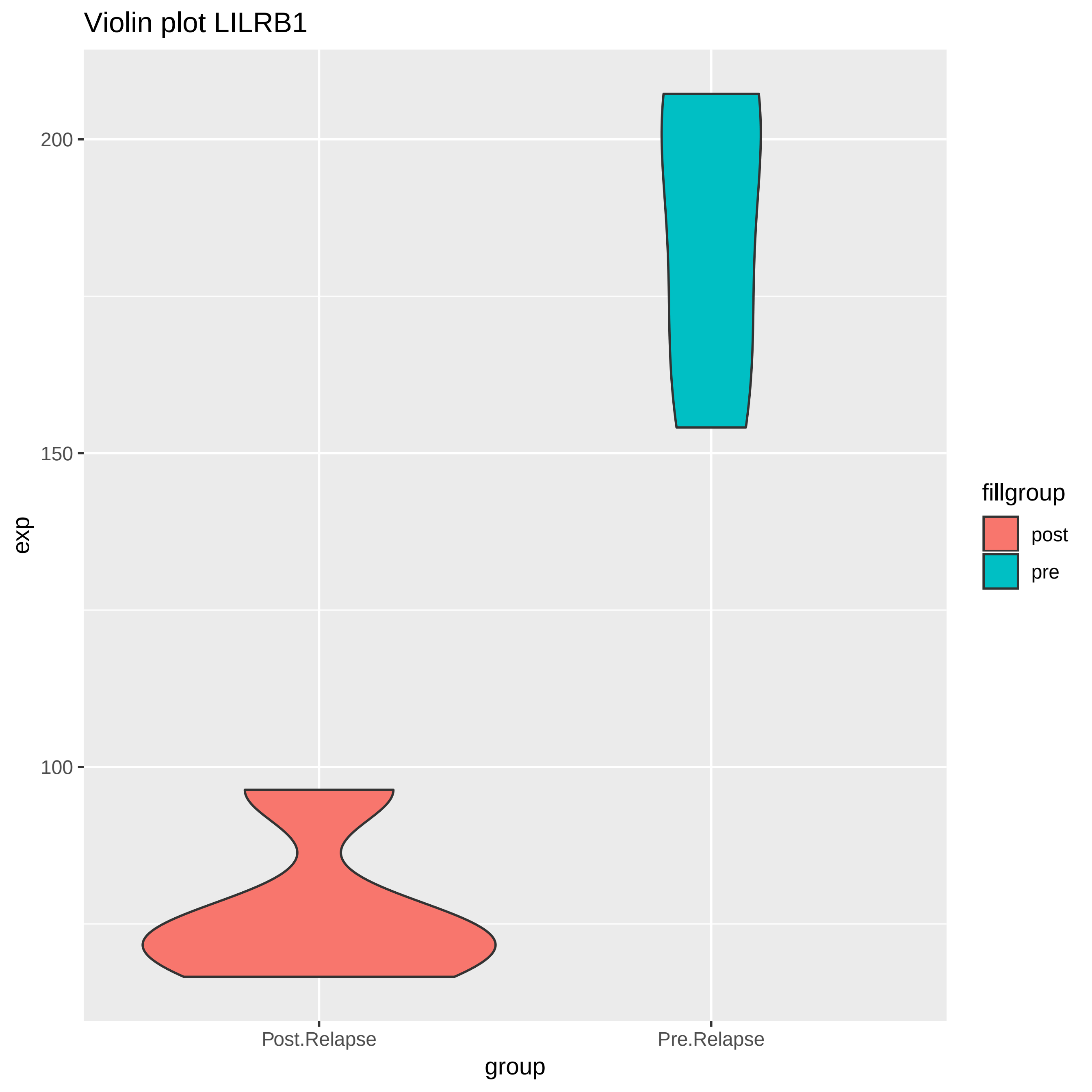
Violin plot LILRB1
200
150
100
exp
Post.Relapse
Pre.Relapse
group
fillgroup
post
pre

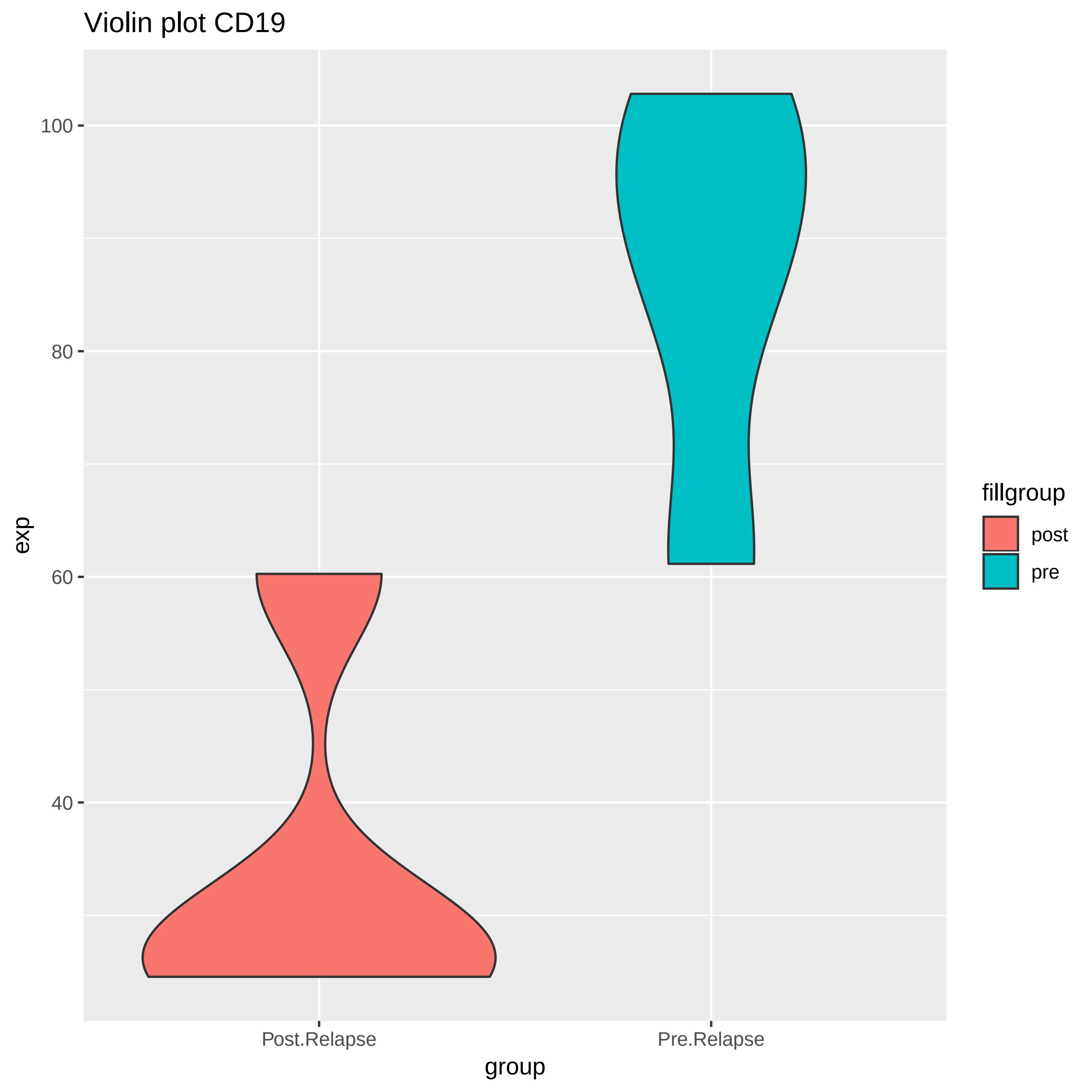

Violin plot CD19
100
80
60
40
exp
Post.Relapse
Pre.Relapse
group
fillgroup
post
pre

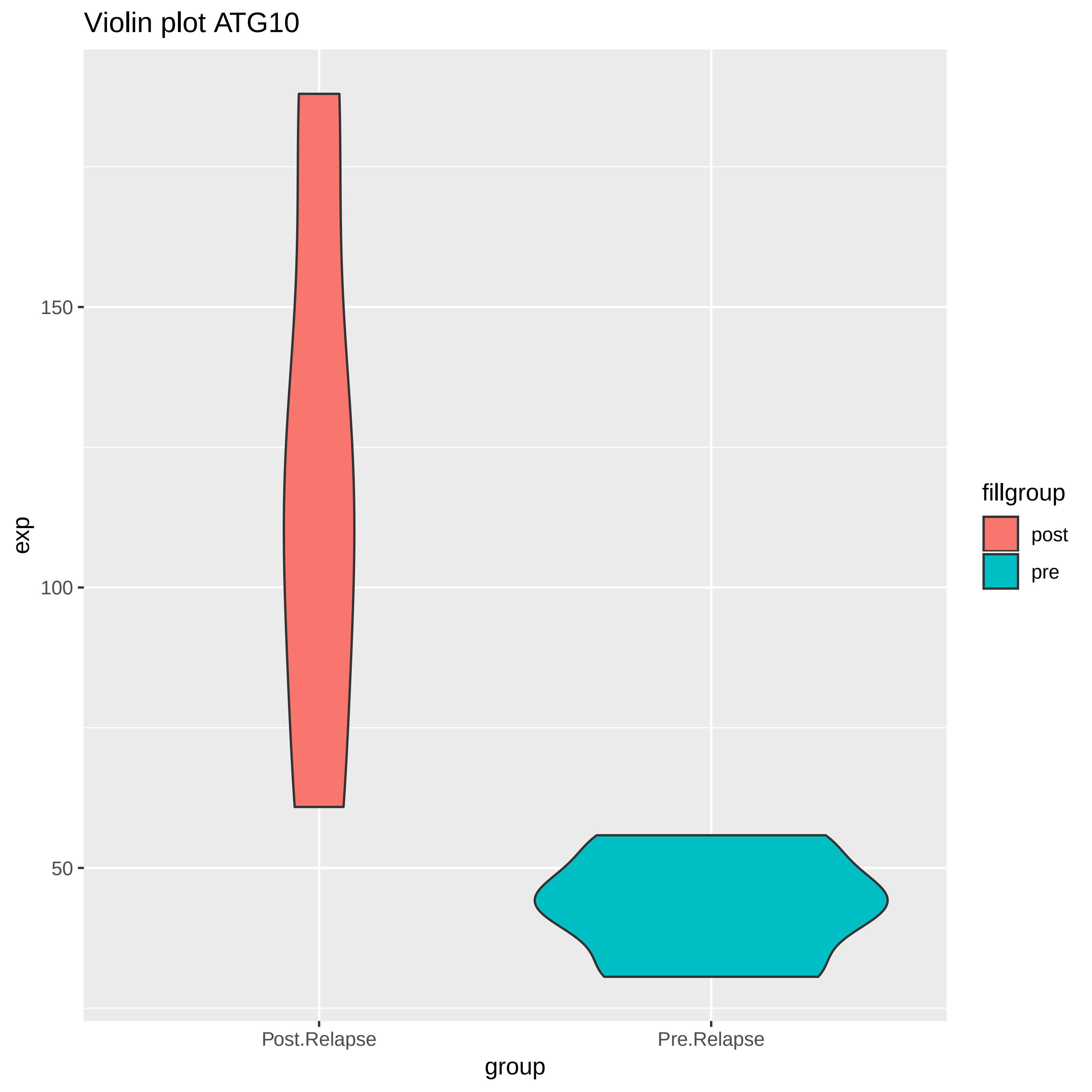
Violin plot ATG10
150
100
50
exp
Post.Relapse
Pre.Relapse
group
fillgroup
post
pre

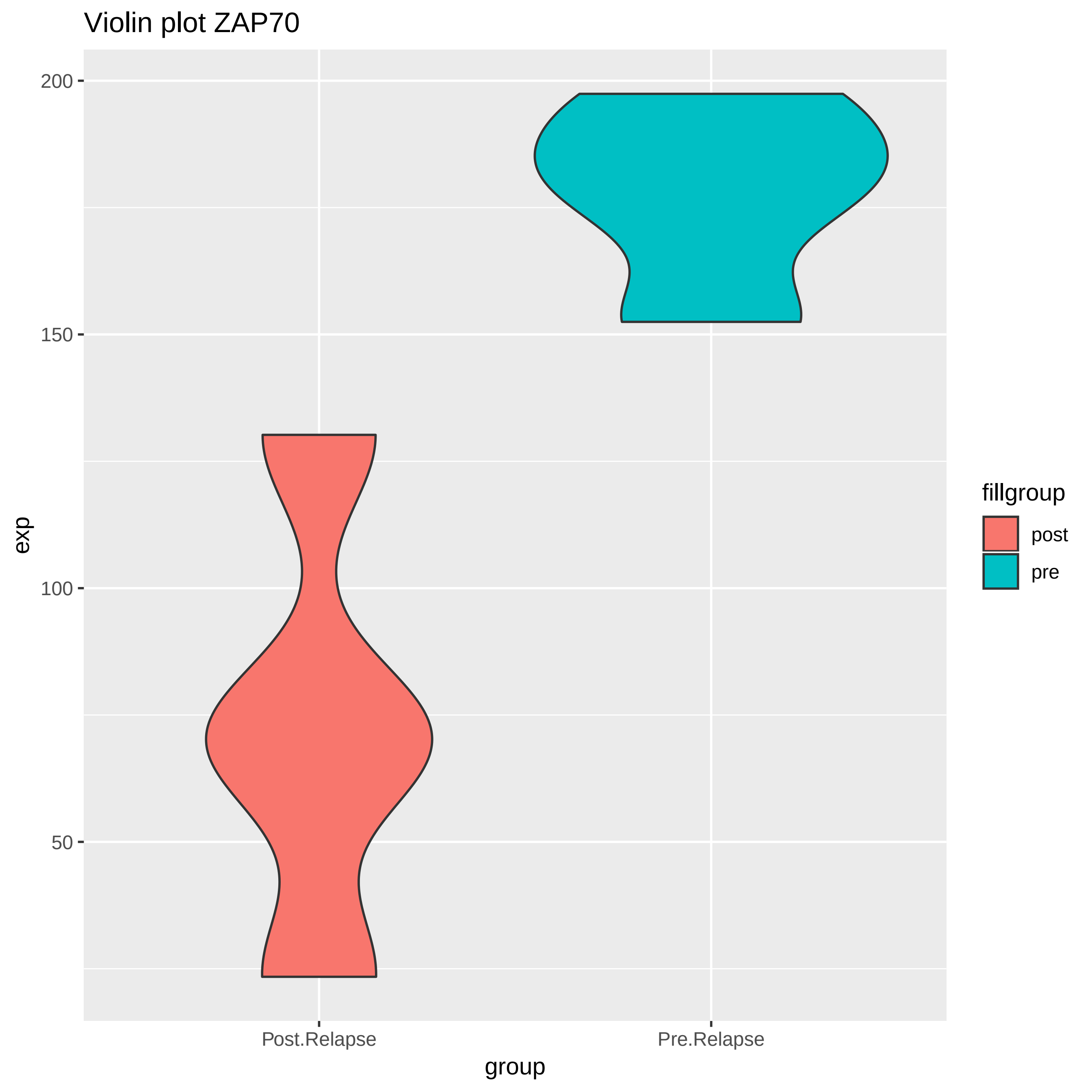
Violin plot ZAP70
200
150
100
50
exp
Post.Relapse
Pre.Relapse
group
fillgroup
post
pre

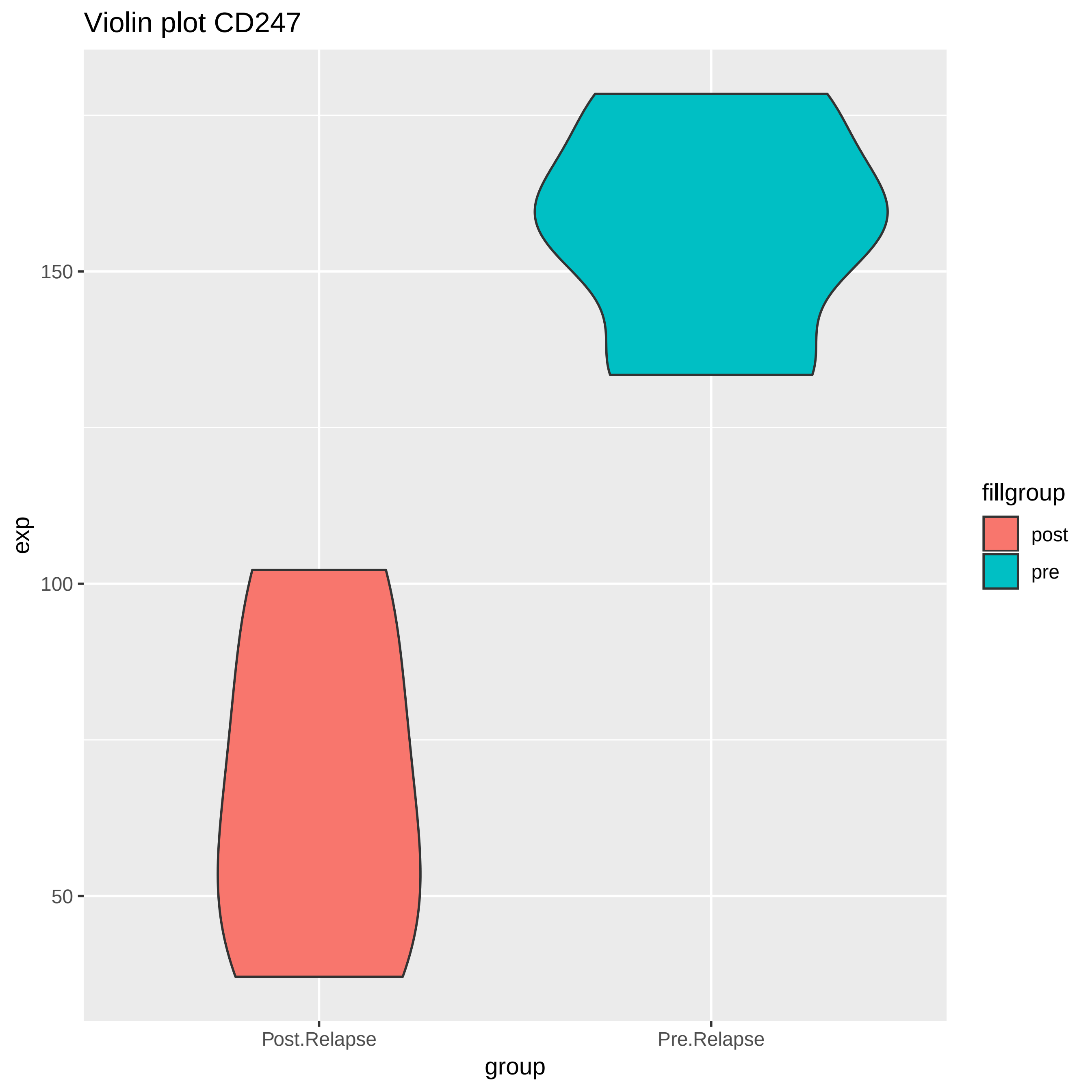

Violin plot CD247
exp
150
100
50
group
Post.Relapse
Pre.Relapse
fillgroup
post
pre
