# Supplementary figures and images for: Prediction of prognostic signatures in triple-negative breast cancer based on the differential expression analysis via NanoString nCounter immune panel
Source: BMC Cancer. 2020 Nov 2;20:1052. doi: 10.1186/s12885-020-07399-8 (PMC7607642; doi:10.1186/s12885-020-07399-8)

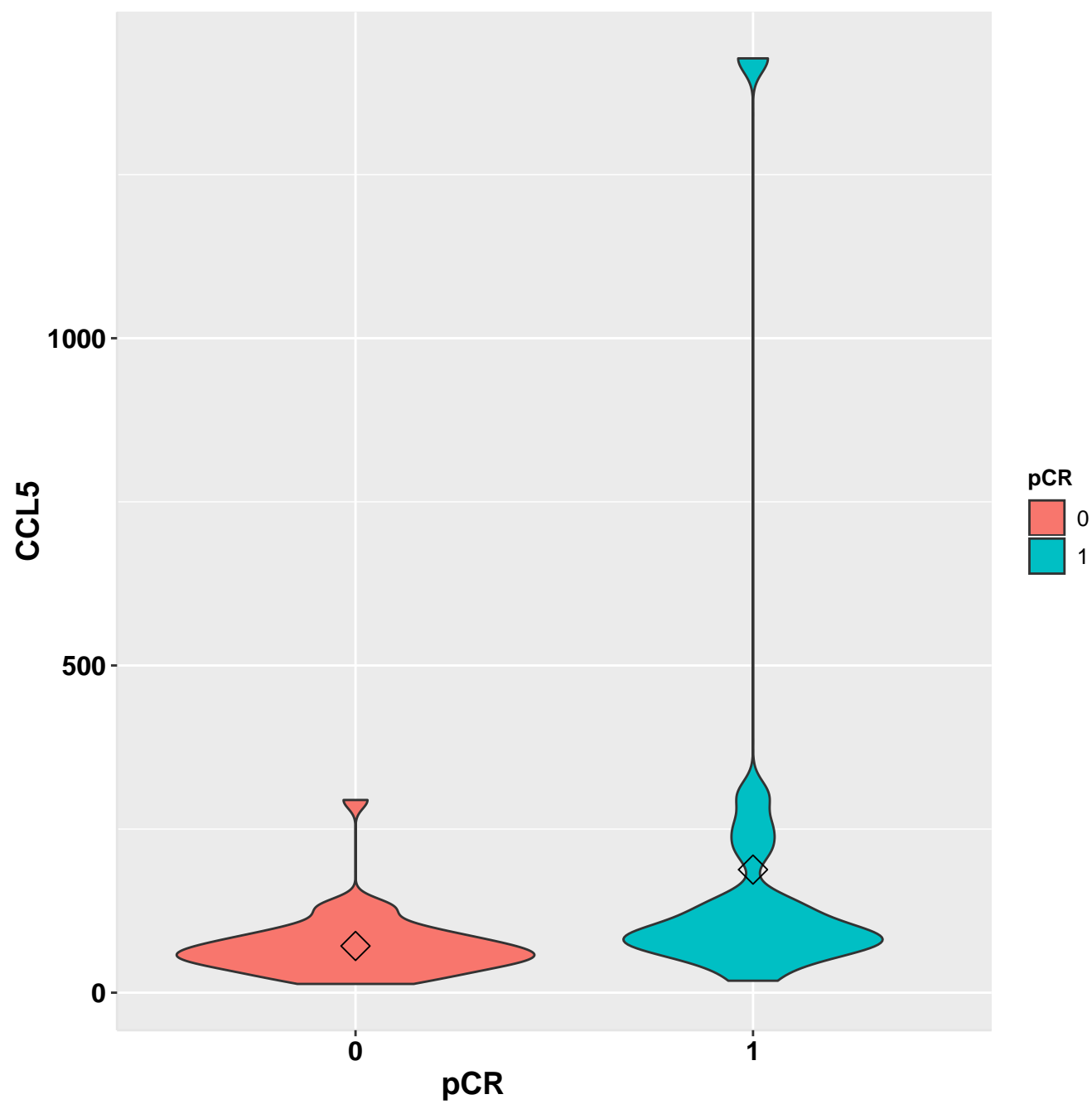

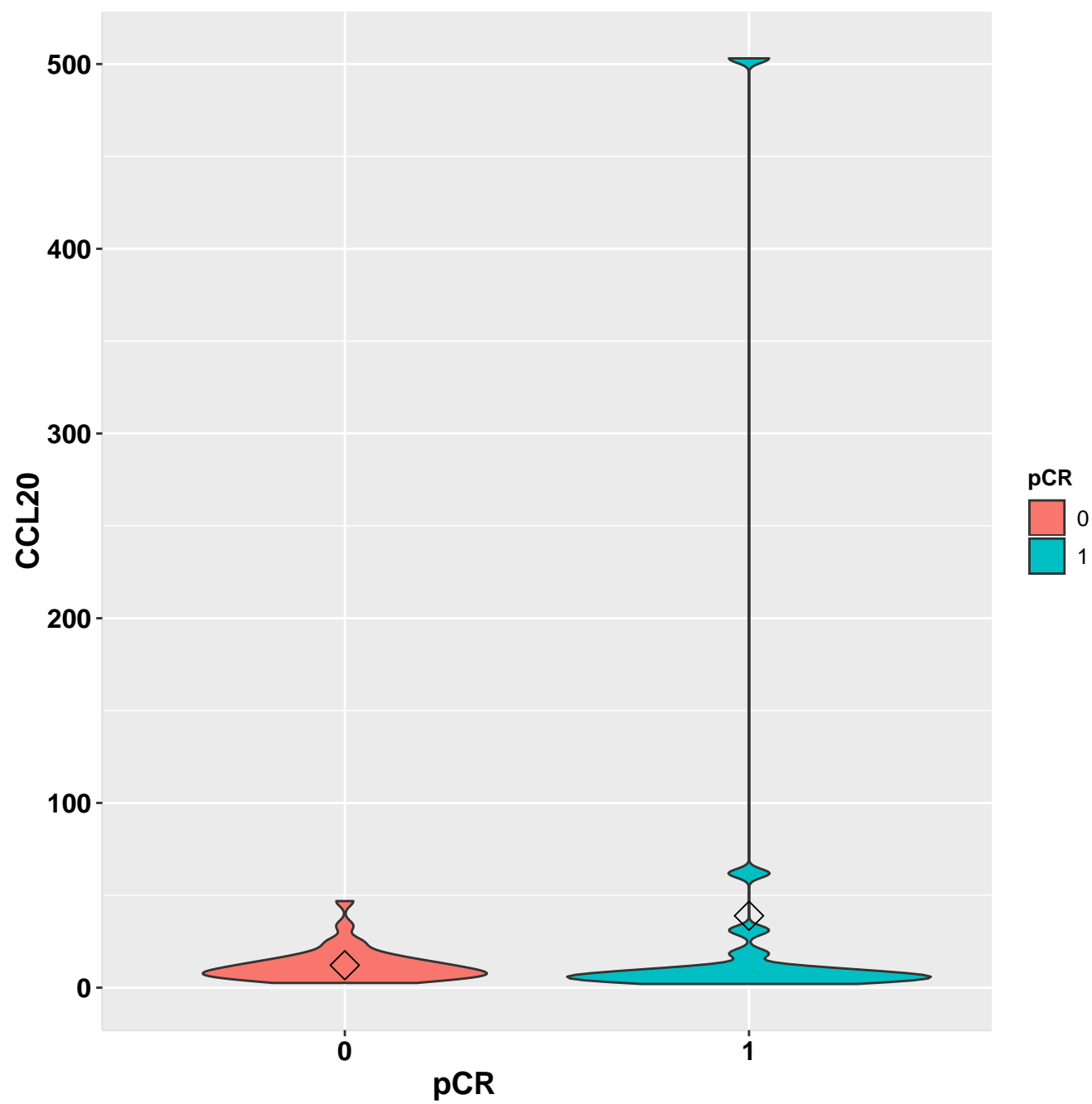

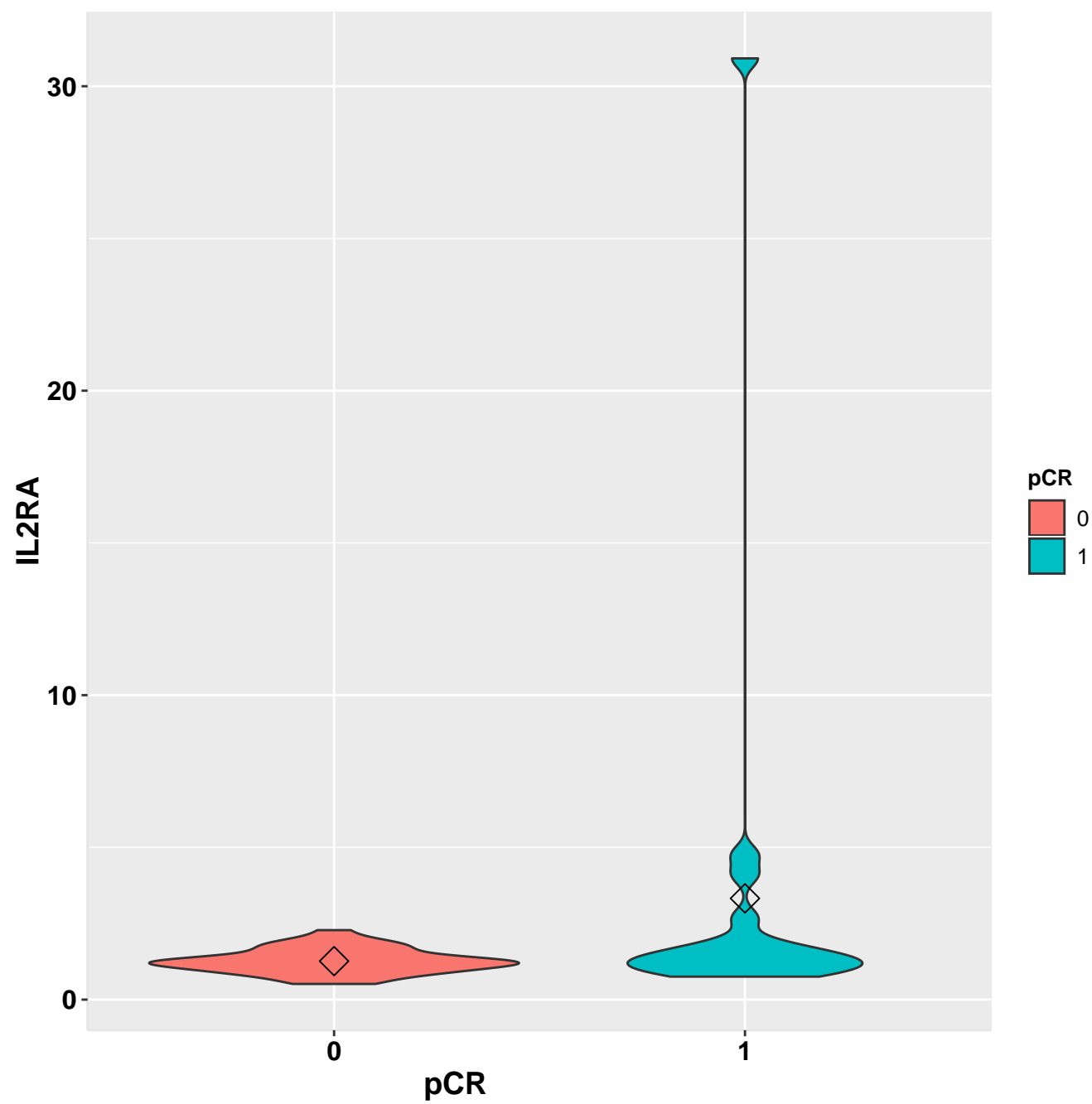

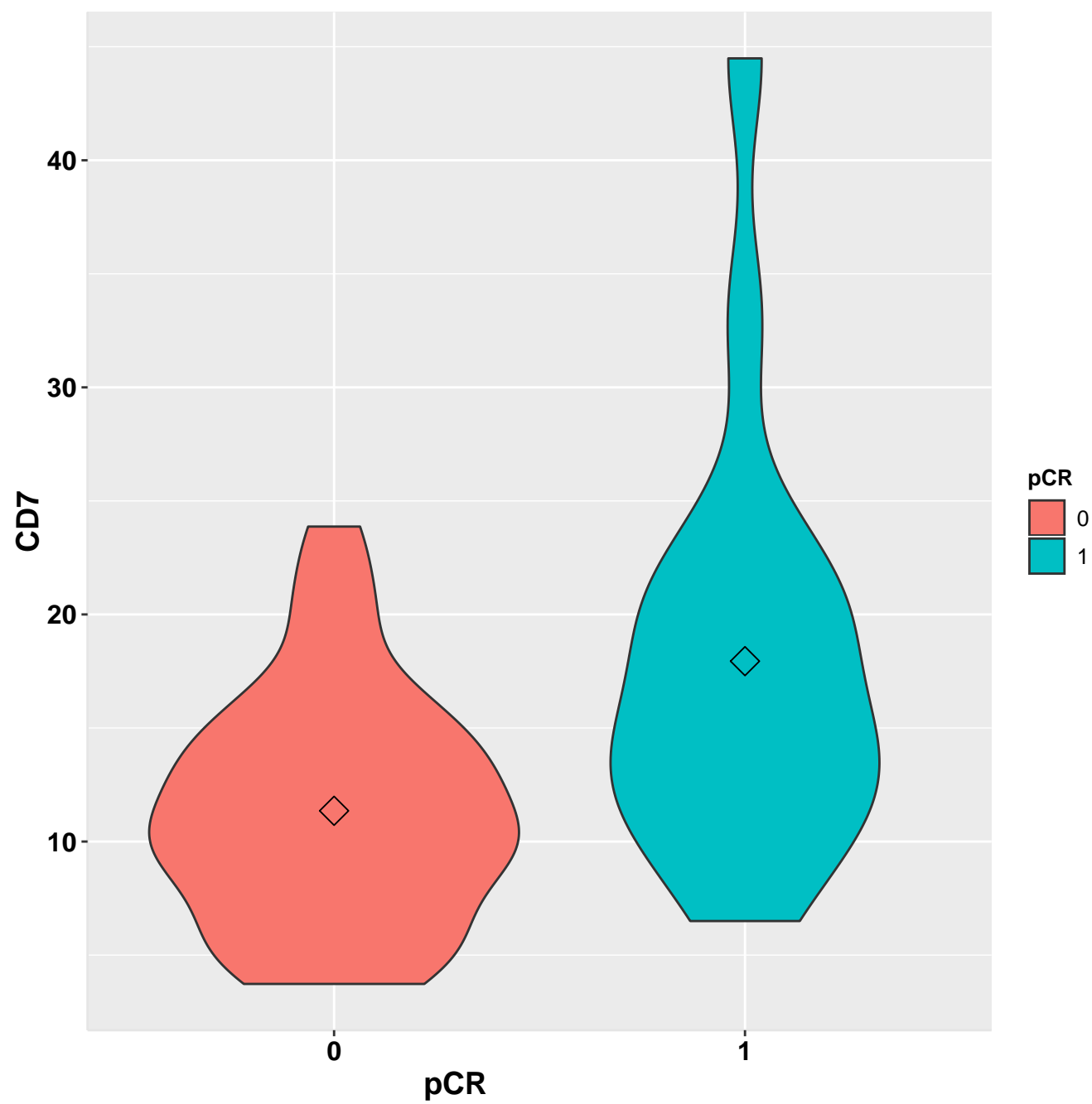

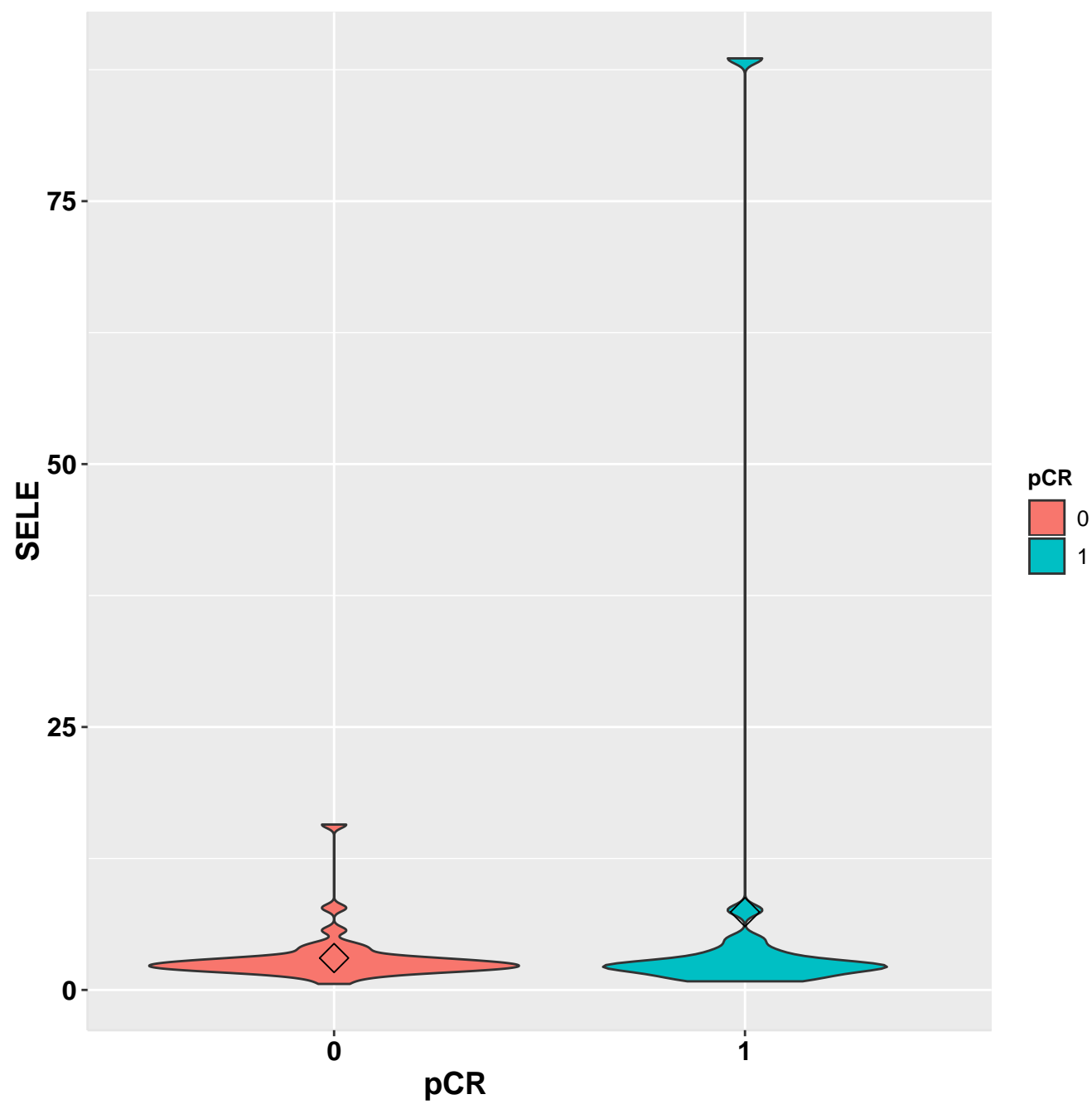

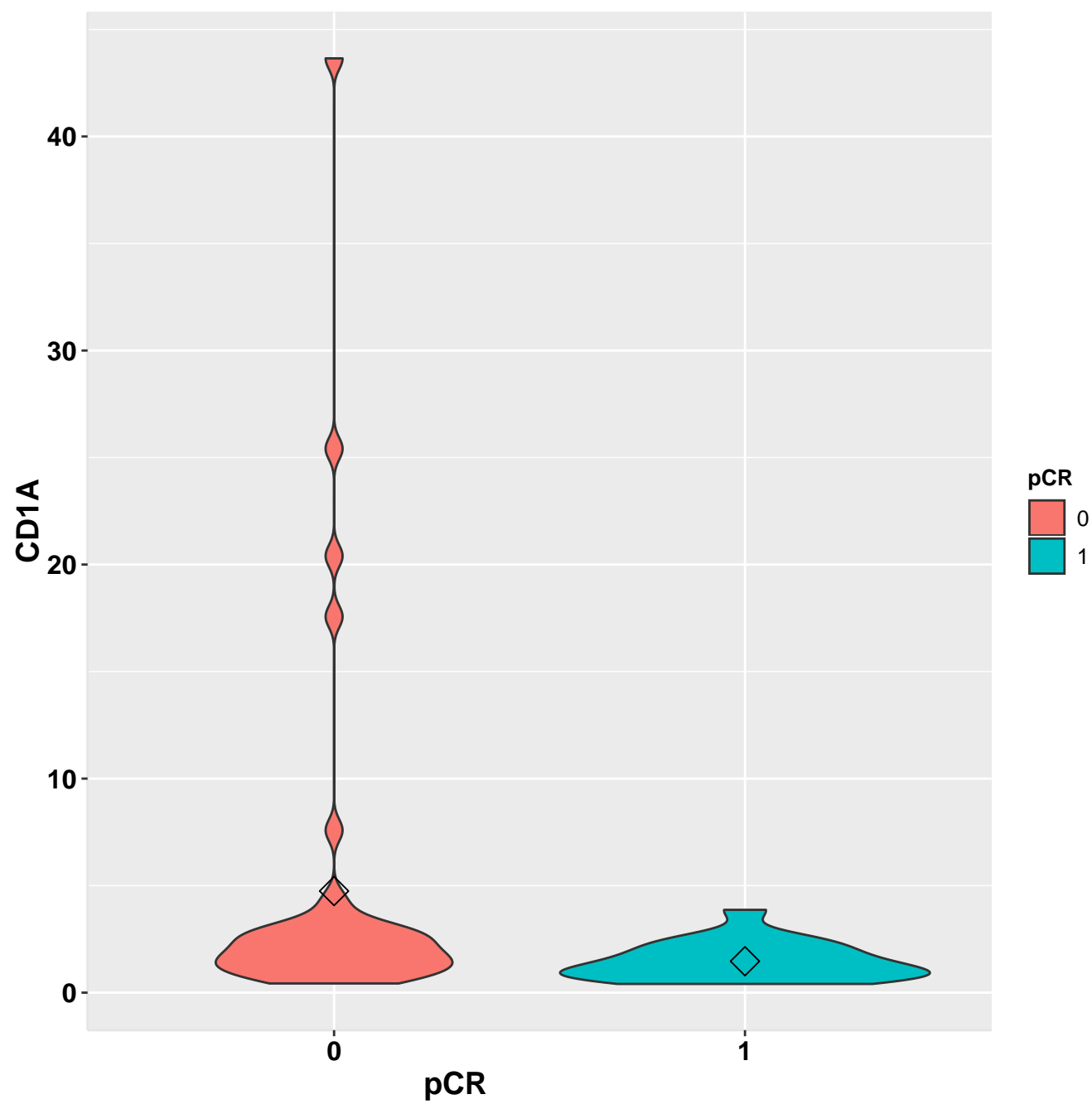

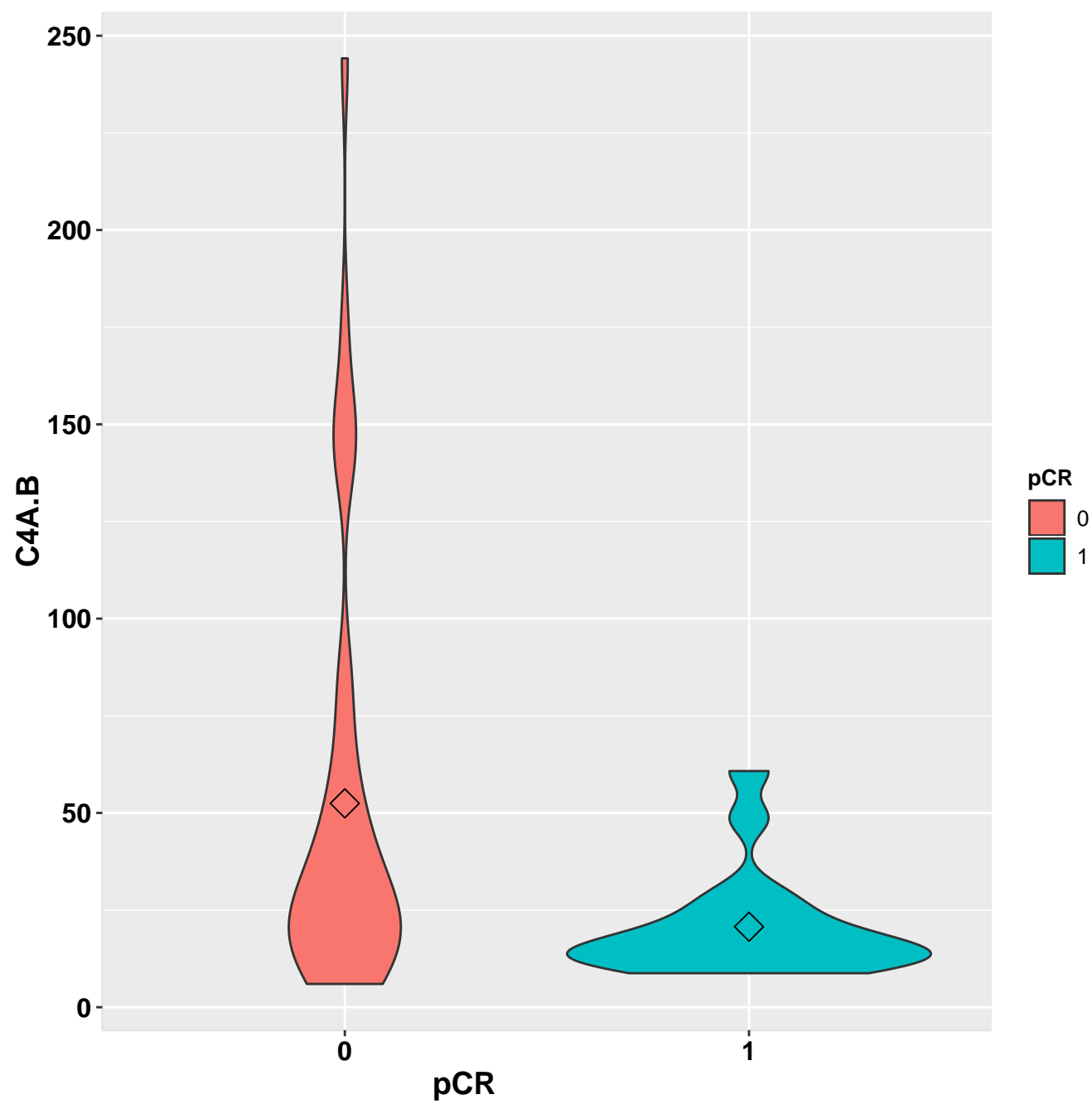

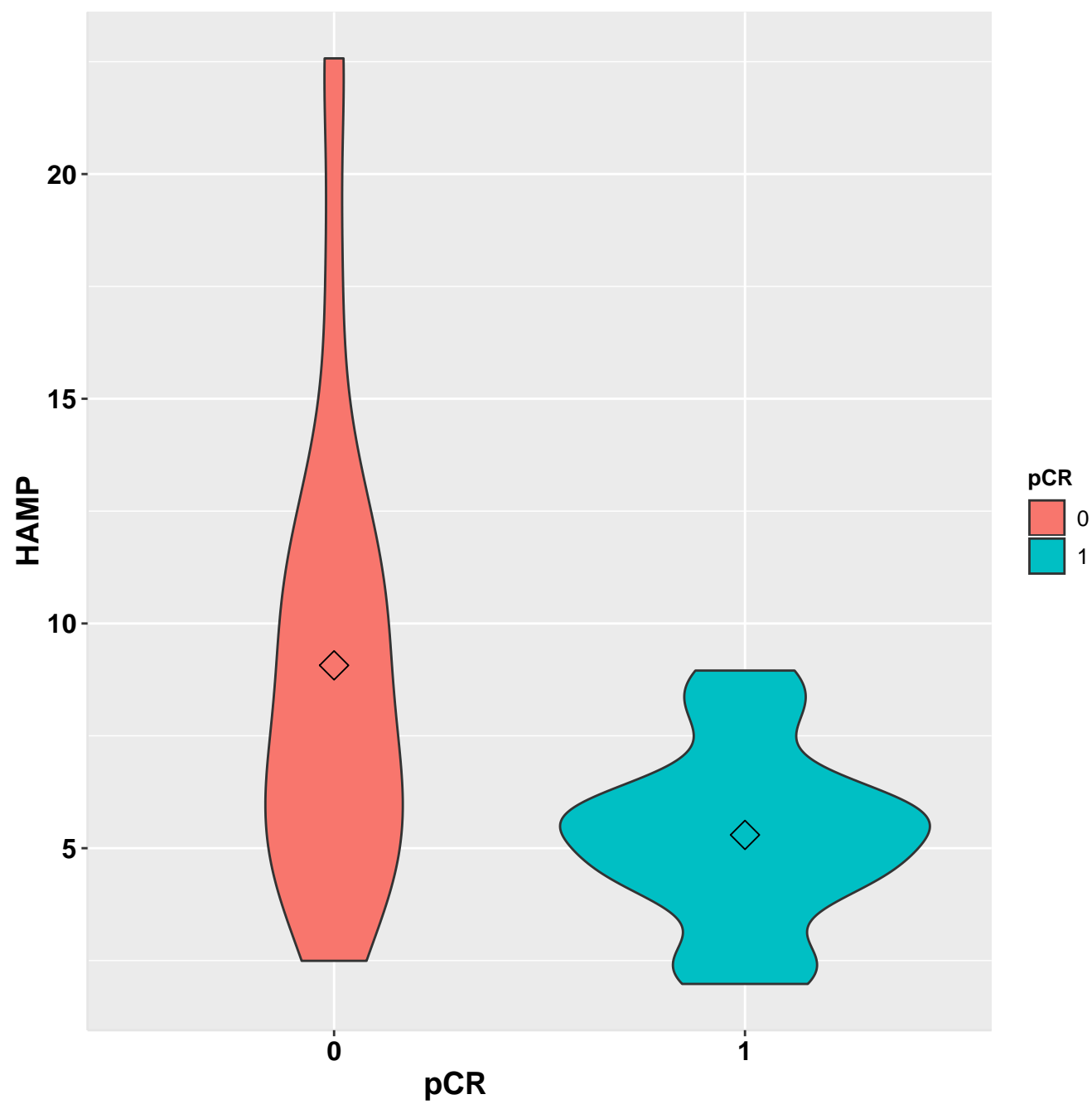

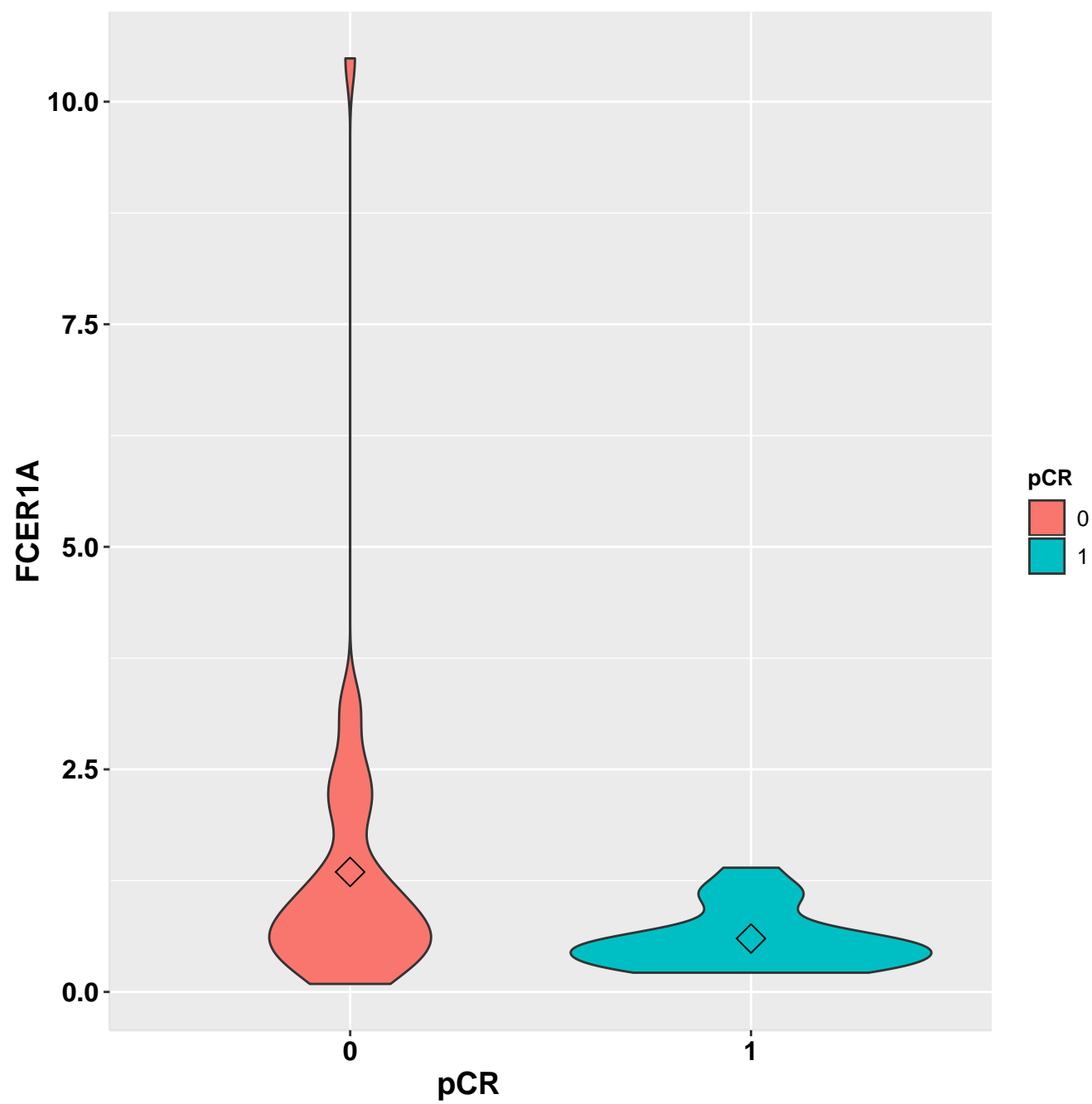

Supplement: Supplementary file 3 — Additional file 3. Violin plots for nine DEGs in the pCR model. Figure S1. Violin plots of nine pCR DEGs. IL2RA, CCL5, SELE, CCL20, CD7, genes have high expression in pCR. FCER1A, CD1A, HAMP, C4A.B genes have high expression in non-pCR. [file 12885_2020_7399_MOESM3_ESM.pdf]

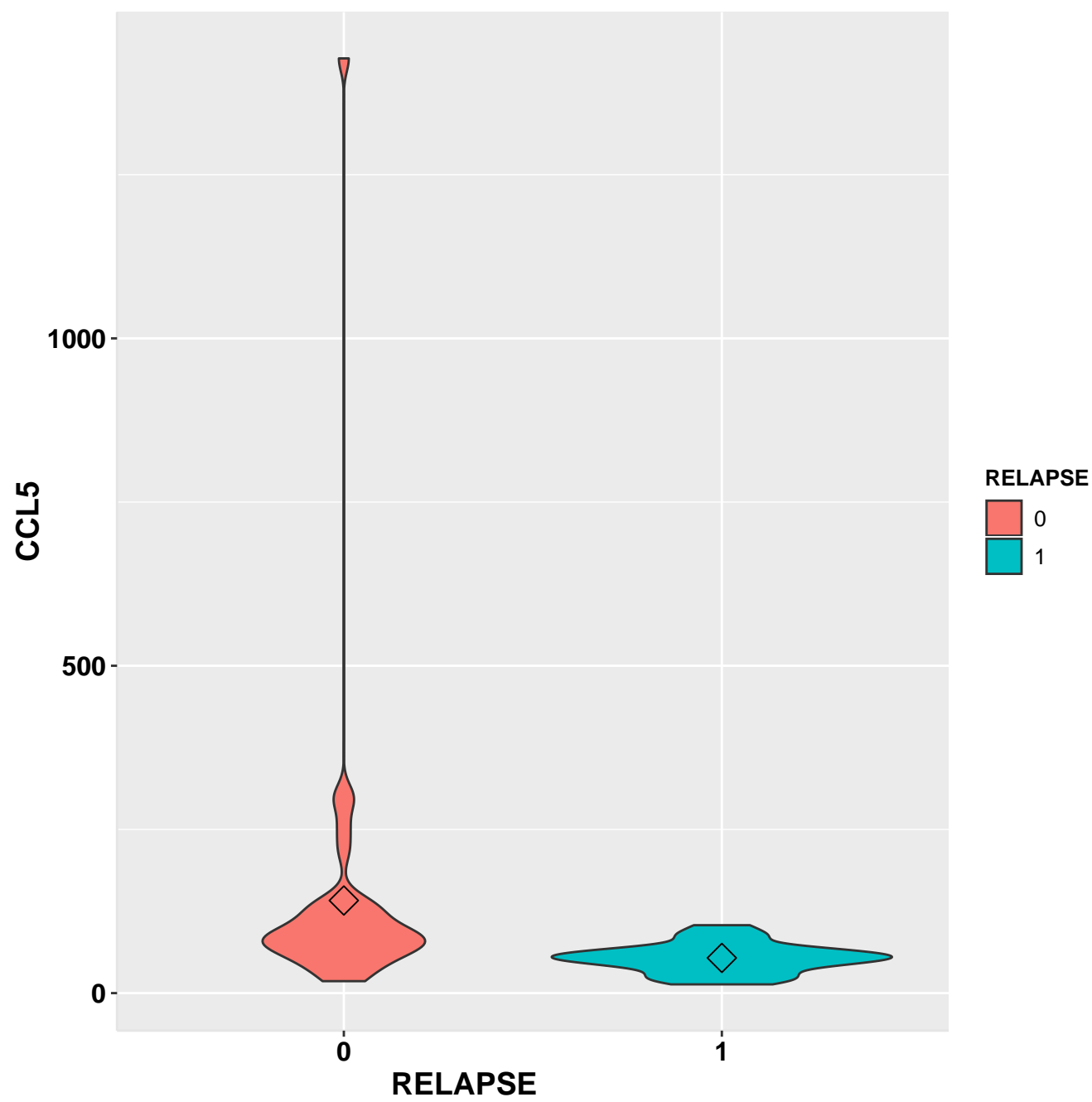

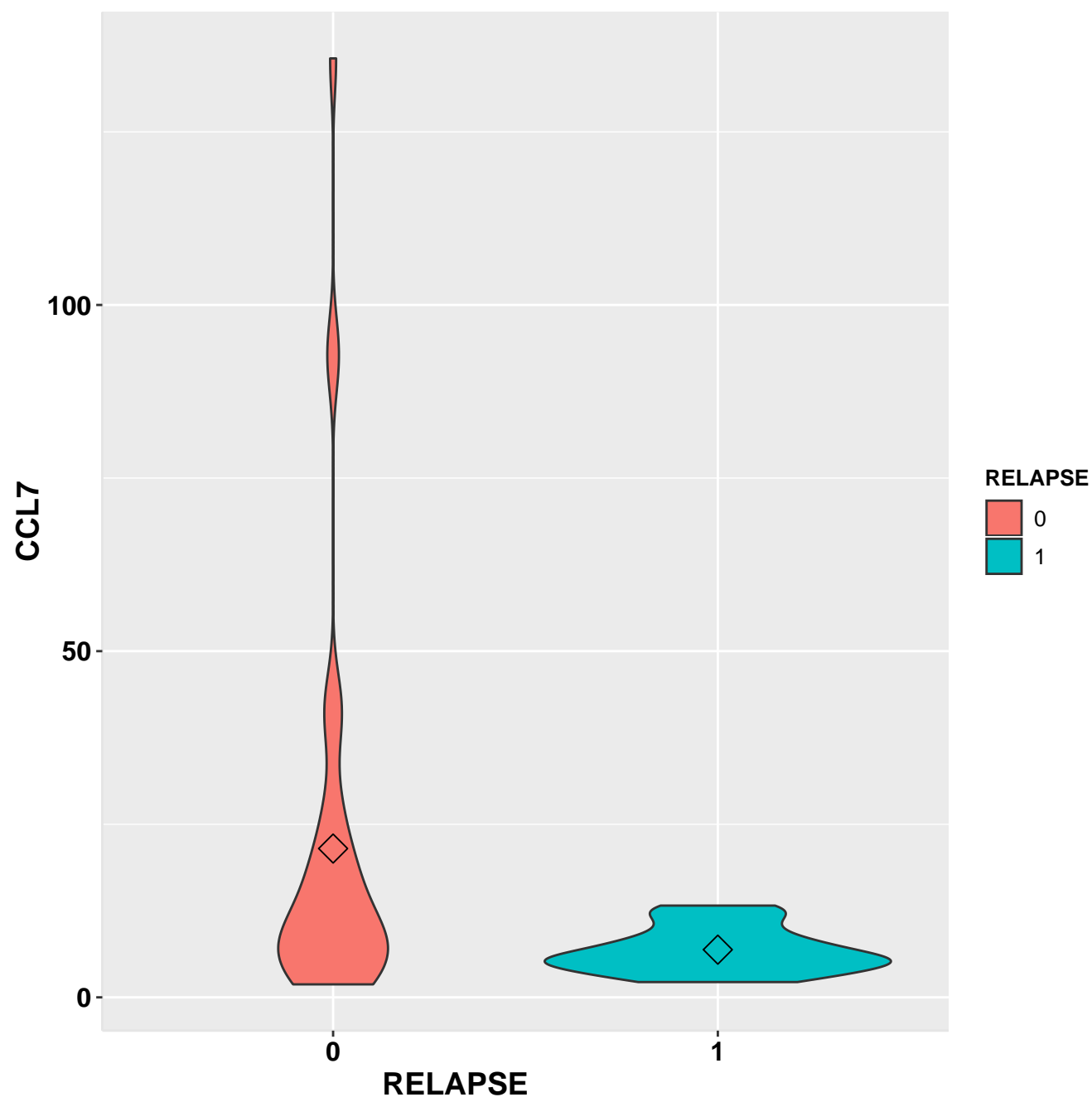

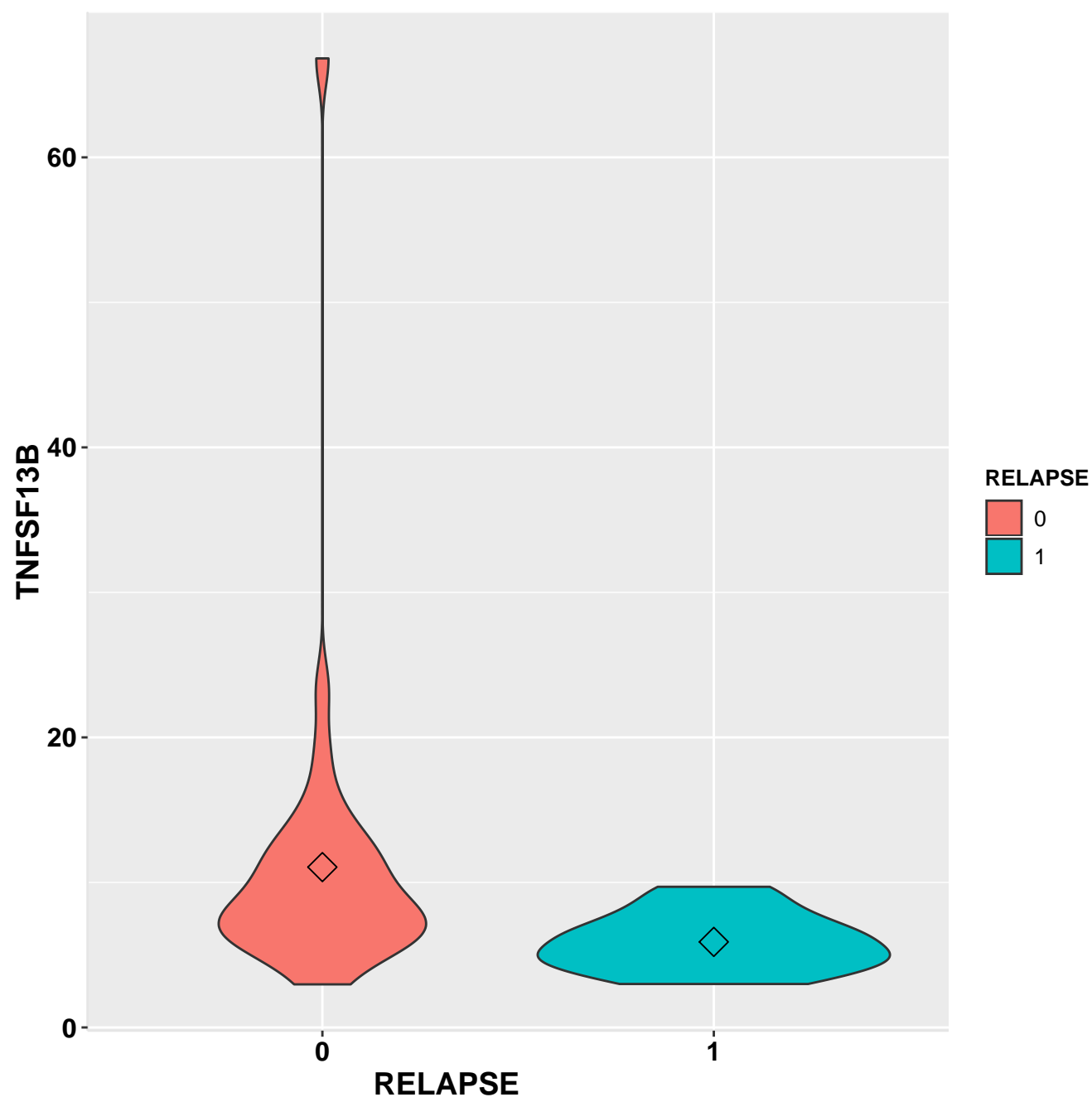

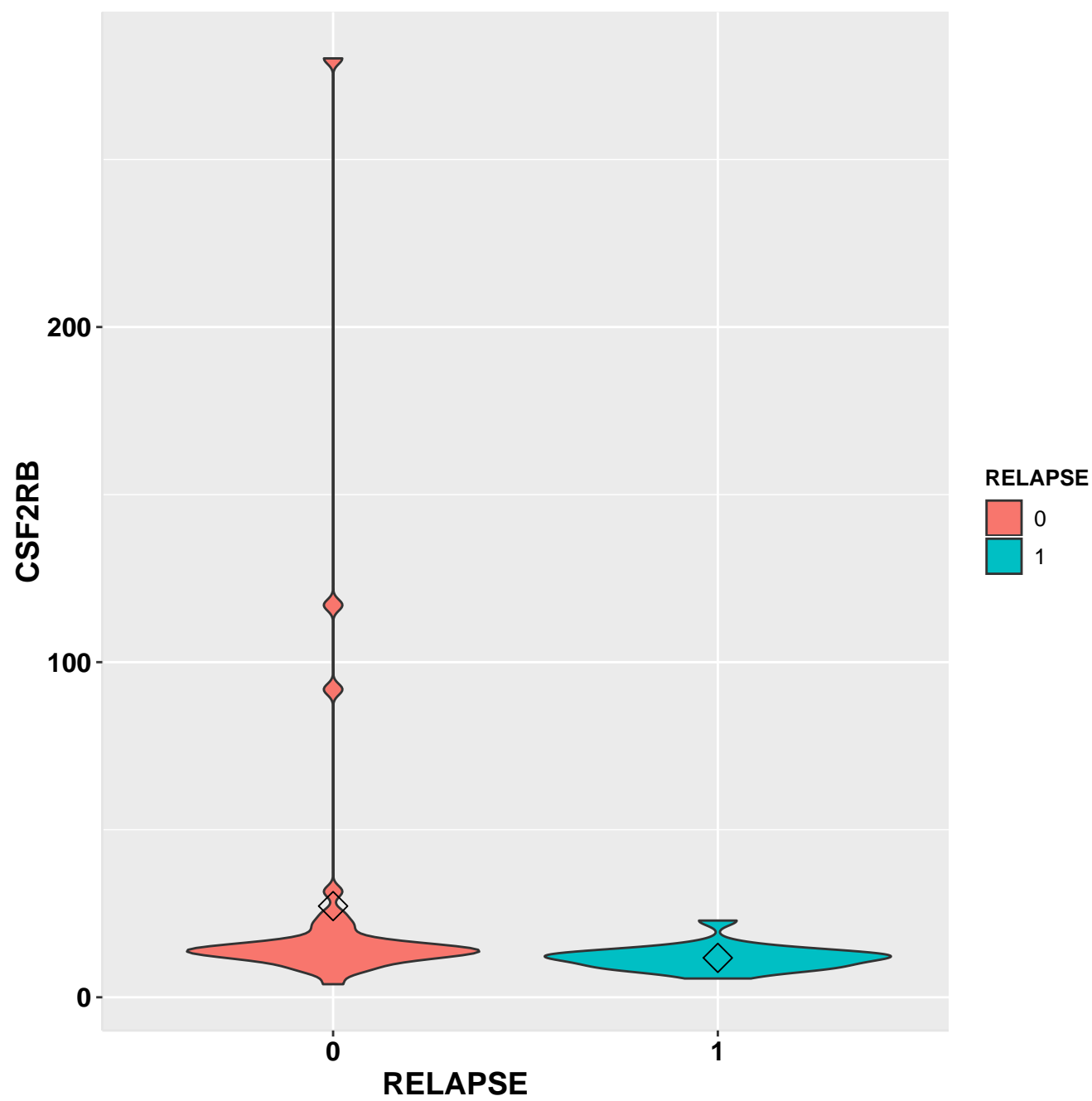

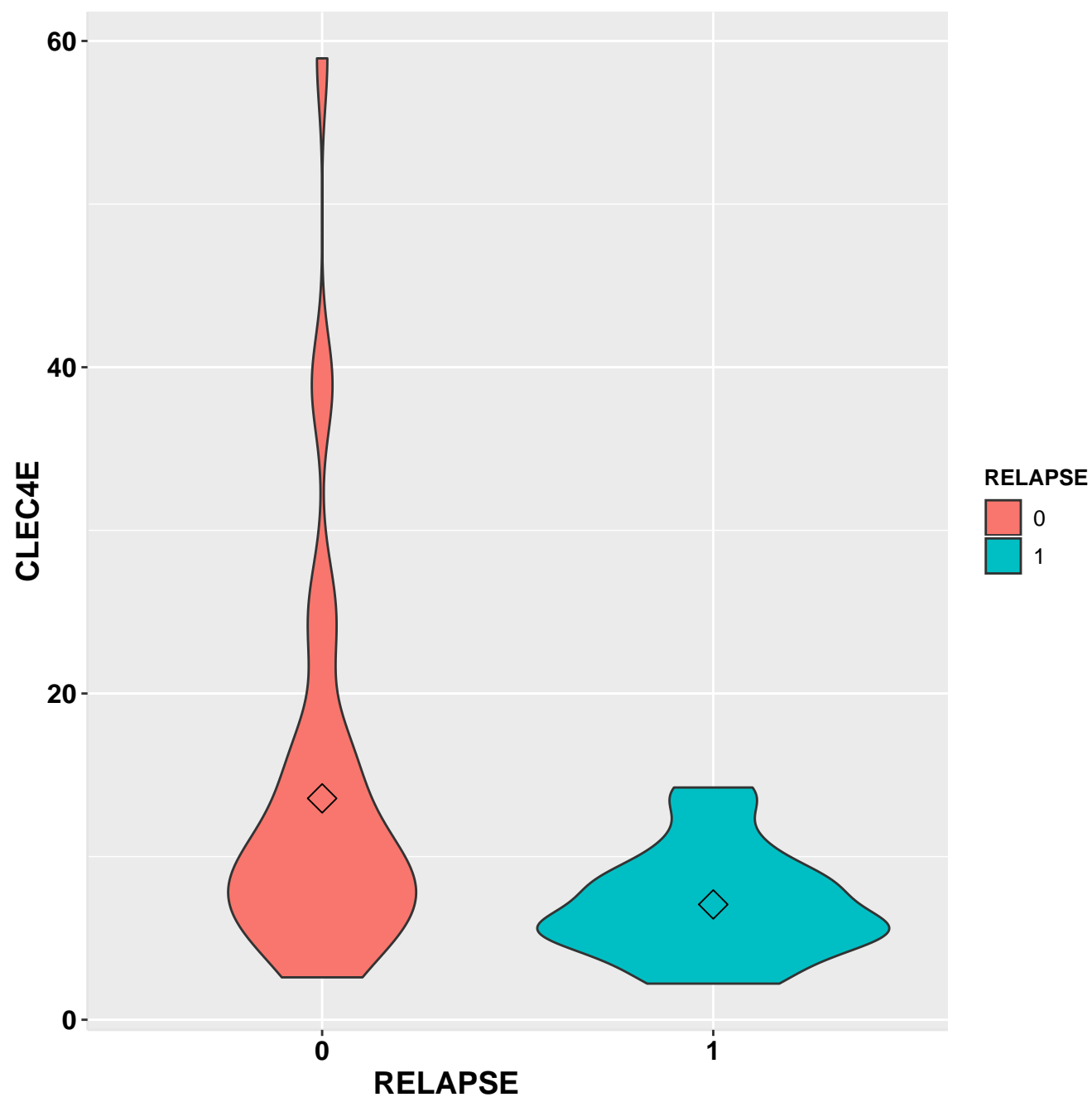

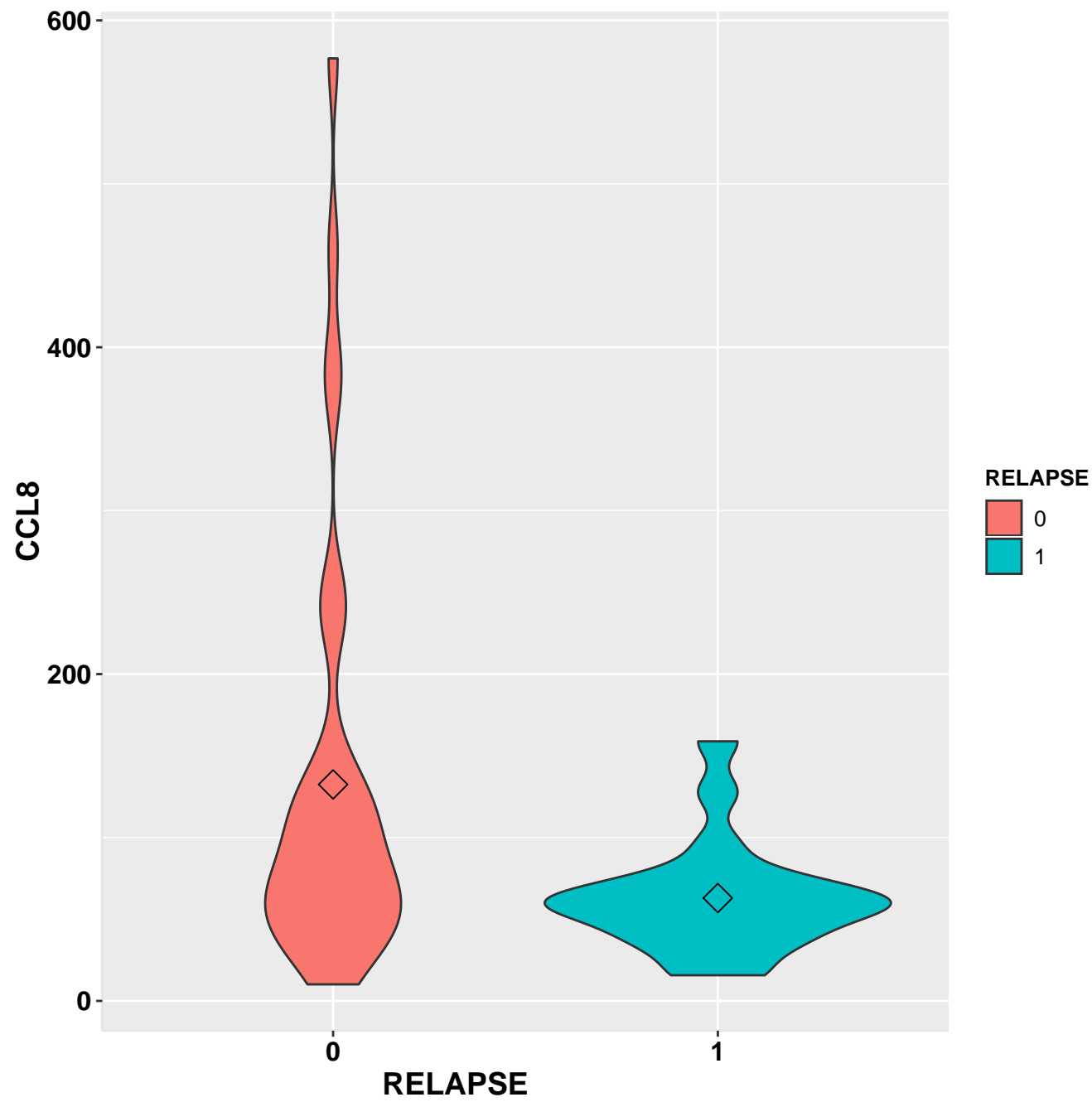

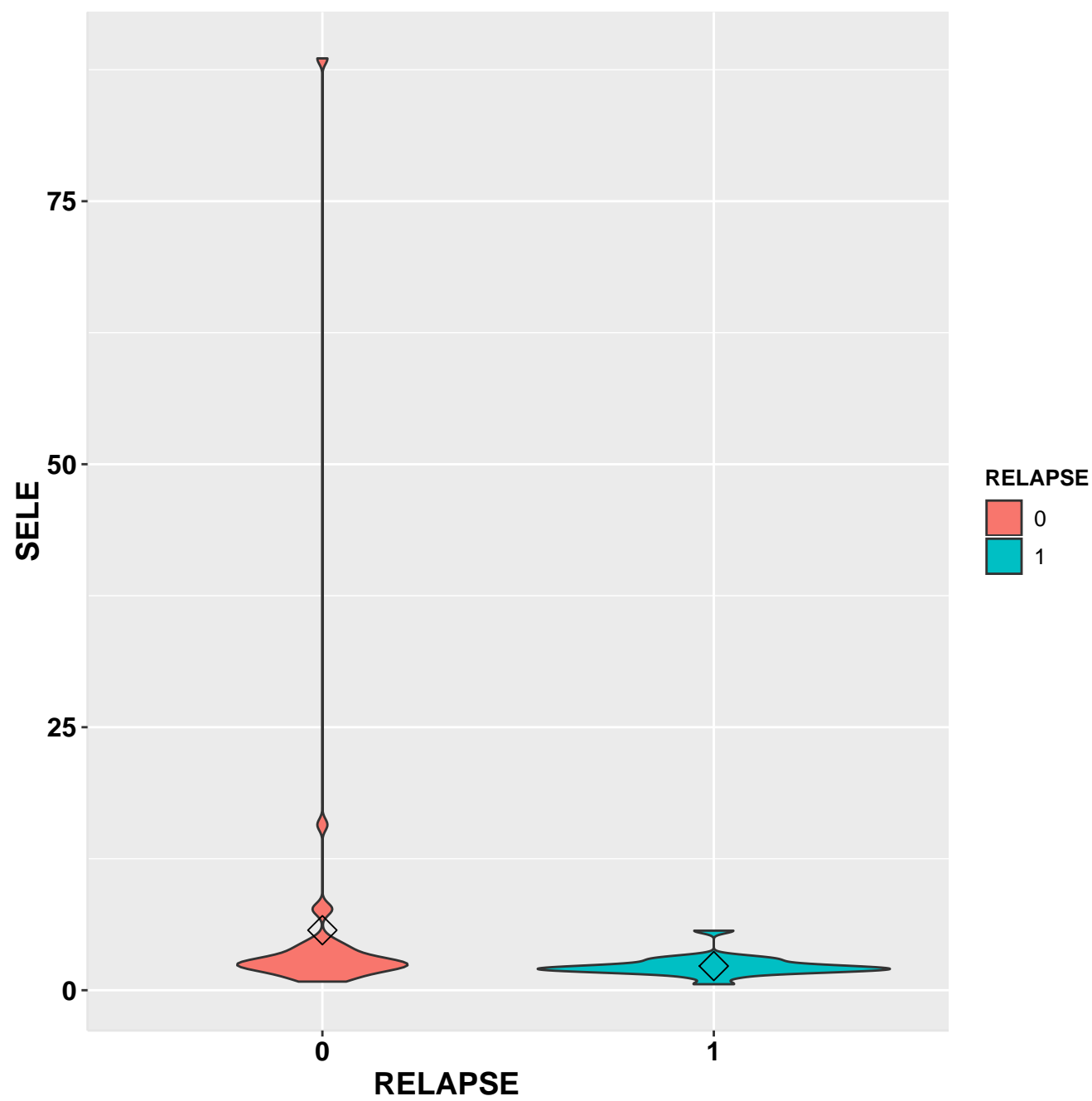

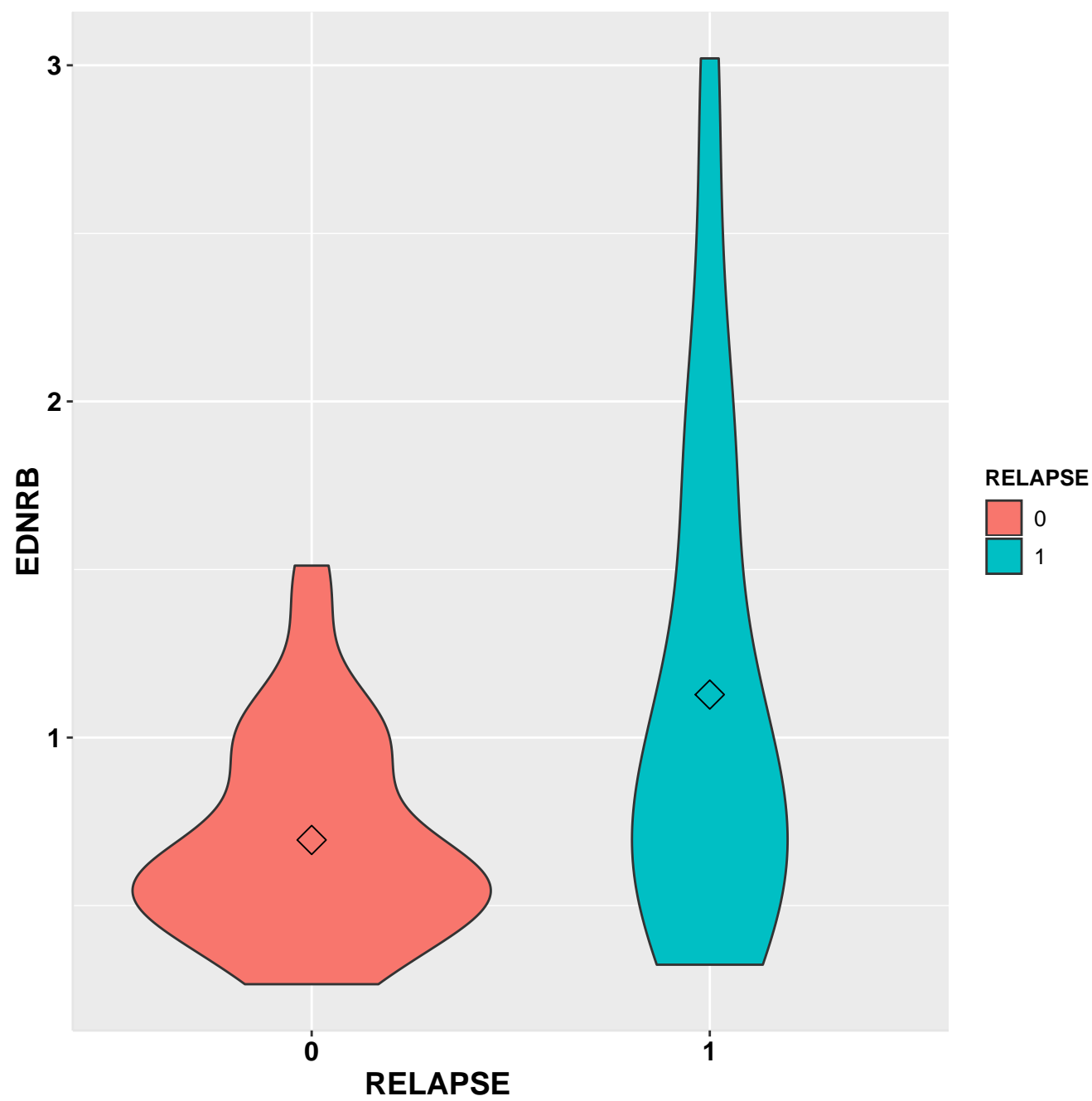

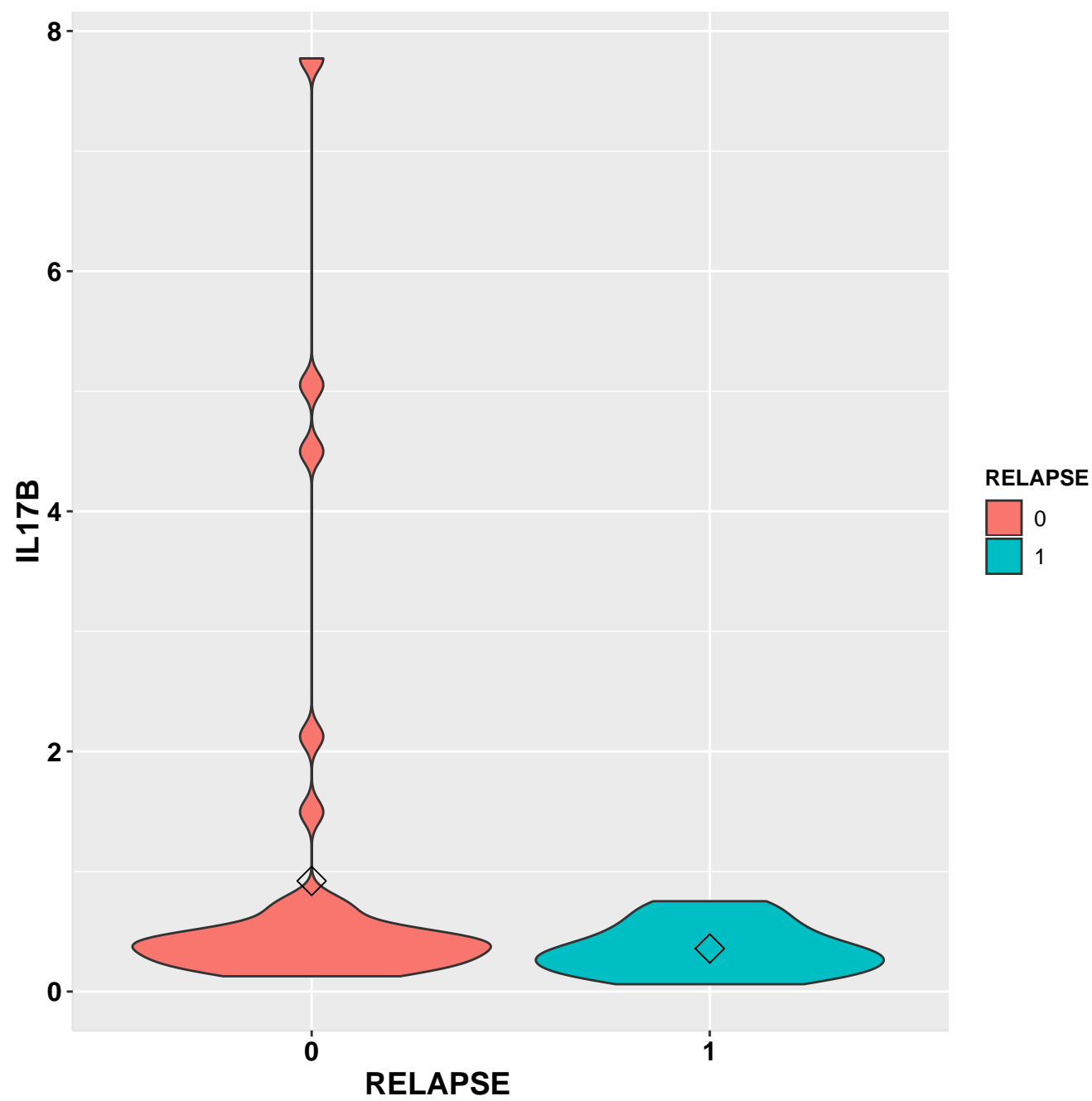

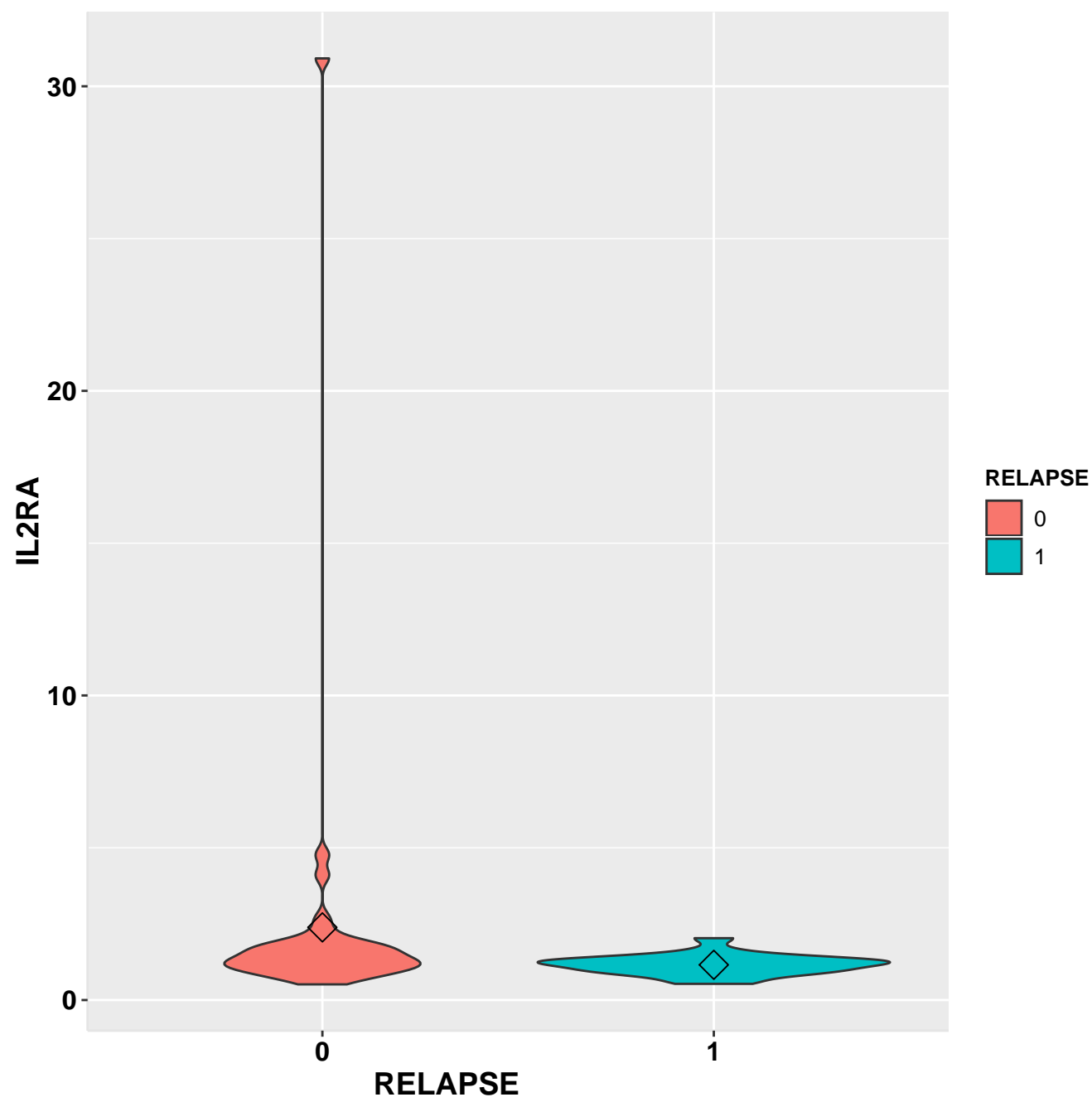

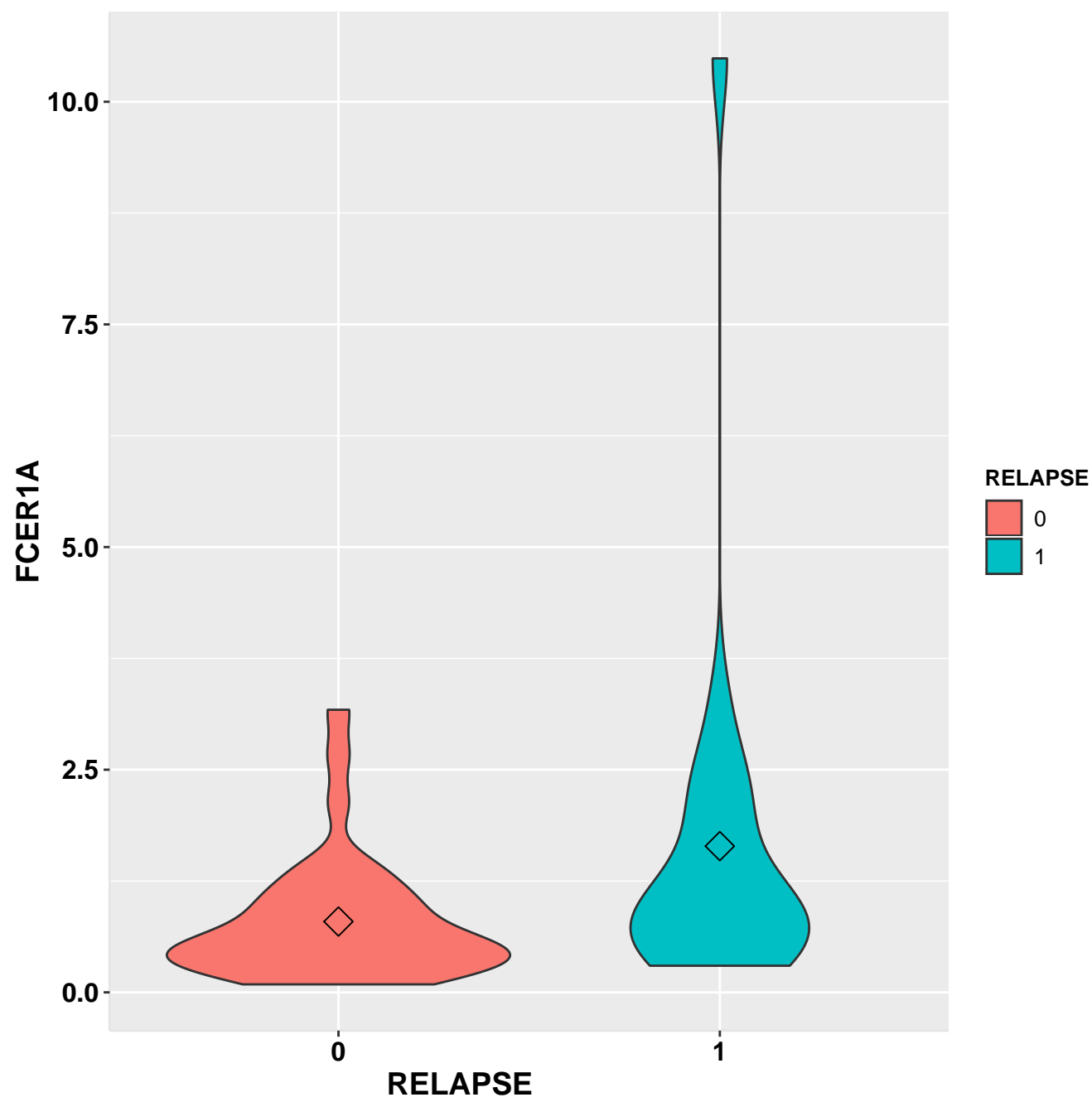

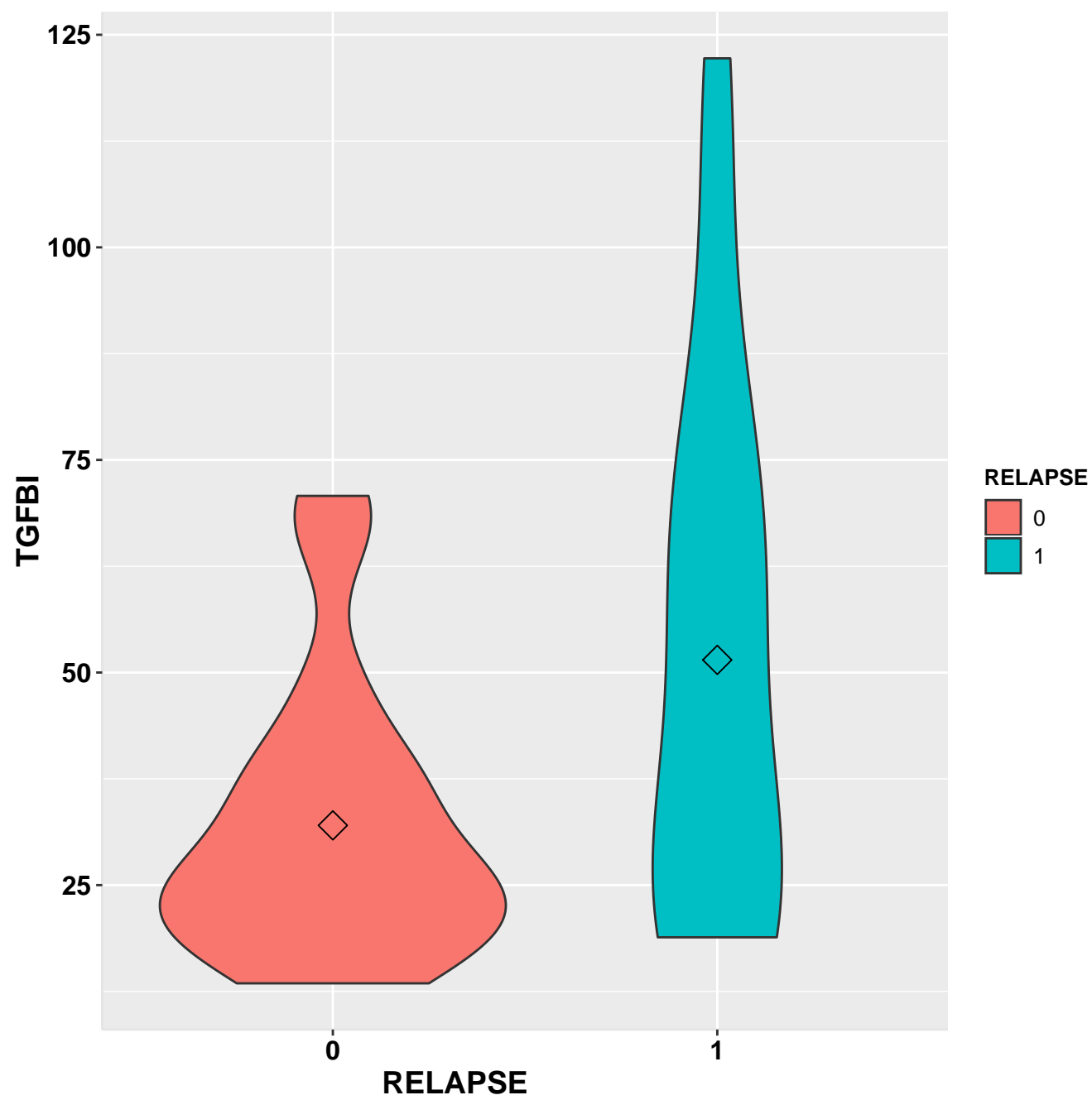

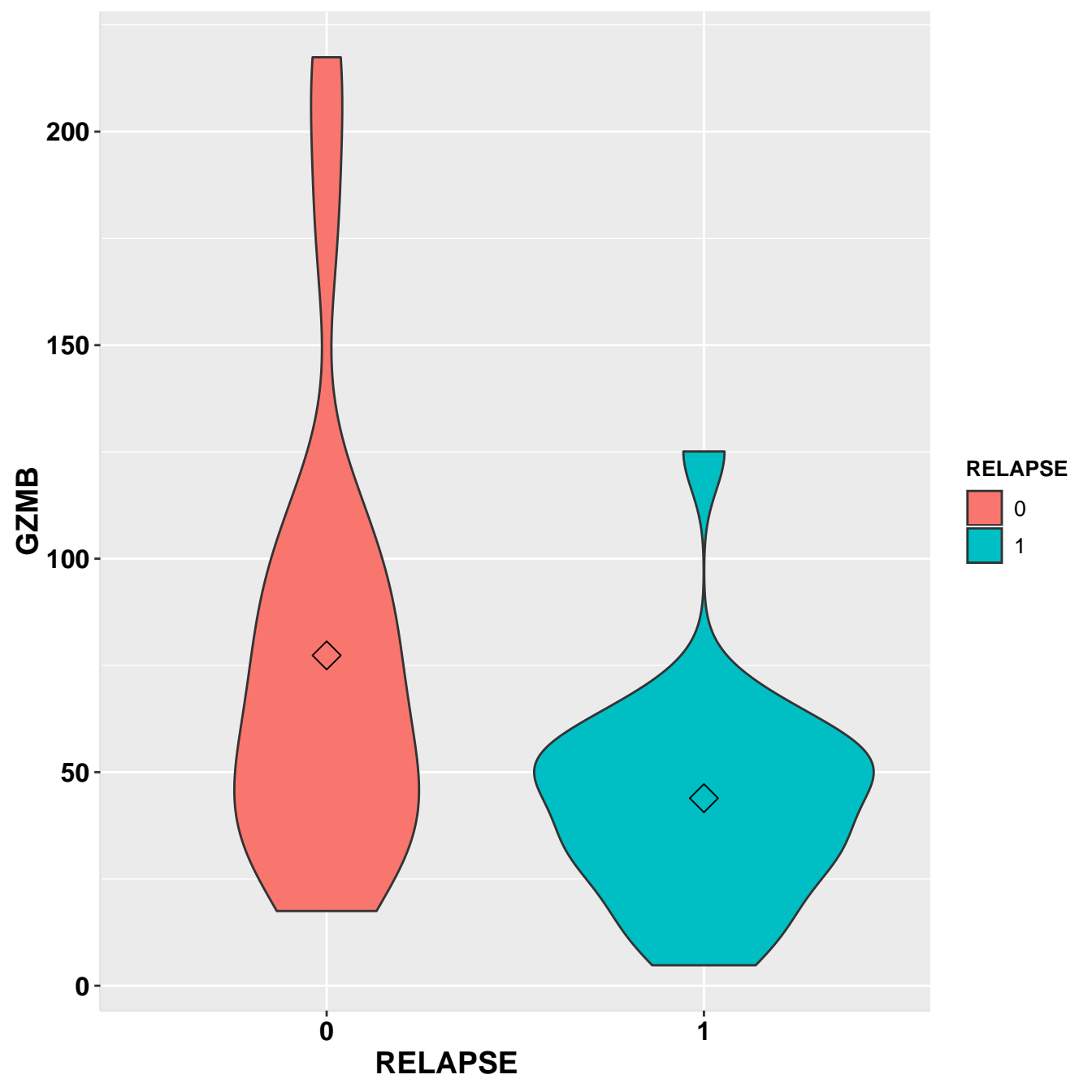

Supplement: Supplementary file 4 — Additional file 4. Violin pots for 13 DEGs in the relapse model. Figure S1. Violin plots of 13 relapse DEGs. CCL5, CCL7, TNFSF13B, CSF2RB, CLEC4E, CCL8, SELE, IL17B, IL2RA, and GZMB genes have high expression in non-RELAPSE. FCER1A, EDNRB, TGFBI genes have high expression in relapse. [file 12885_2020_7399_MOESM4_ESM.pdf]
