# Supplementary material for: Prediction of prognostic signatures in triple-negative breast cancer based on the differential expression analysis via NanoString nCounter immune panel
Source: BMC Cancer. 2020 Nov 2;20:1052. doi: 10.1186/s12885-020-07399-8 (PMC7607642; doi:10.1186/s12885-020-07399-8)
Supplement: Supplementary file 5 — Additional file 5. Clinical data for all patients. Table S1: Clinical data for all patients. Table S2. Spearman correlation between immunohistochemistry staining and gene expression quantified using the NanoString nCounter platform. [file 12885_2020_7399_MOESM5_ESM.pdf]

Table S1. Clinical Data

samid	Miller. Payne_grade	pCR	RCB	RELAPSE	DEATH	year_time	Pre/Post	Post. pCR	Post. Relapse
1	5	1	0	0	0	7.6942	Post	1	
2	5	1	0	0	0	7.5975	Post	1	
3	5	1	0	0	0	6.6000	Pre		
4	5	1	0	0	0	6.5558	Post	1	
5	5	1	0	0	0	6.3308	Post	1	
6	5	1	0	0	0	5.8475	Pre		
7	5	1	0	0	0	8.0858	Pre		
8	5	1	0	0	0	8.0808	Pre		
9	4	1	0	0	0	7.5225	Post	1	
10	5	1	0	0	0	7.2917	Pre		
11	5	1	0	0	0	7.2417	Pre		
12	5	1	0	0	0	7.0692	Pre		
13	5	1	0	0	0	7.4025	Pre		
14	5	1	0	0	0	6.2558	Pre		
15	5	1	0	0	0	6.1808	Pre		
16	5	1	0	0	0	5.7775	Pre		
17	5	1	0	0	0	5.4725	Post	1	
18	2	0	4.299	1	1	2.3000	Post	0	1
19	3	0	3.972	0	0	7.4275	Post	0	0
20	3	0	2.088	0	0	8.1192	Post	0	0
21	4	0	1.547	0	0	8.0275	Pre		
22	2	0	2.593	0	0	7.8058	Pre		
23	3	0	3.088	1	1	3.4475	Pre		
24	4	0	2.177	0	0	6.7692	Pre		
25	4	0	1.925	0	0	5.7442	Pre		
26	2	0	4.355	1	1	1.9192	Pre		
27	4	0	1.294	1	1	3.7667	Pre		
28	2	0	2.981	1	1	1.4392	Pre		
29	3	0	2.252	1	1	3.0775	Pre		
30	3	0	1.695	0	0	8.0725	Pre		
31	3	0	3.559	1	1	1.0308	Post	0	1
32	2	0	2.181	1	1	1.5942	Pre		
33	3	0	1.812	0	0	7.9167	Pre		
34	3	0	2.117	1	1	2.6000	Pre		
35	5	1	3.171	0	0	7.8058	Pre		
36	2	0	4.232	1	1	1.1417	Pre		
37	4	0	1.625	0	0	7.5975	Pre		
38	3	0	4.083	1	1	2.2750	Pre		
39	4	0	2.832	1	1	2.1917	Pre		
40	3	0	2.804	0	0	7.3167	Pre		
41	4	0	2.676	1	1	2.5058	Pre		
42	4	0	1.67	0	0	7.4692	Pre		
43	3	0	3.128	1	1	1.0000	Pre		
44	3	0	1.985	0	0	6.8642	Post	0	0
45	3	0	1.586	0	0	6.6917	Pre		
46	4	0	1.503	1	1	3.6892	Post	0	1
47	4	0	1.48	0	0	6.2808	Pre		

samid	Miller. Payne_grade	pCR	RCB	RELAPSE	DEATH	year_time	Pre/Post	Post. pCR	Post. Relapse
48	2	0	4.494	1	1	1.5108	Post	0	1
49	4	0	1.395	0	0	6.7858	Pre		
50	3	0	3.416	1	1	1.3858	Pre		
51	5	1	1.724	0	1	4.6083	Post	0	0
52	2	0	2.745	1	1	1.8308	Pre		
53	2	0	4.313	1	1	1.7775	Pre		
54	2	0	3.455	0	0	5.8975	Pre		
55	4	0	1.381	1	1	3.9558	Pre		

Table S2. Spearman correlation between immunohistochemistry staining and gene expression quantified using the NanoString nCounter platform.

	Number of $CD3^+$ cells	*CD3D* gene	*CD3E* gene	Number of $CD8^+$ cells	*CD8A* gene	*CD8B* gene	Number of $CD20^+$ cells	*CD20* gene
TIL on H&E	0.599 (<0.001)	0.508 (<0.001)	0.509 (<0.001)	0.498 (<0.001)	0.432 (0.001)	0.254 (0.062)	0.469 (<0.001)	0.300 (0.026)
Number of $CD3^+$ cells		0.738 (<0.001)	0.752 (<0.001)	0.709 (<0.001)	0.656 (<0.001)	0.583 (<0.001)	0.716 (<0.001)	0.646 (<0.001)
CD3D gene			0.983 (<0.001)	0.702 (<0.001)	0.919 (<0.001)	0.808 (<0.001)	0.655 (<0.001)	0.720 (<0.001)
CD3E gene				0.675 (<0.001)	0.918 (<0.001)	0.807 (<0.001)	0.664 (<0.001)	0.744 (<0.001)
Number of $CD8^+$ cells					0.693 (<0.001)	0.623 (<0.001)	0.425 (0.001)	0.435 (0.001)
CD8A gene						0.855 (<0.001)	0.523 (<0.001)	0.599 (<0.001)
CD8B gene							0.478 (<0.001)	0.535 (<0.001)
Number of $CD20^+$ cells								0.761 (<0.001)

Yellow color stands for the IHC markers.

The numbers in parentheses stand for the p-value as per the Spearman correlation.
